# Supplementary material for: Healthy microbiome—moving towards functional interpretation
Source: Gigascience. 2025 Mar 21;14:giaf015. doi: 10.1093/gigascience/giaf015 (PMC11927397; doi:10.1093/gigascience/giaf015)

## Supplement

### Supplementary Table 1

“Core functions”: functions present in at least 80% of healthy individuals from the HMP2 and two validation cohorts

| **Core function name**  1CMET2-PWY: folate transformations III (E. coli)  ANAEROFRUCAT-PWY: homolactic fermentation  ANAGLYCOLYSIS-PWY: glycolysis III (from glucose)  ARGININE-SYN4-PWY: L-ornithine biosynthesis II  ARGSYN-PWY: L-arginine biosynthesis I (via L-ornithine)  ARGSYNBSUB-PWY: L-arginine biosynthesis II (acetyl cycle)  ARO-PWY: chorismate biosynthesis I  ASPASN-PWY: superpathway of L-aspartate and L-asparagine biosynthesis  BIOTIN-BIOSYNTHESIS-PWY: biotin biosynthesis I  BRANCHED-CHAIN-AA-SYN-PWY: superpathway of branched chain amino acid biosynthesis  CALVIN-PWY: Calvin-Benson-Bassham cycle  CITRULBIO-PWY: L-citrulline biosynthesis  COA-PWY-1: superpathway of coenzyme A biosynthesis III (mammals)  COA-PWY: coenzyme A biosynthesis I (prokaryotic)  COBALSYN-PWY: superpathway of adenosylcobalamin salvage from cobinamide I  COLANSYN-PWY: colanic acid building blocks biosynthesis  COMPLETE-ARO-PWY: superpathway of aromatic amino acid biosynthesis  DTDPRHAMSYN-PWY: dTDP-&beta;-L-rhamnose biosynthesis  FASYN-ELONG-PWY: fatty acid elongation -- saturated  FERMENTATION-PWY: mixed acid fermentation  FUCCAT-PWY: fucose degradation  GALACTUROCAT-PWY: D-galacturonate degradation I  GLCMANNANAUT-PWY: superpathway of N-acetylglucosamine, N-acetylmannosamine and N-acetylneuraminate degradation  GLUCONEO-PWY: gluconeogenesis I  GLUCUROCAT-PWY: superpathway of &beta;-D-glucuronosides degradation  GLUTORN-PWY: L-ornithine biosynthesis I  GLYCOCAT-PWY: glycogen degradation I  GLYCOGENSYNTH-PWY: glycogen biosynthesis I (from ADP-D-Glucose)  GLYCOLYSIS-E-D: superpathway of glycolysis and the Entner-Doudoroff pathway  GLYCOLYSIS: glycolysis I (from glucose 6-phosphate)  HISDEG-PWY: L-histidine degradation I  HISTSYN-PWY: L-histidine biosynthesis  HSERMETANA-PWY: L-methionine biosynthesis III  ILEUSYN-PWY: L-isoleucine biosynthesis I (from threonine)  NAGLIPASYN-PWY: lipid IVA biosynthesis (E. coli)  NONMEVIPP-PWY: methylerythritol phosphate pathway I  NONOXIPENT-PWY: pentose phosphate pathway (non-oxidative branch) I  OANTIGEN-PWY: O-antigen building blocks biosynthesis (E. coli)  P41-PWY: pyruvate fermentation to acetate and (S)-lactate I  PANTO-PWY: phosphopantothenate biosynthesis I  PANTOSYN-PWY: superpathway of coenzyme A biosynthesis I (bacteria)  PENTOSE-P-PWY: pentose phosphate pathway  PEPTIDOGLYCANSYN-PWY: peptidoglycan biosynthesis I (meso-diaminopimelate containing)  PHOSLIPSYN-PWY: superpathway of phospholipid biosynthesis I (bacteria)  POLYISOPRENSYN-PWY: polyisoprenoid biosynthesis (E. coli)  PWY-1042: glycolysis IV  PWY-1269: CMP-3-deoxy-D-manno-octulosonate biosynthesis  PWY-2941: L-lysine biosynthesis II  PWY-2942: L-lysine biosynthesis III  PWY-3001: superpathway of L-isoleucine biosynthesis I  PWY-3841: folate transformations II (plants)  PWY-4984: urea cycle  PWY-5030: L-histidine degradation III  PWY-5097: L-lysine biosynthesis VI  PWY-5100: pyruvate fermentation to acetate and lactate II  PWY-5103: L-isoleucine biosynthesis III  PWY-5121: superpathway of geranylgeranyl diphosphate biosynthesis II (via MEP)  PWY-5130: 2-oxobutanoate degradation I  PWY-5154: L-arginine biosynthesis III (via N-acetyl-L-citrulline)  PWY-5188: tetrapyrrole biosynthesis I (from glutamate)  PWY-5384: sucrose degradation IV (sucrose phosphorylase)  PWY-5484: glycolysis II (from fructose 6-phosphate)  PWY-5505: L-glutamate and L-glutamine biosynthesis  PWY-5659: GDP-mannose biosynthesis  PWY-5667: CDP-diacylglycerol biosynthesis I  PWY-5686: UMP biosynthesis I  PWY-5695: inosine 5'-phosphate degradation  PWY-5941: glycogen degradation II  PWY-5973: cis-vaccenate biosynthesis  PWY-5989: stearate biosynthesis II (bacteria and plants)  PWY-6121: 5-aminoimidazole ribonucleotide biosynthesis I  PWY-6122: 5-aminoimidazole ribonucleotide biosynthesis II  PWY-6123: inosine-5'-phosphate biosynthesis I  PWY-6124: inosine-5'-phosphate biosynthesis II  PWY-6125: superpathway of guanosine nucleotides de novo biosynthesis II  PWY-6126: superpathway of adenosine nucleotides de novo biosynthesis II  PWY-6147: 6-hydroxymethyl-dihydropterin diphosphate biosynthesis I  PWY-6151: S-adenosyl-L-methionine salvage I  PWY-6163: chorismate biosynthesis from 3-dehydroquinate  PWY-621: sucrose degradation III (sucrose invertase)  PWY-6270: isoprene biosynthesis I  PWY-6277: superpathway of 5-aminoimidazole ribonucleotide biosynthesis  PWY-6282: palmitoleate biosynthesis I (from (5Z)-dodec-5-enoate)  PWY-6292: superpathway of L-cysteine biosynthesis (mammalian)  PWY-6305: superpathway of putrescine biosynthesis  PWY-6317: D-galactose degradation I (Leloir pathway)  PWY-6353: purine nucleotides degradation II (aerobic)  PWY-6385: peptidoglycan biosynthesis III (mycobacteria)  PWY-6386: UDP-N-acetylmuramoyl-pentapeptide biosynthesis II (lysine-containing)  PWY-6387: UDP-N-acetylmuramoyl-pentapeptide biosynthesis I (meso-diaminopimelate containing)  PWY-6507: 4-deoxy-L-threo-hex-4-enopyranuronate degradation  PWY-6519: 8-amino-7-oxononanoate biosynthesis I  PWY-6527: stachyose degradation  PWY-6606: guanosine nucleotides degradation II  PWY-6608: guanosine nucleotides degradation III  PWY-6609: adenine and adenosine salvage III  PWY-6628: superpathway of L-phenylalanine biosynthesis  PWY-6630: superpathway of L-tyrosine biosynthesis  PWY-6700: queuosine biosynthesis I (de novo)  PWY-6703: preQ0 biosynthesis  PWY-6731: starch degradation III  PWY-6823: molybdopterin biosynthesis  PWY-6859: all-trans-farnesol biosynthesis  PWY-6897: thiamine diphosphate salvage II  PWY-6901: superpathway of glucose and xylose degradation  PWY-6902: chitin degradation II (Vibrio)  PWY-6936: seleno-amino acid biosynthesis (plants)  PWY-6969: TCA cycle V (2-oxoglutarate synthase)  PWY-702: L-methionine biosynthesis II  PWY-7111: pyruvate fermentation to isobutanol (engineered)  PWY-7197: pyrimidine deoxyribonucleotide phosphorylation  PWY-7199: pyrimidine deoxyribonucleosides salvage  PWY-7208: superpathway of pyrimidine nucleobases salvage  PWY-7220: adenosine deoxyribonucleotides de novo biosynthesis II  PWY-7221: guanosine ribonucleotides de novo biosynthesis  PWY-7222: guanosine deoxyribonucleotides de novo biosynthesis II  PWY-7228: superpathway of guanosine nucleotides de novo biosynthesis I  PWY-7229: superpathway of adenosine nucleotides de novo biosynthesis I  PWY-7234: inosine-5'-phosphate biosynthesis III  PWY-7237: myo-, chiro- and scyllo-inositol degradation  PWY-7238: sucrose biosynthesis II  PWY-7242: D-fructuronate degradation  PWY-724: superpathway of L-lysine, L-threonine and L-methionine biosynthesis II  PWY-7282: 4-amino-2-methyl-5-diphosphomethylpyrimidine biosynthesis II  PWY-7323: superpathway of GDP-mannose-derived O-antigen building blocks biosynthesis  PWY-7328: superpathway of UDP-glucose-derived O-antigen building blocks biosynthesis  PWY-7345: superpathway of anaerobic sucrose degradation  PWY-7357: thiamine phosphate formation from pyrithiamine and oxythiamine (yeast)  PWY-7383: anaerobic energy metabolism (invertebrates, cytosol)  PWY-7392: taxadiene biosynthesis (engineered)  PWY-7400: L-arginine biosynthesis IV (archaebacteria)  PWY-7456: &beta;-(1,4)-mannan degradation  PWY-7560: methylerythritol phosphate pathway II  PWY-7663: gondoate biosynthesis (anaerobic)  PWY-7664: oleate biosynthesis IV (anaerobic)  PWY-7761: NAD salvage pathway II (PNC IV cycle)  PWY-7790: UMP biosynthesis II  PWY-7791: UMP biosynthesis III  PWY-7851: coenzyme A biosynthesis II (eukaryotic)  PWY-7953: UDP-N-acetylmuramoyl-pentapeptide biosynthesis III (meso-diaminopimelate containing)  PWY-7977: L-methionine biosynthesis IV  PWY-8004: Entner-Doudoroff pathway I  PWY-8073: lipid IVA biosynthesis (P. putida)  PWY-8178: pentose phosphate pathway (non-oxidative branch) II  PWY-8187: L-arginine degradation XIII (reductive Stickland reaction)  PWY-841: superpathway of purine nucleotides de novo biosynthesis I  PWY-I9: L-cysteine biosynthesis VI (from L-methionine)  PWY0-1241: ADP-L-glycero-&beta;-D-manno-heptose biosynthesis  PWY0-1261: anhydromuropeptides recycling I  PWY0-1296: purine ribonucleosides degradation  PWY0-1319: CDP-diacylglycerol biosynthesis II  PWY0-1586: peptidoglycan maturation (meso-diaminopimelate containing)  PWY0-162: superpathway of pyrimidine ribonucleotides de novo biosynthesis  PWY0-845: superpathway of pyridoxal 5'-phosphate biosynthesis and salvage  PWY0-862: (5Z)-dodecenoate biosynthesis I  PWY4FS-7: phosphatidylglycerol biosynthesis I (plastidic)  PWY4FS-8: phosphatidylglycerol biosynthesis II (non-plastidic)  PWY66-399: gluconeogenesis III  PWY66-429: fatty acid biosynthesis initiation (mitochondria)  PYRIDNUCSYN-PWY: NAD de novo biosynthesis I (from aspartate)  PYRIDOXSYN-PWY: pyridoxal 5'-phosphate biosynthesis I  RHAMCAT-PWY: L-rhamnose degradation I  RIBOSYN2-PWY: flavin biosynthesis I (bacteria and plants)  SALVADEHYPOX-PWY: adenosine nucleotides degradation II  SER-GLYSYN-PWY: superpathway of L-serine and glycine biosynthesis I  THISYNARA-PWY: superpathway of thiamine diphosphate biosynthesis III (eukaryotes)  THRESYN-PWY: superpathway of L-threonine biosynthesis  TRNA-CHARGING-PWY: tRNA charging  UDPNAGSYN-PWY: UDP-N-acetyl-D-glucosamine biosynthesis I  VALSYN-PWY: L-valine biosynthesis |
| --- |

### Supplementary Figure 1

Number of observed functions per sample, separated by health group and project.


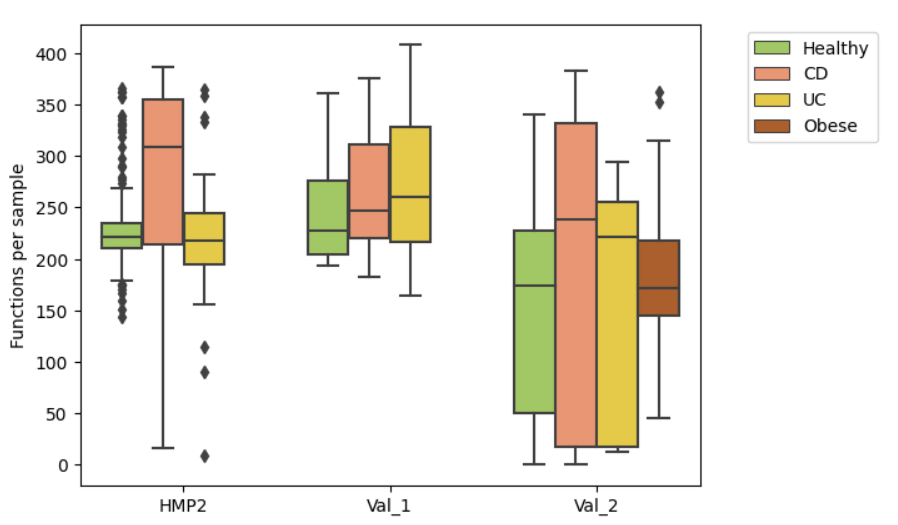


### Supplementary Figure 2

Comparison of core functions in health and disease. **a.** Overlap of LEfSe differentially enriched pathways in healthy versus unhealthy samples in the validation and HMP2 cohorts. **b.** Overlap of LEfSe differentially enriched pathways in unhealthy versus healthy samples in the validation and HMP2 cohorts**. c.** Fraction of seen core functions among all annotated core functions. **d.** Fraction of seen core functions among all functions seen in a sample.


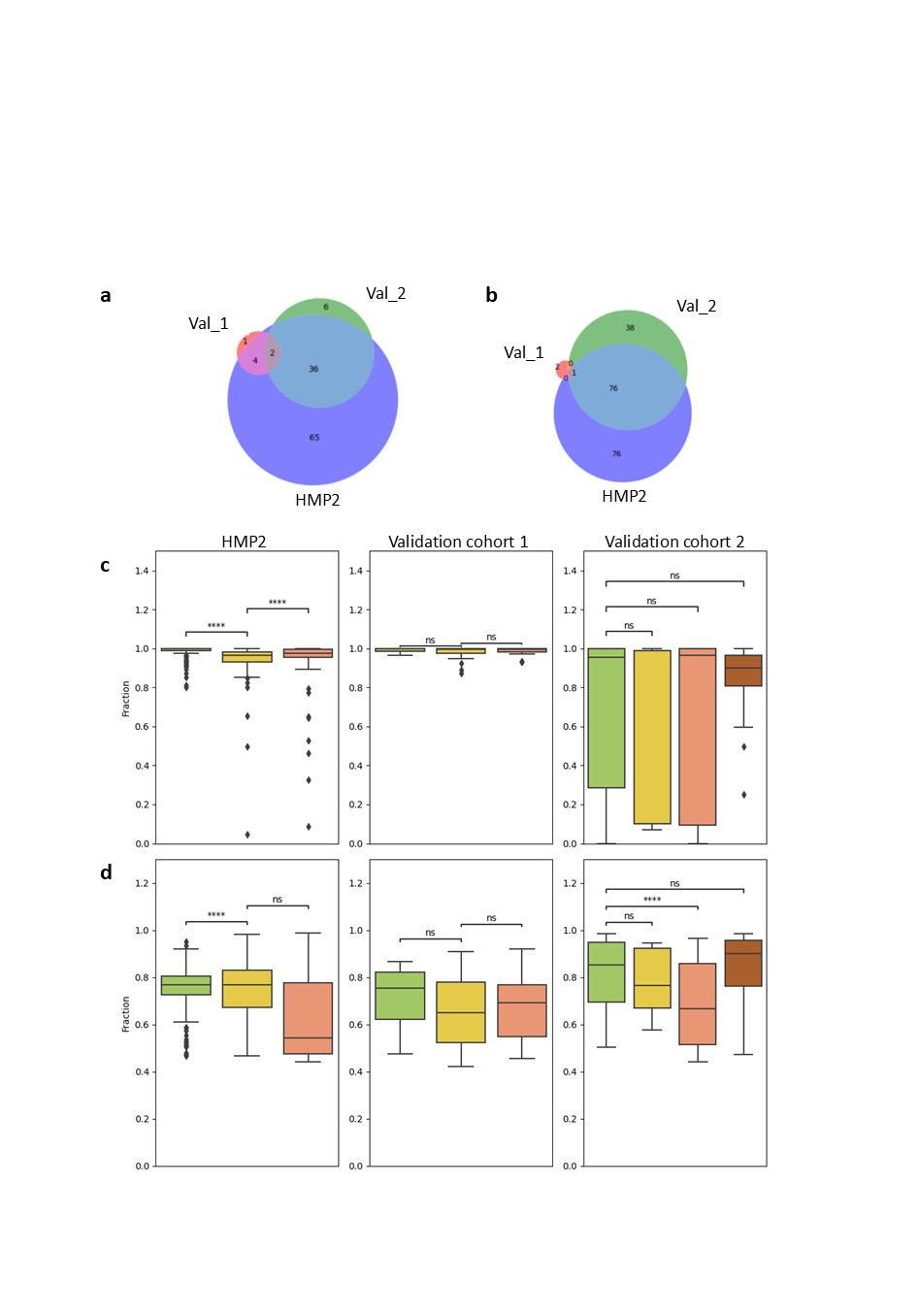


### Supplementary Figure 3

Number of observed species per sample, separated by health group and project.


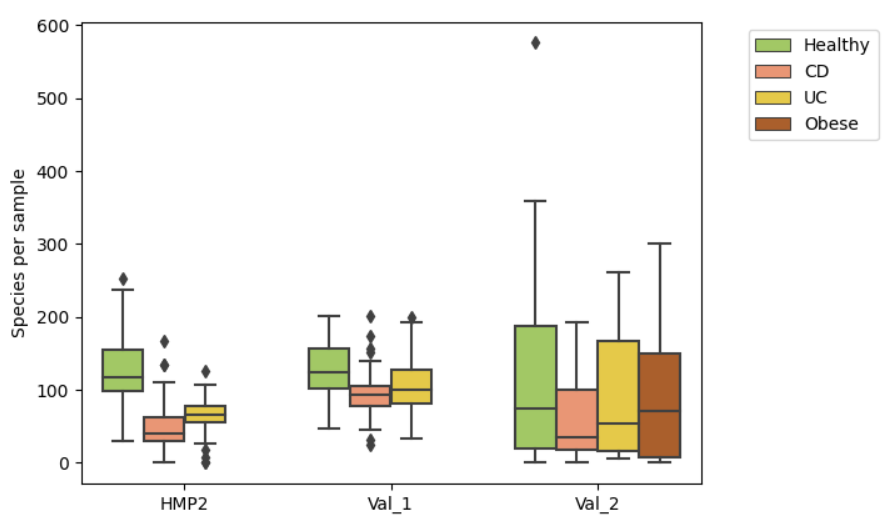


### Supplementary Figure 4

Top species contributions to core functions, based on the stratified output of HUMAnN. For every cohort, the sum of the contributions for all samples was calculated and contributions with the greatest summed abundances are shown. The final values have been normalized to values between 0 and 1 to compare the strength of the correlations between the groups.


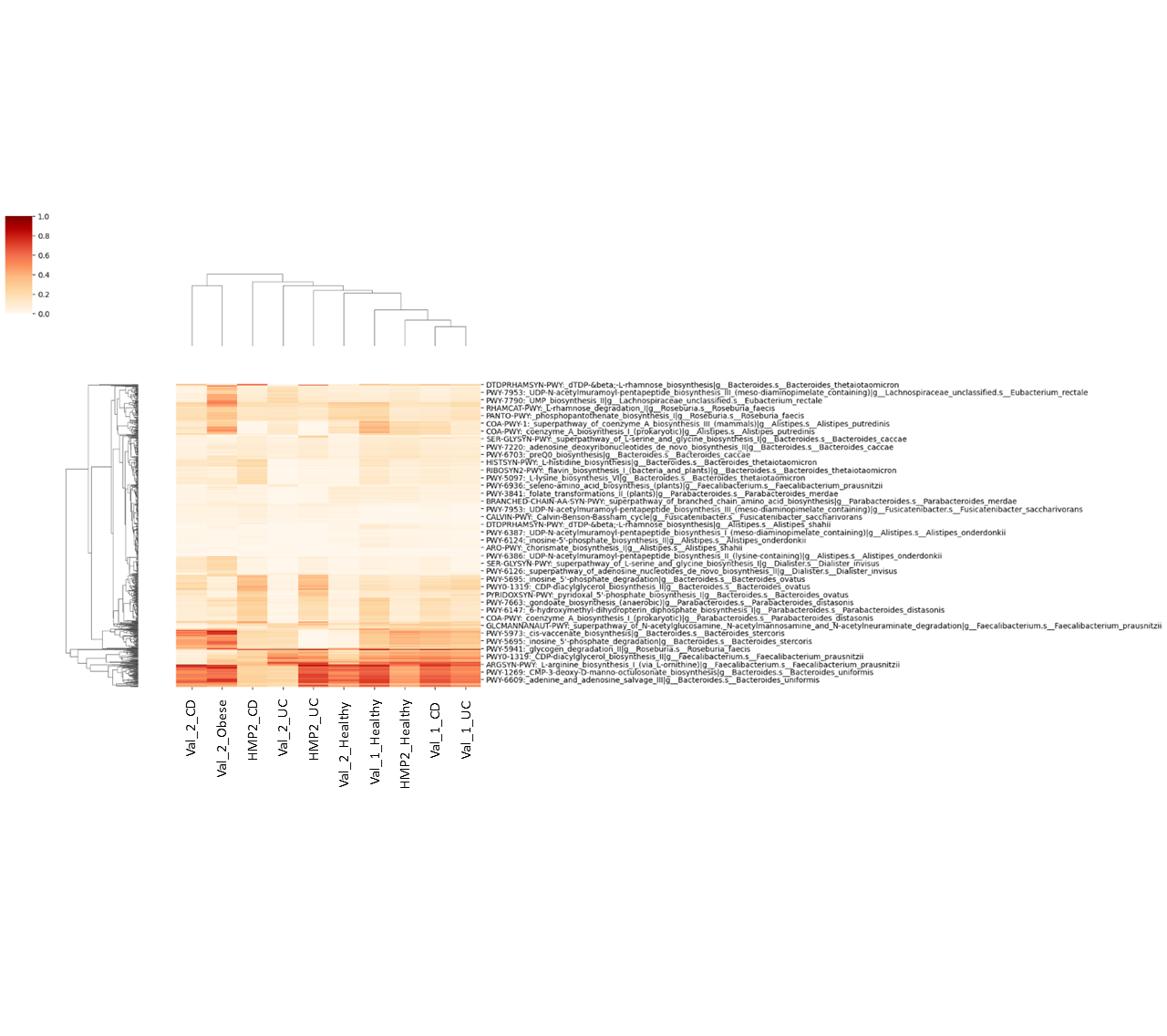


### Supplementary Figure 5

Functional redundancy. **a.** Average number of species per function in a sample. **b**. Average number of functions per species.


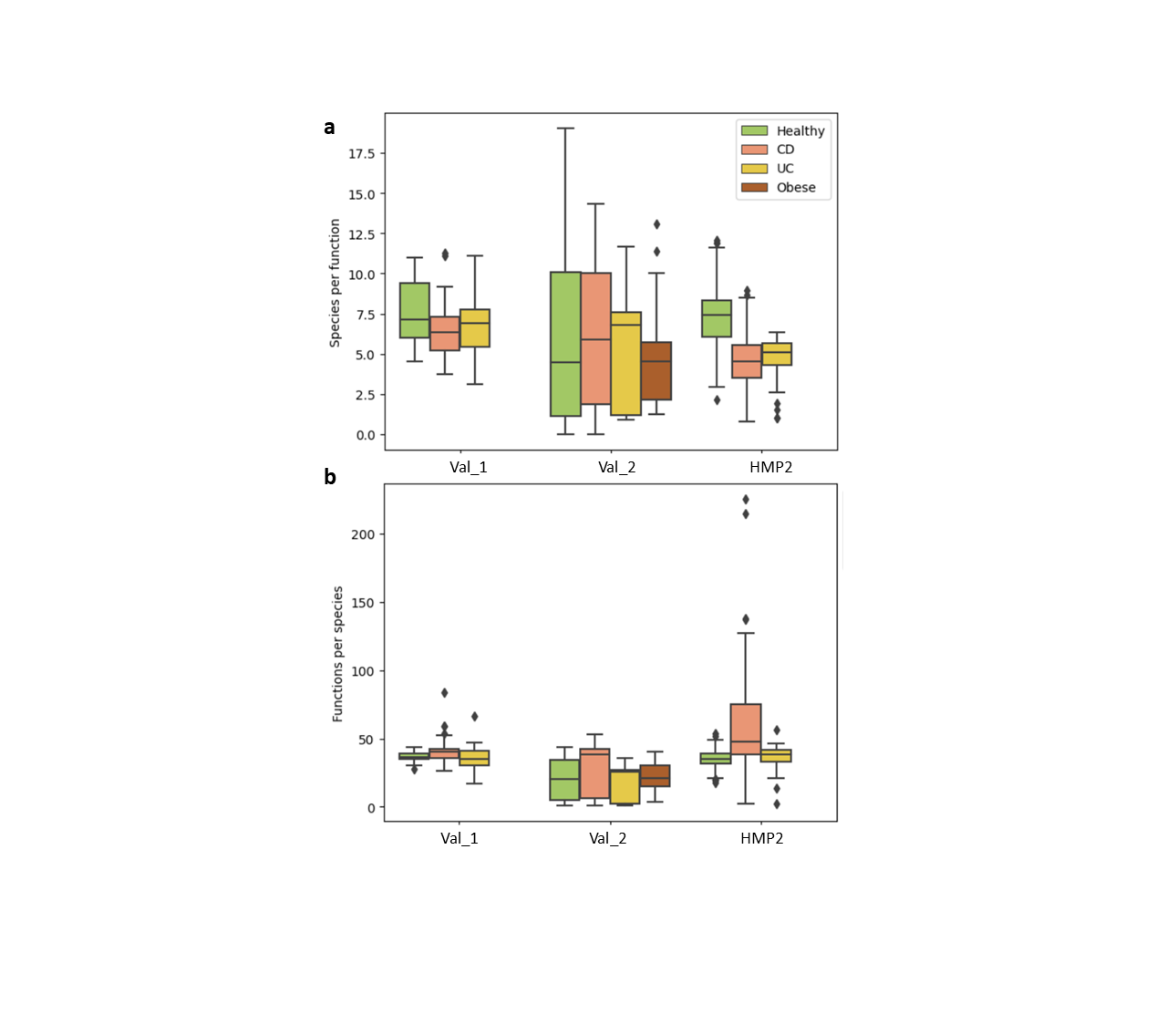


### Supplementary Figure 6

Accuracy and AUC scores achieved by each index, separately for each cohort.


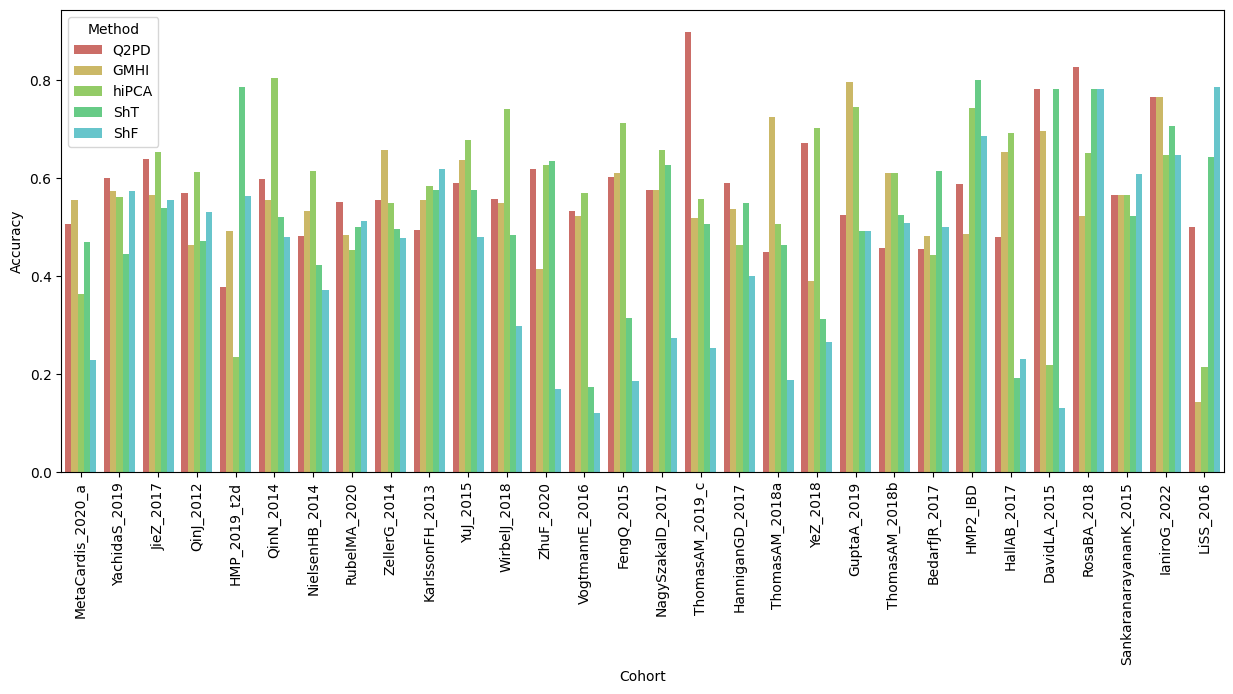


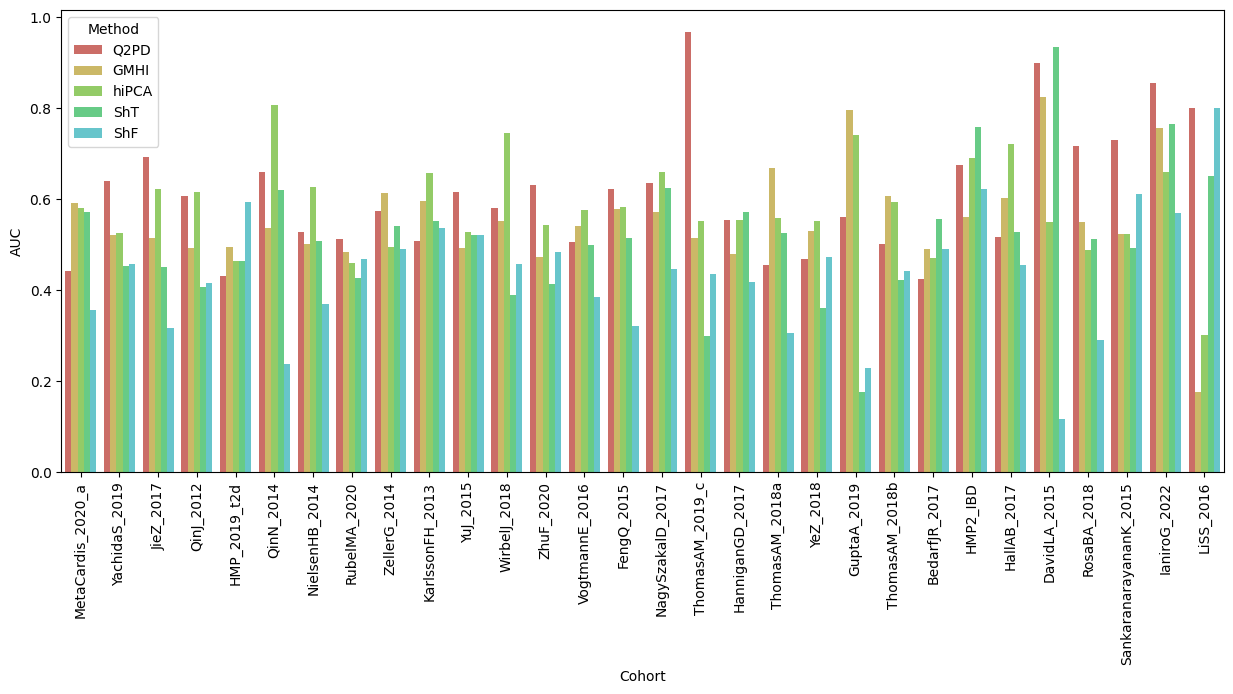

Supplement: giaf015_Supplemental_File [file giaf015_supplemental_file.docx]
