# Supplementary material for: Healthy microbiome—moving towards functional interpretation
Source: Gigascience. 2025 Mar 21;14:giaf015. doi: 10.1093/gigascience/giaf015 (PMC11927397; doi:10.1093/gigascience/giaf015)
Supplement: giaf015_GIGA-D-24-00311_Original_Submission [file giaf015_giga-d-24-00311_original_submission.pdf]

GigaScience

Healthy microbiome - moving towards functional interpretation

--Manuscript Draft--

**Manuscript Number:**	GIGA-D-24-00311
**Full Title:**	Healthy microbiome - moving towards functional interpretation
**Article Type:**	Research
**Funding Information:**	NCN Sonata BIS (2020/38/E/NZ2/00598) — Dr Paweł P Łabaj
**Abstract:**	Microbiome-based disease prediction has significant potential as an early, non-invasive marker of multiple health conditions attributable to dysbiosis of the human gut microbiota, thanks in part to decreasing sequencing and analysis costs. Microbiome health indexes and other computational tools currently proposed in the field often rely and are based on a microbiome's species richness and are completely reliant on taxonomic classification. More recently, a resurgent interest in a metabolism-centric, ecological approach has led to an increased understanding of microbiome metabolic and phenotypic complexity revealing substantial restrictions of taxonomy-reliant approaches. In this study, we introduce a new metagenomic health index developed as an answer to the developments in microbiome definitions in an effort to distinguish healthy from unhealthy microbiomes, here inflammatory bowel disease (IBD) affliction based on two well-explored cohorts: the Human Microbiome Project 2 and American Gut Project. The novelty of our approach is a shift from a traditional Linnean phylogenetic classification towards a more holistic consideration of the metabolic functional potential underlining ecological interactions between species. We compare our method to the most comprehensive indices to date, the taxonomy-based Gut Microbiome Health Index (GMHI), and the high dimensional principal component analysis (hiPCA) methods, as well as to the standard taxon-, and function-based Shannon entropy scoring. We demonstrate a significant improvement in comparison to these methods. Furthermore, we validate our index's ability to distinguish between healthy and disease states using a variety of complementary benchmarking approaches. Finally, we demonstrate its superiority over the GMHI and the hiPCA on a set of diseases that we did not target, but which the two other methods had originally been developed on. Overall, we emphasize the potential of this metagenomic approach and advocate a shift towards functional approaches in order to better understand and assess microbiome health as well as provide directions for future index enhancements. Our method, q2-predict-dysbiosis (Q2PD), is freely available (https://github.com/Kizielins/q2-predict-dysbiosis).
**Corresponding Author:**	Paweł P Łabaj, PhD, DSc<br>Jagiellonian University in Krakow: Uniwersytet Jagiellonski w Krakowie<br>Krakow, POLAND
**Corresponding Author Secondary Information:**	
**Corresponding Author's Institution:**	Jagiellonian University in Krakow: Uniwersytet Jagiellonski w Krakowie
**Corresponding Author's Secondary Institution:**	
**First Author:**	Kinga Zielinska
**First Author Secondary Information:**	
**Order of Authors:**	Kinga Zielinska
	Klas I. Udekwu
	Witold Rudnicki
	Alina Frolova
	Paweł P Łabaj
**Order of Authors Secondary Information:**	

Powered by Editorial Manager® and ProduXion Manager® from Aries Systems Corporation

Additional Information:	
**Question**	**Response**
Are you submitting this manuscript to a special series or article collection?	Yes
Please select an option from the menu: as follow-up to "Are you submitting this manuscript to a special series or article collection?"	Functional Metagenomics
**Experimental design and statistics**<br><br>Full details of the experimental design and statistical methods used should be given in the Methods section, as detailed in our Minimum Standards Reporting Checklist. Information essential to interpreting the data presented should be made available in the figure legends.<br><br>Have you included all the information requested in your manuscript?	Yes
**Resources**<br><br>A description of all resources used, including antibodies, cell lines, animals and software tools, with enough information to allow them to be uniquely identified, should be included in the Methods section. Authors are strongly encouraged to cite Research Resource Identifiers (RRIDs) for antibodies, model organisms and tools, where possible.<br><br>Have you included the information requested as detailed in our Minimum Standards Reporting Checklist?	Yes
**Availability of data and materials**<br><br>All datasets and code on which the conclusions of the paper rely must be either included in your submission or deposited in publicly available repositories (where available and ethically appropriate), referencing such data using	Yes

Powered by Editorial Manager® and ProduXion Manager® from Aries Systems Corporation

a unique identifier in the references and in the “Availability of Data and Materials” section of your manuscript.<br><br>Have you have met the above requirement as detailed in our Minimum Standards Reporting Checklist?	

Click here to access/download;Manuscript;Manuscript_main_text.docx

Healthy microbiome - moving towards functional interpretation

Kinga Zielińska[1], Klas I. Udekwu[2,3],Witold Rudnicki[4,5], Alina Frolova[6,7], Paweł P Łabaj[1]

1 - Małopolska Centre of Biotechnology, Jagiellonian University, Krakow, Poland

2 - Department of Biological Sciences, Bioinformatics and Computational Biology Program, University of Idaho, Moscow, ID 83843, U.S.A.

3 - Swedish Environmental Epidemiology Centre, Department of Aquatic Sciences and Assessment, Swedish University of Agricultural Sciences, Uppsala, SE75007, Sweden

4 – Faculty of Computer Science, University of Białystok, Białystok, Poland

5 - Interdisciplinary Centre for Mathematical and Computational Modelling, University of Warsaw, Warsaw, Poland

6 - Institute of Molecular Biology and Genetics of National Academy of Sciences of Ukraine, Kyiv, Ukraine

7 - Kyiv Academic University, Kyiv, Ukraine

Abstract

Microbiome-based disease prediction has significant potential as an early, non-invasive marker of multiple health conditions attributable to dysbiosis of the human gut microbiota, thanks in part to decreasing sequencing and analysis costs. Microbiome health indexes and other computational tools currently proposed in the field often rely and are based on a microbiome's species richness and are completely reliant on taxonomic classification. More recently, a resurgent interest in a metabolism-centric, ecological approach has led to an increased understanding of microbiome metabolic and phenotypic complexity revealing substantial restrictions of taxonomy-reliant approaches. In this study, we introduce a new metagenomic health index developed as an answer to the developments in microbiome definitions in an effort to distinguish healthy from unhealthy microbiomes, here inflammatory bowel disease (IBD) affliction based on two well-explored cohorts: the Human Microbiome Project 2 and American Gut Project. The novelty of our approach is a shift from a traditional Linnean phylogenetic classification towards a more holistic consideration of the metabolic functional potential underlining ecological interactions between species. We compare our method to the most comprehensive indices to date, the taxonomy-based Gut Microbiome Health Index (`GMHI`), and the high dimensional principal component analysis (`hiPCA)` methods, as well as to the standard taxon-, and function-based Shannon entropy scoring. We demonstrate a significant improvement in comparison to these methods. Furthermore, we validate our index's ability to distinguish between healthy and disease states using a variety of complementary benchmarking approaches. Finally, we demonstrate its superiority over the `GMHI` and the `hiPCA` on a set of diseases that we did not target, but which the two other methods had originally been developed on. Overall, we emphasize the potential of this metagenomic approach and advocate a shift towards functional approaches in order to better understand and assess microbiome health as well as provide directions for future index

enhancements. Our method, **`q2-predict-dysbiosis (Q2PD)`**, is freely available (https://github.com/Kizielins/q2-predict-dysbiosis).

Introduction

The prevalence of a host of diseases and conditions peripherally or directly linked to microbiome health such as Inflammatory Bowel Disease (IBD), diabetes, obesity and even various cancers, continue to increase globally and substantial funds are currently spent on diagnosis and treatment (M'koma, 2013, Hong et al., 2019). While a correlation between gut microbiome composition and human health is widely acknowledged (Vijay and Valdes, 2022), the accurate identification of microbial and host markers of disease states remains elusive. Accordingly, the ability to evaluate patient health status based on a gut microbiome snapshot would be of high clinical value. Stool-based methods are promising because they can be collected non-invasively and frequently, accompanied by a short analysis time. Decreasing costs of stool-sample analysis via next generation sequencing makes microbiome-based characterization and diagnostics a strong competitor in the field (Bajaj et al., 2020).

Dysbiosis, defined as a perturbation of gut homeostasis, is believed to be accompanied by reduced microbiota diversity and increased prevalence of 'harmful' bacteria in adults (DeGruttola et al., 2016, Hrncir, 2022). It can be induced by a wide range of factors including infection, diet, exercise, antibiotics, stress or poor sleep (Martinez et al., 2021). The simplest interventions currently applied for the prevention or alleviation of mild microbiome dysbioses include dietary modification or prebiotics (often non-digestible food types which promote the growth of beneficial microorganisms), probiotics (live beneficial bacteria usually in the form of capsules) and lifestyle changes. More severe cases of gut dysbiosis failing to respond to the above interventions and may qualify for fecal microbiota transplants, FMTs, which are increasingly gaining traction in clinics worldwide (Bull and Plummer, 2015). However, host-microbiota and intra-microbiota interactions are both very complex

and highly individual and in spite of the successful application of FMT's, we have no real understanding of why or how they work as yet.

There are a number of accepted approaches for the evaluation of a gut microbiome health status based on stool composition. Alpha diversity (Shannon entropy, for example) is a frequent choice as microbiome richness was long believed to be a key driver of microbiome health and robustness (Li et al., 2022, Gong et al., 2016). Beta diversity has also been applied albeit to a lesser degree in a number of longitudinal studies, mainly identifying non-dysbiotic samples based on a time-resolved proximity to other healthy samples (Zouiouich et al., 2021). The most robust index to date, outperforming diversity indices, is the Gut Microbiome Health Index, or the **GMHI** (Gupta et al., 2020), recently renamed the Gut Microbiome Wellness Index (**GMWI**). It is based on the ratio of 50 microbial species associated with healthy or unhealthy gut ecosystems and is reported to exceed 73% accuracy in determining disease state, thus the authors suggesting that gut taxonomic signatures can predict health status. A follow-up version of the same method (**GMWI2)**, available as a pre-print is purely based on gut taxonomic signatures (Chang et al., 2023). Another metagenomic gut-health index expanding on the **GMHI** approach, **hiPCA** (Zhu et al., 2023), was introduced as a monitoring framework for personalized health purposes. The personalized approach is achieved by analyzing the contribution of each bacterium to the index, which allows for the identification of high-influence (ostensibly keystone) species in different patient groups. The **hiPCA** claim of better performance than the **GMHI** is attributed to the authors' application of additional transformation and clustering algorithms. Importantly, such studies are often defined by datasets limited in scope to industrialized nations and thus a less than complete consideration of diet-environment-microbiome interactions.

However, a recently re-visited definition of the microbiome emphasizes the importance of not just the microbiota (a community of microorganisms), but the whole "Theatre of Activity", ToA (Berg et al., 2020). This ToA includes structural elements (proteins, lipids, polysaccharides), metabolites and environmental conditions. It is tightly bound to its corresponding ecological niche, and the synergistic

Commented [fs1]: maybe all capitalized?

relations between species provide all the necessary, community defining components. Based on this definition, we maintain that an index constructed from taxonomy alone is hardly sufficient to accurately capture biological phenomena occurring within the gut environment – the key to understanding gut dysbiosis. Instead, we hypothesize that **to effectively determine health:** i) a metagenomic functional profile is required (microbiome phenotype); and, ii) species interactions (e.g measured as co-occurrence) but not just presence should be considered.

We introduce an approach that is based on identifiable metagenomic features of ecosystems beyond diversity measures and basic taxonomic information. This **function-**centric approach is broached in two ways: i) directly by evaluating the functional potential within and between species and ii) indirectly by assessing co-occurrence and synergism between bacterial species. Our goal is not only to distinguish between healthy and diseased, but also to quantify the degree of dysbiosis in each sample for the given cohort. We derive the health-describing features based on an exploratory analysis of healthy samples from the Human Microbiome Project 2 (HMP2, Lloyd-Price et al., 2019) and outperform **`Shannon entropy,`** the **`GMHI`** and the **`hiPCA`** in healthy versus inflammatory bowel disease (IBD) and obese classification. The effectiveness of our index is validated when classifying two other IBD-oriented American Gut Project cohorts (AGP, McDonald et al, 2018), further referred to as AGP_1 and AGP_2. What is more, despite being developed for IBD cohorts, our method outperforms the **`GMHI`** and the **`hiPCA`** on an unseen set of diseases that the two were originally developed on. Furthermore, it is effective at identifying longitudinal microbiome changes in COVID-19 patients, which the **`GMHI`** and the **`hiPCA`** are unable to capture. Our method **`q2-predict-dysbiosis (Q2PD)`** is freely available (https://github.com/Kizielins/q2-predict-dysbiosis).

Results

High prevalence of “core functions” in health

In order to develop a way to assess the degree of dysbiosis in a microbiome sample, we must define a microbiome healthy state. We based our initial analysis (described in the Methods section) on 384 healthy individuals from the HMP2 project and searched for the most prevalent species, regardless of abundance (Figure 1a). We observed that 50% of species were present in less than 5% of samples, and hardly any species were shared by all individuals. On the other hand, the prevalence of functions within the healthy population had an opposite trend - 50% of functions were already represented by at least 40% of individuals (Figure 1b), a functional redundancy unaccounted for in the **GMHI** (or the **hiPCA** which is based on it). These results convinced us further about the unsuitability of basing an index purely on the presence of "core taxa" and encouraged a shift of focus towards more prevalent functions instead.

The addition of healthy samples from two AGP cohorts maintained the function distribution profile obtained purely with HMP2 samples (Figure 1c). In order to test whether the functions were universal or cohort-specific, we calculated the distributions separately for functions present in 1, 2 or all 3 cohorts. We found that all functions missing from at least 1 cohort were present in less than 10% of samples, which indicated the occurrence of high-prevalence functions in all three cohorts. Based on the increased frequency of functions present in over 80% of samples (dotted line in Figure 1c), we defined this set as "core functions" (refer to Supplementary Table 1 for full list). According to the MetaCyc classification, 73.5% of "core functions" were assigned as "Biosynthesis" pathways, followed by 18.8% "Degradation/Utilization/Assimilation" and 7.6% "Generation of Precursor Metabolites and Energy", a few of these additionally classified as "Superpathways". The classification aligned well with a previously reported high prevalence of carbohydrate and amino acid metabolism-related pathways, potentially forming the functional microbiome core (Zou et al., 2019). A detailed analysis of the core functions identified in our study, however, is out of the scope of this manuscript.

Shannon entropy calculated on species and functions allowed for a good distinction between healthy and unhealthy individuals from the HMP2 (Figure 2a top). However, the trends were unclear in the two AGP cohorts (Figure 2a middle and bottom), indicating the need for more complex methods, expanding beyond microbiome richness, to classify datasets without obvious separation. Diversity analyses revealed that the number of functions per sample remained similar, or even increased, during microbiome transitions from healthy state to dysbiosis in HMP2 (Supplementary Figure 1). While this cannot be measured using metagenomics data alone, this could hypothetically be due to occurring or increased expression of genes usually silenced in the state of eubiosis. However, this observation was not reproduced in the AGP cohorts. We tested whether "core functions" are maintained or replaced by other functions in IBD samples, investigating the presence of "core functions" in different groups. While the differences were not significant in most cases, we noted a visibly higher preponderance of "core functions" and a higher percentage of all "core functions" in healthy as compared to disease samples (Supplementary Figure 2). We carried out differential enrichment analysis using LEfSe (Segata et al., 2011), performed separately for each cohort, and identified 100 functions that were more abundant in healthy as compared to disease cohorts (Figure 2b, Supplementary Figure 2). Over 90% of functions enriched in healthy samples were "core functions", while they constituted less than 5% of functions enriched in the unhealthy class of AGP_2 and HMP2 (Figure 2c). The LEfSe analysis on AGP_1 data revealed only 10 significantly enriched functions (7 in health and 3 in disease), all of which were core. This indicated a more heterogeneous functional landscape within this cohort.

Commented [PŁ2]: how this is connected to next sentence?
we have everywhere "functions" and here we have "pathways".

Species interactions and function contributions in health

In accordance with past studies, we observed a decrease in the number of species in dysbiotic samples (Supplementary Figure 3, Mirsepasi-Lauridsen et al., 2018, Mosca et al., 2016). Having previously noted an increase in the number of functions (Supplementary Figure 1), we speculated that the

remaining species may contribute to core or new functions, forming new connections with one another. Due to the substantial number of initial connections to analyze (170 "core functions" and 1490 species present in at least two projects), we restricted the number of species to those most informative in the context of health- / disease-state separation. We chose Multi-Dimensional Feature Selection (MDFS) algorithm, as it was the only feature selection method accounting for inter-feature interactions that we were aware of at the time of manuscript submission. This approach reduced the number of species to 587 allowing us to eliminate noise and focus on the most important interactions (see Methods for details about the feature selection procedure).

We then used the SparCC algorithm, designed specifically for compositional data, to investigate correlations between the resulting species in health and disease (Friedman and Alm, 2012). We did not observe any trends in the number of correlations, or in the fraction of positive correlations per group, that would indicate differences between the two. However, we identified opposite relationships of some species in different groups (Figure 3a). A number of species we found to be positively correlated in health and generally considered beneficial *(e.g. Eubacterium rectale*, *Faecalibacterium prausnitzii,* and a number of *Bacteroides* species), and those relationships would be disrupted in dysbiotic groups. We observed that the prevalence of the pairs positively correlated in health was higher than in a number of disease-associated groups (Figure 3b). We therefore added the co-occurrence of such species as another feature of interest potentially aiding in the determination of microbiome health.

Based on our previous results, we hypothesized that the contributions of each species to functions would be relatively stable in the healthy state and less predictable in disease. To test this, we compared the contributions of MDFS-identified species to "core functions" in different groups (Supplementary Figure 4). We did not observe any differences between health and disease, despite a relatively tight clustering of the healthy groups. However, we found stronger results when exploring functional redundancy. While the average number of species per function and the average number of

functions per species did not produce visual separation of the healthy and diseased (Supplementary Figure 5), the latter appeared informative in the context of health and disease separation (described in the subsequent sections). This finding was congruent with our earlier suspicions of functional plasticity; modulation of function and thus altered connectivity in the interaction network, shifting towards less abundant, non-core functions upon perturbation of homeostasis. It also, once again, proved the difficulty of identifying dysbiosis based on one feature alone – and highlighted the advantage of describing a microbiome from a variety of perspectives in order to make overall classification feasible.

Testing `q2-predict-dysbiosis`, `GMHI` and `hiPCA` accuracy of prediction for healthy and IBD individuals

Our final set of potentially health-defining microbiome features included the following parameters: i) the fraction of "core functions" found, ii) the fraction of "core functions" among all functions, iii) the fraction of co-occurrent species pairs in healthy samples, and iv) the average number of function contributions per species (details of how each feature was calculated can be found in Methods). In addition, we also included two parameters derived from the GMHI method – the number of “good” and “bad” GMHI species identified in a sample, which would enable us to compare between approaches. We then fed these described parameters into a machine learning model (see Figure 4a for the experimental design flowchart).

We opted for a Random Forest model due to its intrinsic ability to identify informative features and performed 5-fold cross-validation. Collecting information on feature importance at every iteration of the cross-validation procedure model, we consistently identified the two **`GMHI`** parameters as the least important (Figure 5b). On the other hand, the parameters defined by us merely switched positions among the top 4 places depending on the feature being excluded. We identified species co-

occurrence and the fraction of core functions among all functions as the most important features and the importance of each was individually significantly greater than that of the **GMHI** features combined. To confirm this observation, we also applied the MDFS 1D and 2D (pure feature selection and the inter-feature interaction-aware mode), both of which identified all parameters as relevant (Supplementary Table 2). We therefore decided to include all features, including the weakest **GMHI** parameters, in our final model.

Commented [PŁ3]: important or relevant?

Commented [PŁ4]: didn't we want to test option without GMHI features?

Next, we compared the **Shannon entropy, GMHI** and **hiPCA** values to our predictions (**q2-predict-dysbiosis [Q2PD]**) for two IBD subtypes: ulcerative colitis (UC) and Crohn's disease (CD) from the HMP2 and the two AGP cohorts (Figure 5). The actual predictions were performed using the leave-one-out cross validation procedure in order to maximize the size of the training set. In this comparison, increasingly higher scores were indicative of quantitative improvement in health status. In the case of the **GMHI**, a binary interpretation is used where any score greater than 0 is considered healthy. All four methods effectively distinguished between healthy and diseased individuals from the HMP2 cohorts, but **Shannon entropy** and the **GMHI** failed to make a correct distinction between healthy, UC and Obese samples from the AGP_2. In addition, the **hiPCA** was unsuccessful at separating any of the AGP_1 cohorts and failed to distinguish between healthy and Obese samples in the AGP_2 data. Most importantly, **Q2PD** produced visually the highest scores for all healthy in comparison to unhealthy cohorts. Despite the fact that our method was originally designed for within-cohort analyses, samples with a score of at least 0.6 were generally healthy in all cohorts. **Q2PD** was also the only index to produce a statistically significant difference between Healthy and Obese in HMP2. In an effort to quantitatively evaluate the performance of the indices, we used a Mann-Whitney U test (health and disease expressed in binary form) to estimate the significance of association between indices and the study groups. The **Q2PD** produced the strongest separation of healthy and diseased in HMP2 and AGP_2 and the **GMHI** in AGP_1 (Supplementary Table 3).

As differential sample preparation and software versions are known to influence sequencing results, we tested the **Q2PD** on the precalculated **MetaPhlAn3** and **HUMAnN3** profiles, released with the original HMP2 publication. We chose the **GMHI** as our benchmark because the **hiPCA** and **Shannon entropy** produced worse results for the HMP2 individuals (Figure 5). Both methods distinguished dysbiotic and eubiotic (a term coined for healthy living individuals' microbiomes) samples from healthy and CD groups, even though they were less concordant for UC patients (Supplementary Figure 6). While **Q2PD** did not identify significant differences between eubiosis and dysbiosis in UC individuals, it positively correlated with 37 metabolites and GMHI with 3 in this group. Among the metabolites positively correlating only with our approach was the artificial sweetener acesulfame, recently shown to induce proinflammatory cytokines and metabolic syndrome in mice (Hanawa et al., 2021). Correlations between the indexes and metabolites were similar in healthy groups, as was clustering based on index value correlations with metabolites resulting in the clustering of eubiotic groups with both the **GMHI** and the **Q2PD** (Supplementary Figure 7). A complete list of correlations can be found in Supplementary Table 4.

Cross-Disease Success: going beyond IBD

While only healthy and IBD individuals had originally been included in the development and validation of the approach so far, we wondered about the applicability of the **Q2PD** as it was to other diseases. In order to test the performance of the **Q2PD** more extensively, we used a number of datasets as different from our previous cohorts as possible. These were datasets upon which the **GMHI** and the **hiPCA** had originally been developed and thus both methods should perform better than ours (Figure 6). Although the threshold of 0.6 as determinant of health by the **Q2PD** was not applicable to the new datasets, its ability to differentiate between inter-cohort groups was surprising. While the **Q2PD** and

GMHI could separate healthy samples from colon cancer (Figure 6a) and acute diarrhoea (Figure 6b) samples, the **Q2PD** produced a better separation for both (**GMHI** colon cancer *p* < 0.05, **GMHI** acute diarrhoea *p* < 0.05, **Q2PD** colon cancer *p* < 0.001, **Q2PD** acute diarrhoea *p* < 0.01). Colon cancer sample separation produced by the **hiPCA** was significant, though not clearly discernible (p < 0.01), and discernible but not statistically significant for the acute diarrhoea samples (p > 0.05). The **GMHI** performed better distinguishing between healthy and atherosclerotic cardiovascular disease, ACVD, individuals (Figure 6c, *p* < 0.0001). However, even here, it returned several false positives, negative scores for non-diseased individuals, for both ACVD and metabolic syndrome (Figure 6d) cohorts, accordingly failing to identify healthy samples (GMHI metabolic syndrome *p* > 0.05). The **hiPCA** performed best in distinguishing between healthy and ACVD individuals. Interestingly, both the **GMHI** and the **hiPCA** predicted lower scores for healthy individuals than those with metabolic syndrome (*p* < 0.001). While the results produced by the **Q2PD** for ACVD and metabolic syndrome cohorts were not significant (*p* > 0.05 for both), the trend was of the expected direction.

To quantify the importance of each health index in the context of health and disease separation, we trained a separate random forest for each dataset. The input matrix consisted of health scores, predicted by each index as shown in Figure 5. The models produced an average AUC score of 0.72 (AUC values and errors for each dataset can be found in Supplementary Table 5). The AUC score was highly variable across the datasets, however, reaching below 0.6 for ACVD and one COVID-19 dataset (described in the subsequent section), while exceeding 0.9 for HMP2 and acute diarrhoea. Scores below 0.7 indicated problematic datasets that were difficult to classify, while those with very good (> 0.8) and outstanding (> 0.9) AUC values strongly demonstrated the clinical utility of the health indices at least in the context of group separation.

To further compare our method objectively, we also used **Boruta**, an all-relevant feature selection method, to rank health indices according to their importance [1]. The **Q2PD** was identified as the most important in all datasets that were included in its training and validation, specifically AGP_1, AGP_2 and HMP2 (Table 1, Supplementary Figure 7). The same was true when all datasets were taken into

Commented [PŁ5]: no mention about COVID data set earlier ... it is introduced in next section

account, as the **Q2PD** achieved the smallest mean of the ranks (mean rank of 2.88, versus 3.00, 3.88, 4.00 and 4.50 for **Shannon entropy**, **Shannon entropy on functions**, **GMHI** and **hiPCA**, respectively). What is more, the **Q2PD** was identified as the most important in three cases (out of 8), in comparison to two cases for the **Shannon entropy** and one case for the **Shannon entropy on functions** (added for benchmarking reasons) and the **GMHI**. The **hiPCA** was never selected as the most important. The **Q2PD, Shannon entropy** and **Shannon entropy on functions** were not recognized as relevant in two cases, whereas **hiPCA** and the **GMHI** three times. Interestingly, **Shannon entropy** and **Shannon entropy on functions** appeared to be complementary in certain scenarios and would never be rejected at the same time. For example, when **Shannon entropy** was identified as the most important in the Covid_case_1_ICU, Covid_case_2_ICU or Acute_diarrhoea datasets, **Shannon entropy on functions** was rejected or fifth in the ranking. On the other hand, when **Shannon entropy on functions** would lead the classification in Colon_cancer, **Shannon entropy** was fourth. Overall, the ranks of most health indices were spread – each method would both lead (or be second) in one case, whereas it would be placed in the lowest rank in another. This was not the case for the **Q2PD**, however - which would be either in top 3 or be rejected along with other methods.

Commented [PŁ6]: ?? repetition? shouldn't be Covid_case_2_ICU?

Table 1. Boruta ranks for health indices in the context of disease prediction.

	**Shannon entropy**	**Shannon entropy on functions**	**GMHI**	**hiPCA**	**Q2PD**
**HMP2**	2	4	3	5	1
**AGP_1**	Rejected	3/3	3/3	Rejected	3/3
**AGP_2**	2	3	5	4	1
**Covid_case_1_ICU**	1	Rejected	Rejected	Rejected	Rejected
**Covid_case_2_ICU**	2	Rejected	Rejected	Rejected	1

**Colon_cancer**	4	1	Rejected	3	2
**Acute_diarrhoea**	1	5	2	4	3
**ACVD**	Rejected	3	1	2	Rejected
**Mean rank**	3.00	3.88	4.00	4.50	**2.88**
**Number of rejects**	2	2	3	3	2
**Number of wins**	2	1	1	0	**3**

Covid_case_noICU and metabolic syndrome not included as one feature was tentative, the rest was rejected. Rejected = rank 6. The rank of "3/3" in AGP_1 indicates that all three indices were marked as tentative, but included here as the AUC score provided by the random forest was significant.

Q2PD Robustness to longitudinal alterations and sequencing depth

To evaluate the ability of the three approaches to compare longitudinal and sparse microbiome data, we applied them to another type of data - a shallowly sequenced COVID-19 dataset. The set consisted of three groups - controls (healthy hospital staff), and COVID-19 patients who, during the course of the treatment, were either transferred to the intensive care unit (ICU) or recovered (noICU). For every individual, two timepoints were selected - "1" that was collected at hospital admission or early in the pandemic in the case of staff, and "2" which was the final sample taken from each individual. All methods determined similar health levels for controls and noICU patients, though only the **hiPCA** and the **Q2PD** indicated a noticeable, albeit non-significant decrease in health of the control staff as the pandemic progressed (Figure 7a). More importantly, while **Q2PD** determined a predictable decrease in the health of ICU-transferred patients, the **GMHI** and the **hiPCA** produced the opposite trend, wrongly indicating patient recovery. In addition, the **hiPCA** and the **GMHI** failed to identify

differences between the 2_ICU and both noICU groups, whereas the separation of the two cohorts was clearly evident from the **Q2PD** analysis. Recalculating scores on deeply sequenced paired samples confirmed the ability of the **Q2PD** to distinguish between healthy and ICU samples processed with different sequencing types (Figure 7b). **Q2PD** was the only index to produce statistically significant differences between the controls and both ICU timepoints of the expected direction. The **hiPCA** produced significantly lower scores for controls versus 1_ICU samples, while the **GMHI** produced the highest scores for the patients after ICU admission. The difference between samples collected upon admission and upon patient transfer to the ICU disappeared in the deeply sequenced samples which suggests that some hallmarks of the non-healthy status are of low abundance and thus require a sufficient sequencing depth.

The separation of healthy, 1_ICU and 2_ICU patients varied dependent upon sequencing method, so we explored the robustness of the **GMHI** and **Q2PD** to variation in sequencing depth. Because the performance of the **hiPCA** on the COVID-19 cohort was worse than that of the **GMHI**, we decided to treat the latter as our benchmark. In order to investigate method robustness to depth, we applied various degrees of rarefaction to deeply sequenced control samples expecting to see similar importance scores despite changing sequencing depth. The scores appeared to increase with increasing degree of rarefaction, and although negligible, this change occurred similarly for both methods (Figure 8 a, b). The results corresponding to the lowest coverage considered (2,000,000 reads reported a reasonable consensus between cost and resolution (Hillmann et al., 2018))) would be visibly separated from the remaining values, indicating this as a coverage threshold for method stability. Plotting values for each feature separately revealed that at this level certain species (Figure 8 c, d) or "core functions" (Figure 8 e, f) are undetectable. "The fraction of core features among other features" and "the mean number of function contributions per species" would vary most with coverage, which was anticipated considering their dependence on low-abundance functions (Figure 8 e - h). Interestingly, the numbers of "good" and "bad" species identified at any sequencing depth would cover only 32% of the complete **GMHI** list (2 and 14, versus 7 and 43 for good and bad species,

respectively). In line with the underlying theme of our work, this overlap argues against taxonomy-based indexes. Furthermore, the **GMHI** classification appears to be only relevant for the depth of 2 million reads (score greater than zero), as the healthy samples are defined as diseased at greater sequencing depth (scores below zero), as seen in Figure 8a. This is not the case for our index which correctly identifies health at any sequencing depth (score is always above the threshold selected to maximize accuracy for classification on shallow sequencing). Overall, we show the values from **Q2PD** to be surprisingly robust considering the variation of its components, being nearly as stable as the values of the **GMHI** across sequencing depths albeit only above a high sequencing depth (> 7,000,000 reads). For **Q2PD**, the decrease in value with an increased sequencing depth does not affect health status prediction while for the **GMHI**, even a small change in sequencing depth can result in erroneous health status predictions as evidenced by their values' proximity to author-defined threshold values for COVID-19 samples. Consideration for depth is required when comparing health between samples with significantly different coverage and milder dysbiotic symptoms.

Discussion

A connection between the human gut microbiome and gut health is now well established, and a number of approaches have been employed to try to identify gut dysbiosis from sequence-based analysis of stool samples. Those methods are based on taxonomy and rely either on measures of microbiome richness (alpha / beta diversity) or on the presence or absence of "good" and "bad" bacteria, proposing the health-indexes **GMHI** (Gupta et al., 2020) and **hiPCA** (Zhu et al., 2023). Hampering such approaches, however, are ecological considerations of metabolic or functional redundancies inherent within complex environments. Currently, re-defining the microbiome to

include inferred functionality is bolstered by recent studies that highlight the importance of interactions between microbiome components and functional aspects thereof To address the inadequacy of this *Linnéan* approach to evaluating change in microbiomes, we developed a novel method that incorporates function in bioinformatics-based assessment of microbiome dysbioses. We show that features based on microbiome functions and interactions define a healthy microbiome more accurately. There exists a set of "core functions", which are consistently identified as present in healthy gut microbiomes, and which disappear in the advent of dysbiosis. We compare our results to the **hiPCA** and the **GMHI**, (rather than to the newer **GMWI2)**, to benchmark our work against a version that is extensively validated by a wide range of users. By applying feature selection, we demonstrate that all parameters defined by us are relevant for healthy versus diseased state classification. The **Q2PD** outperforms **Shannon entropy,** the **GMHI** and the **hiPCA** in the separation of healthy and IBD / obese individuals from the HMP2 and two AGP cohorts. In addition, the **Q2PD** shows superior performance to the **GMHI** and the **hiPCA** based on a range of datasets with diseases **Q2PD** had not previously encountered and significantly, that the **GMHI** and the **hiPCA** had originally been trained with and developed on.

While we extol the performance **Q2PD**, we acknowledge the challenges that remain to be addressed in subsequent development of the method in order to make it more robust for diseases other than IBD and obesity, and eventual clinic deployment. Clearly, a deeper understanding of how the identified function- and interaction-based microbiome features respond to variations in sequencing depth and quality is required. We find that longitudinal data can provide more insight into personalized "core features" of a microbiome, and hence indicate individual deviations from the normal. This finding points to guidelines for index rescaling and cross-cohort unification, ultimately allowing for analysis of datasets from different studies (meta-analysis). Notably, we also find that individuals with different diseases and cohorts can be separated by different parameters. Thus, tracking microbiome changes in individuals over time may eventually allow for the identification of microbiome trajectories or which alterations that can occur, and better patient classification. Finally, the specific features describing an

individual’s microbiome changes could help determine risk for certain conditions or diseases, and a personalized clinical intervention.

A caveat to this type of study is the infancy of certain fields in particular, the availability of functional annotation software. Currently, the only widely-accepted functional annotation software is HUMAnN (any version), which requires data to be processed in a particular way. In future work we plan to explore other possibilities and ultimately migrate towards a more universal solution, such as gene content. Only then can recent developments in augmented functional annotation be efficiently utilized, as we already have shown in other applications (Maranga et al., 2023, Koehler Leman et al., 2023, Szydlowski et al., 2023).

Commented [PŁ7]: ... particular .... particular, ...
maybe:
... is the infancy of certain fields in particular, ...

We also consider expanding to a multi-omics approach in our predictions. With decreasing costs of metagenomics, metabolomics and proteomics data acquisition, predicting patient health status by the incorporation of all three -omics methods might be a reasonable option that carries significant improvements in accuracy and prediction in the not-too-distant future. It would also allow us to quantitatively estimate the functions directly, instead of basing our analyses on the functional potential evaluated based on metagenomics data.

In conclusion, we highlight the performance and high accuracy obtained by **Q2PD**, based only on a limited number of parameters. Despite its being developed originally to separate healthy and IBD individuals from the HMP2 and AGP cohorts, it proved to accurately distinguish between healthy patients and individuals with several other conditions. Motivated by this success we are positive that our methodology and approach are accurately oriented towards a better understanding of the human gut microbiome and its health, based on fundamental resource and interaction mediated ecological principles that ultimately define microbial community structure.

Methods

Metagenomic Data Processing

The overview of datasets used in this study can be found in Supplementary Table 6. All raw shotgun sequencing fastq files were processed with **`Trim Galore 0.6.10`** (Krueger, 2023) to ensure sufficient read quality. The taxonomic profiles were calculated with **`MetaPhlAn 4.0.6`** (Blanco-Míguez et al., 2023) and functional profiles with **`HUMAnN 3.7`** (Beghini et al., 2021). Species selection was performed using MultiDimentional Feature Selection, or the **`MDFS`** (Piliszek et al., 2018, version 1.5.2), with a Benjamini-Hochberg p-value correction in a 2D mode. Selection was performed per cohort (healthy versus each of the non-healthy groups in separate **`MDFS`** runs), and the final list of species was the union of the MDFS results for all cohorts (corrected p-values < 0.05). Rarefaction of deep COVID-19 samples, originally published by us before (Kopera et al., 2023), was performed with **`seqtk 1.4`** (Li, 2023).

Random Forest training

Random Forest is a classification and regression method. The algorithm utilises an ensemble of CART trees [2], in which each tree is built using different bootstrap samples of data and different random subsets of variables at each stage of the tree construction. It is a robust and versatile algorithm that works well on different types of data (Fernandez-Delgado et al., 2014).

Metagenomic features passed to the random forest were constructed based on healthy samples from the HMP2 and two AGP cohorts (AGP_1 and AGP_2). The features were as follows:

- the fraction of "core functions" found (number of “core functions” in a sample / number of all “core functions” in our list)

- the fraction of "core functions" among all functions (number of “core functions” in a sample / number of all functions in a sample)
- the fraction of species pairs commonly occurring together in healthy samples (SparCC correlation of species > 0.1, only nonnegative correlations in healthy cohorts)
- the average number of function contributions per species (number of all species to functions contributions based on stratified output / number of all species in a sample)
- the numbers of “good” and “bad” GMHI species.

The importance of the features was assessed using a permutation approach and 5-fold cross validation.

During random forest training, healthy individuals were labeled as “1” and the remaining IBD + obese individuals from the same projects were added as disease samples and labeled as “0”. A leave-one-out cross-validation (613 iterations) procedure was applied to predict health scores for each sample in order to avoid model overfitting or the need to split the data into training and testing sets. The final health score is the output of the random forest’s *predict_proba* method, which expresses the probability of each sample belonging to the healthy (or unhealthy) group.

The model was originally developed for, trained and validated on healthy versus IBD / Overweight cohorts from the HMP2 and AGP. It was then tested on cohorts with other diseases.

Index validation

To compare the four health indices we used two machine learning algorithms. A random forest was chosen to measure the amount of biological information captured by those indices, and Boruta [1] for an all-relevant feature selection to identify truly important indices.

The Boruta algorithm is a wrapper around the Random Forest classifier. It works in the following way: a data set is extended by adding a randomly permuted copy of each original variable - a so-called

shadow variable. A predictive model for the decision variable is then built using the random forest algorithm. The importance of each variable is estimated using a permutation test. Boruta collects the information about the importance and then compares the importance score of each original variable with the maximal importance achieved by a shadow variable.

The procedure is repeated multiple times, and a statistical test is performed. The variables are eventually assigned to three classes: Confirmed (better than random), Rejected (no better than random) and Tentative (those that could not be assigned neither to Confirmed no to the Tentative class. The utility of the variable is a good indicator of the information importance carried by the variable, also in situations where the synergistic interactions are important.

Downstream analyses

Metagenomic table formatting and alpha diversity plots were done using **QIIME 2** (Bolyen et al., 2019). The GMHI scores were calculated with the **QIIME 2 q2-health-index** plugin ("q2-health-index," 2023) and **SparCC** correlations with the **SCNIC** plugin (https://github.com/lozuponelab/q2-SCNIC). The **hiPCA** (Zhu et al., 2023) predictions were obtained by substituting the original test file with our test samples, due to a lack of instructions about how to do it better (this could potentially result in an overlap of the train and test samples, possibly falsely increasing the **hiPCA**'s accuracy). Feature importance was calculated using **MDFS** and **Boruta** [1]. Plots were made using custom python scripts. Any statistical tests were calculated with the independent t-test, unless stated otherwise.

Data and code availability

The HMP2 data can be found in SRA with the accession code PRJNA398089. The two AGP projects can be located under accessions PRJNA389280 and PRJEB1220. The four cohorts from the curatedMetagenomicsData were WirbelJ_2018 (colon cancer, ENA: PRJEB27928), DavidLA_2015 (acute diarrhoea, PRJEB9150), JieZ_2017 (ACVD, ERP023788) and LiSS_2016 (metabolic syndrome, PRJEB12357). The COVID-19 cohort can be found under the accession PRJEB64515 in the European Nucleotide Archive. Our method is deposited on GitHub and can be accessed here: https://github.com/bioinf-mcb/q2-predict-dysbiosis.

Acknowledgements

This research was conducted as a part of the NCN Sonata BIS grant number 2020/38/E/NZ2/00598. We gratefully acknowledge Poland's high-performance Infrastructure PLGrid (HPC Centers: ACK Cyfronet AGH, PCSS, CI TASK, WCSS) for providing computer facilities. We would like to thank Dr Krzysztof Mnich, Dr Sajad Shahbazi, Dr Balakrishnan Subramanian and Piotr Stomma from the University of Białystok for their expertise in the Multi-Dimensional Feature Selection algorithm and insightful contributions to our work, dr. Tomasz Kościółek from the Sano Centre for Computational Medicine and the members of Bioinformatics Research Group at MCB JU for their comments and support.

Ethics declarations

All authors declare no competing interests.

Bibliography

American Gut: an Open Platform for Citizen Science Microbiome Research | mSystems [WWW Document], n.d. URL https://journals.asm.org/doi/10.1128/msystems.00031-18 (accessed 12.3.23).

Bajaj, J.S., Acharya, C., Sikaroodi, M., Gillevet, P.M., Thacker, L.R., 2020. Cost-effectiveness of integrating gut microbiota analysis into hospitalisation prediction in cirrhosis. GastroHep 2, 79–86. https://doi.org/10.1002/ygh2.390

Beghini, F., McIver, L.J., Blanco-Míguez, A., Dubois, L., Asnicar, F., Maharjan, S., Mailyan, A., Manghi, P., Scholz, M., Thomas, A.M., Valles-Colomer, M., Weingart, G., Zhang, Y., Zolfo, M., Huttenhower, C., Franzosa, E.A., Segata, N., 2021. Integrating taxonomic, functional, and strain-level profiling of diverse microbial communities with bioBakery 3. eLife 10, e65088. https://doi.org/10.7554/eLife.65088

Berg, G., Rybakova, D., Fischer, D., Cernava, T., Vergès, M.-C.C., Charles, T., Chen, X., Cocolin, L., Eversole, K., Corral, G.H., Kazou, M., Kinkel, L., Lange, L., Lima, N., Loy, A., Macklin, J.A., Maguin, E., Mauchline, T., McClure, R., Mitter, B., Ryan, M., Sarand, I., Smidt, H., Schelkle, B., Roume, H., Kiran, G.S., Selvin, J., Souza, R.S.C. de, van Overbeek, L., Singh, B.K., Wagner, M., Walsh, A., Sessitsch, A., Schloter, M., 2020. Microbiome definition re-visited: old concepts and new challenges. Microbiome 8, 103. https://doi.org/10.1186/s40168-020-00875-0

Blanco-Míguez, A., Beghini, F., Cumbo, F., McIver, L.J., Thompson, K.N., Zolfo, M., Manghi, P., Dubois, L., Huang, K.D., Thomas, A.M., Nickols, W.A., Piccinno, G., Piperni, E., Punčochář, M., Valles-Colomer, M., Tett, A., Giordano, F., Davies, R., Wolf, J., Berry, S.E., Spector, T.D., Franzosa, E.A., Pasolli, E., Asnicar, F., Huttenhower, C., Segata, N., 2023. Extending and improving metagenomic taxonomic profiling with uncharacterized species using MetaPhlAn 4. Nat. Biotechnol. 41, 1633–1644. https://doi.org/10.1038/s41587-023-01688-w

Bolyen, E., Rideout, J.R., Dillon, M.R., Bokulich, N.A., Abnet, C.C., Al-Ghalith, G.A., Alexander, H., Alm,

E.J., Arumugam, M., Asnicar, F., Bai, Y., Bisanz, J.E., Bittinger, K., Brejnrod, A., Brislawn, C.J., Brown, C.T., Callahan, B.J., Caraballo-Rodríguez, A.M., Chase, J., Cope, E.K., Da Silva, R., Diener, C., Dorrestein, P.C., Douglas, G.M., Durall, D.M., Duvallet, C., Edwardson, C.F., Ernst, M., Estaki, M., Fouquier, J., Gauglitz, J.M., Gibbons, S.M., Gibson, D.L., Gonzalez, A., Gorlick, K., Guo, J., Hillmann, B., Holmes, S., Holste, H., Huttenhower, C., Huttley, G.A., Janssen, S., Jarmusch, A.K., Jiang, L., Kaehler, B.D., Kang, K.B., Keefe, C.R., Keim, P., Kelley, S.T., Knights, D., Koester, I., Kosciolek, T., Kreps, J., Langille, M.G.I., Lee, J., Ley, R., Liu, Y.-X., Loftfield, E., Lozupone, C., Maher, M., Marotz, C., Martin, B.D., McDonald, D., McIver, L.J., Melnik, A.V., Metcalf, J.L., Morgan, S.C., Morton, J.T., Naimey, A.T., Navas-Molina, J.A., Nothias, L.F., Orchanian, S.B., Pearson, T., Peoples, S.L., Petras, D., Preuss, M.L., Pruesse, E., Rasmussen, L.B., Rivers, A., Robeson, M.S., Rosenthal, P., Segata, N., Shaffer, M., Shiffer, A., Sinha, R., Song, S.J., Spear, J.R., Swafford, A.D., Thompson, L.R., Torres, P.J., Trinh, P., Tripathi, A., Turnbaugh, P.J., Ul-Hasan, S., vander Hooft, J.J.J., Vargas, F., Vázquez-Baeza, Y., Vogtmann, E., von Hippel, M., Walters, W., Wan, Y., Wang, M., Warren, J., Weber, K.C., Williamson, C.H.D., Willis, A.D., Xu, Z.Z., Zaneveld, J.R., Zhang, Y., Zhu, Q., Knight, R., Caporaso, J.G., 2019. Reproducible, interactive, scalable and extensible microbiome data science using QIIME 2. Nat. Biotechnol. 37, 852–857. https://doi.org/10.1038/s41587-019-0209-9

Breiman, L., 2001. Random Forests. Mach. Learn. 45, 5–32. https://doi.org/10.1023/A:1010933404324

Breiman, L., Friedman, J., Olshen, R.A., Stone, C.J., 2017. Classification and Regression Trees. Chapman and Hall/CRC, New York. https://doi.org/10.1201/9781315139470

Bull, M.J., Plummer, N.T., 2015. Part 2: Treatments for Chronic Gastrointestinal Disease and Gut Dysbiosis. Integr. Med. Clin. J. 14, 25–33.

DeGruttola, A.K., Low, D., Mizoguchi, A., Mizoguchi, E., 2016. Current understanding of dysbiosis in disease in human and animal models. Inflamm. Bowel Dis. 22, 1137–1150. https://doi.org/10.1097/MIB.0000000000000750

Friedman, J., Alm, E.J., 2012. Inferring Correlation Networks from Genomic Survey Data. PLOS Comput. Biol. 8, e1002687. https://doi.org/10.1371/journal.pcbi.1002687

Gong, D., Gong, X., Wang, L., Yu, X., Dong, Q., 2016. Involvement of Reduced Microbial Diversity in Inflammatory Bowel Disease. Gastroenterol. Res. Pract. 2016, 6951091. https://doi.org/10.1155/2016/6951091

Gupta, V.K., Kim, M., Bakshi, U., Cunningham, K.Y., Davis, J.M., Lazaridis, K.N., Nelson, H., Chia, N., Sung, J., 2020. A predictive index for health status using species-level gut microbiome profiling. Nat. Commun. 11, 4635. https://doi.org/10.1038/s41467-020-18476-8

Gut Microbiome Wellness Index 2 for Enhanced Health Status Prediction from Gut Microbiome Taxonomic Profiles | bioRxiv [WWW Document], n.d. URL https://www.biorxiv.org/content/10.1101/2023.09.30.560294v1 (accessed 12.3.23).

Hanawa, Y., Higashiyama, M., Kurihara, C., Tanemoto, R., Ito, S., Mizoguchi, A., Nishii, S., Wada, A., Inaba, K., Sugihara, N., Horiuchi, K., Okada, Y., Narimatsu, K., Komoto, S., Tomita, K., Hokari, R., 2021. Acesulfame potassium induces dysbiosis and intestinal injury with enhanced lymphocyte migration to intestinal mucosa. J. Gastroenterol. Hepatol. 36, 3140–3148. https://doi.org/10.1111/jgh.15654

Hillmann, B., Al-Ghalith, G.A., Shields-Cutler, R.R., Zhu, Q., Gohl, D.M., Beckman, K.B., Knight, R., Knights, D., 2018. Evaluating the Information Content of Shallow Shotgun Metagenomics. mSystems 3, 10.1128/msystems.00069-18. https://doi.org/10.1128/msystems.00069-18

Hong, Y.-R., Huo, J., Desai, R., Cardel, M., Deshmukh, A.A., 2019. Excess Costs and Economic Burden of Obesity-Related Cancers in the United States. Value Health J. Int. Soc. Pharmacoeconomics Outcomes Res. 22, 1378–1386. https://doi.org/10.1016/j.jval.2019.07.004

Hrncir, T., 2022. Gut Microbiota Dysbiosis: Triggers, Consequences, Diagnostic and Therapeutic Options. Microorganisms 10, 578. https://doi.org/10.3390/microorganisms10030578

Koehler Leman, J., Szczerbiak, P., Renfrew, P.D., Gligorijevic, V., Berenberg, D., Vatanen, T., Taylor,

B.C., Chandler, C., Janssen, S., Pataki, A., Carriero, N., Fisk, I., Xavier, R.J., Knight, R., Bonneau, R., Kosciolek, T., 2023. Sequence-structure-function relationships in the microbial protein universe. Nat. Commun. 14, 2351. https://doi.org/10.1038/s41467-023-37896-w

Kopera, K., Gromowski, T., Wydmański, W., Skonieczna-Żydecka, K., Muszyńska, A., Zielińska, K., Wierzbicka-Woś, A., Kaczmarczyk, M., Kadaj-Lipka, R., Cembrowska-Lech, D., Januszkiewicz, K., Kotfis, K., Witkiewicz, W., Nalewajska, M., Feret, W., Marlicz, W., Łoniewski, I., Łabaj, P.P., Rydzewska, G., Kosciolek, T., 2023. Gut Microbiome Dynamics and Predictive Value in Hospitalized COVID-19 Patients: A Comparative Analysis of Shallow and Deep Shotgun Sequencing. https://doi.org/10.1101/2023.11.29.568526

Krueger, F., 2023. Trim Galore.[1] M. B. Kursa and W. R. Rudnicki, 'Feature Selection with the Boruta Package', *J. Stat. Softw.*, vol. 36, pp. 1–13, Sep. 2010, doi: 10.18637/jss.v036.i11.

[2] J. F. Leo Breiman, *Classification and Regression Trees*. Chapman and Hall/CRC, 1984.

[3] M. Fernandez-Delgado, E. Cernadas, S. Barro, and D. Amorim, 'Do we Need Hundreds of Classifiers to Solve Real World Classification Problems?'.

Li, H., 2023. lh3/seqtk.

Li, Z., Zhou, J., Liang, H., Ye, L., Lan, L., Lu, F., Wang, Q., Lei, T., Yang, X., Cui, P., Huang, J., 2022. Differences in Alpha Diversity of Gut Microbiota in Neurological Diseases. Front. Neurosci. 16.

Lloyd-Price, J., Arze, C., Ananthakrishnan, A.N., Schirmer, M., Avila-Pacheco, J., Poon, T.W., Andrews, E., Ajami, N.J., Bonham, K.S., Brislawn, C.J., Casero, D., Courtney, H., Gonzalez, A., Graeber, T.G., Hall, A.B., Lake, K., Landers, C.J., Mallick, H., Plichta, D.R., Prasad, M., Rahnavard, G., Sauk, J., Shungin, D., Vázquez-Baeza, Y., White, R.A., Braun, J., Denson, L.A., Jansson, J.K., Knight, R., Kugathasan, S., McGovern, D.P.B., Petrosino, J.F., Stappenbeck, T.S., Winter, H.S., Clish, C.B., Franzosa, E.A., Vlamakis, H., Xavier, R.J., Huttenhower, C., 2019. Multi-omics of the gut microbial ecosystem in inflammatory bowel diseases. Nature 569, 655–662. https://doi.org/10.1038/s41586-019-1237-9

Maranga, M., Szczerbiak, P., Bezshapkin, V., Gligorijevic, V., Chandler, C., Bonneau, R., Xavier, R.J., Vatanen, T., Kosciolek, T., 2023. Comprehensive Functional Annotation of Metagenomes and

Microbial Genomes Using a Deep Learning-Based Method. mSystems 8, e01178-22. https://doi.org/10.1128/msystems.01178-22

Martinez, J.E., Kahana, D.D., Ghuman, S., Wilson, H.P., Wilson, J., Kim, S.C.J., Lagishetty, V., Jacobs, J.P., Sinha-Hikim, A.P., Friedman, T.C., 2021. Unhealthy Lifestyle and Gut Dysbiosis: A Better Understanding of the Effects of Poor Diet and Nicotine on the Intestinal Microbiome. Front. Endocrinol. 12.

Mirsepasi-Lauridsen, H.C., Vrankx, K., Engberg, J., Friis-Møller, A., Brynskov, J., Nordgaard-Lassen, I., Petersen, A.M., Krogfelt, K.A., 2018. Disease-Specific Enteric Microbiome Dysbiosis in Inflammatory Bowel Disease. Front. Med. 5.

M'koma, A.E., 2013. Inflammatory Bowel Disease: An Expanding Global Health Problem. Clin. Med. Insights Gastroenterol. 6, CGast.S12731. https://doi.org/10.4137/CGast.S12731

Mosca, A., Leclerc, M., Hugot, J.P., 2016. Gut Microbiota Diversity and Human Diseases: Should We Reintroduce Key Predators in Our Ecosystem? Front. Microbiol. 7.

Piliszek, R., Mnich, K., Migacz, S., Tabaszewski, P., Sułecki, A., Polewko-Klim, A., Rudnicki, W., 2018. MDFS - MultiDimensional Feature Selection.

Segata, N., Izard, J., Waldron, L., Gevers, D., Miropolsky, L., Garrett, W.S., Huttenhower, C., 2011. Metagenomic biomarker discovery and explanation. Genome Biol. 12, R60. https://doi.org/10.1186/gb-2011-12-6-r60

Szydlowski, L.M., Bulbul, A.A., Simpson, A.C., Kaya, D.E., Singh, N.K., Sezerman, U.O., Łabaj, P.P., Kosciolek, T., Venkateswaran, K.J., 2023. Adaptation to space conditions of novel bacterial species isolated from the International Space Station revealed by functional gene annotations and comparative genome analysis. https://doi.org/10.1101/2023.09.28.559980

Vijay, A., Valdes, A.M., 2022. Role of the gut microbiome in chronic diseases: a narrative review. Eur. J. Clin. Nutr. 76, 489–501. https://doi.org/10.1038/s41430-021-00991-6

Zhu, J., Xie, H., Yang, Z., Chen, J., Yin, J., Tian, P., Wang, H., Zhao, J., Zhang, H., Lu, W., Chen, W., 2023. Statistical modeling of gut microbiota for personalized health status monitoring.

Microbiome 11, 184. https://doi.org/10.1186/s40168-023-01614-x

Zou, Y., Xue, W., Luo, G., Deng, Z., Qin, P., Guo, R., Sun, H., Xia, Y., Liang, S., Dai, Y., Wan, D., Jiang, R., Su, L., Feng, Q., Jie, Z., Guo, T., Xia, Z., Liu, C., Yu, J., Lin, Y., Tang, S., Huo, G., Xu, X., Hou, Y., Liu, X., Wang, J., Yang, H., Kristiansen, K., Li, J., Jia, H., Xiao, L., 2019. 1,520 reference genomes from cultivated human gut bacteria enable functional microbiome analyses. Nat. Biotechnol. 37, 179–185. https://doi.org/10.1038/s41587-018-0008-8

Zouiouich, S., Loftfield, E., Huybrechts, I., Viallon, V., Louca, P., Vogtmann, E., Wells, P.M., Steves, C.J., Herzig, K.-H., Menni, C., Jarvelin, M.-R., Sinha, R., Gunter, M.J., 2021. Markers of metabolic health and gut microbiome diversity: findings from two population-based cohort studies. Diabetologia 64, 1749–1759. https://doi.org/10.1007/s00125-021-05464-w

Figures

Figure 1. Distributions of species **(a)** and functions **(b)** present in healthy individuals from the HMP2; absolute values of species and function counts are shown as histograms (with scales on the right-hand side, with shaded cumulative sum in the background and an inverse of the cumulative sum represented with a dashed line (with scales on the left-hand side). **(c)** Distribution of functions in healthy individuals from the HMP2, AGP_1 and AGP_2.

Figure 2. (**a**) Shannon entropy scores for species and functions in healthy and unhealthy individuals from the HMP2, AGP_1 and AGP_2. Fraction of "core functions" found per sample. (**b**) LEfSe differential enrichment analysis: overlap of enriched pathways in healthy and unhealthy individuals in the AGP_1, AGP_2 and HMP2 projects. (**c**) Fraction of core functions among differentially enriched functions in healthy and unhealthy individuals in the AGP_1, AGP_2 and HMP2 projects.

Commented [PŁ8]: Amount of seen core functions among all annotated core functions.

Figure 3. (**a**) SparCC correlations between species, restricted to pairs non-negatively correlated in health. (**b**) Prevalence of the pairs in different cohorts.

Figure 4. (a) Schematic representation of the Q2PD. **(b)** Feature importance for the Q2PD, defined by the random forest “feature_importances” method over 5 rounds of cross-validation.

Figure 5. Shannon entropy, GMHI, hiPCA and Q2PD scores for HMP2 and AGP healthy, IBD and Obese individuals. Red stars indicate statistically significant differences between groups.

Commented [fs9]: From this plot alone I can conclude that GMHI is better, because it gives 5 significant comparisons (5 *), while q2 is only 4. Yes, the text above says that q2 gave stronger difference, but some people might argue it doesn't matter as long as there is significant different. Are you sure there is no significance in AGP_1 for q2? Based on the plot it looks like there should be (cause healthy is so different from the rest).

Commented [KZ10R9]: There is no statistically significant diffference because the means are very close. I added more comparisons in the supplement and in Table 1 specifically for that to show different performance aspects.

Figure 6. Comparison of the GMHI, the hiPCA and the Q2PD predictions for colon cancer (**a**), acute diarrhoea (**b**), artherosclerotic cardiovascular disease (**c**) and metabolic syndrome (**d**).

Figure 7. Comparison of the GMHI, the hiPCA and Q2PD predictions on COVID-19 samples processed using shallow (**a**) and deep (**b**) sequencing. Q2PD was the only method to produce a significant difference between healthy and COVID-19 patients.

Figure 8. Robustness of health predictions to sequencing coverage. GMHI (**a**) and Q2PD (**b**) scores for deeply sequenced healthy samples from the COVID-19 cohort, rarefied to corresponding depths. The horizontal line in (a) represents a health threshold. Horizontal lines in (b) represent health thresholds for shallow (dashed) and deep (full) sequencing, defined specifically for the COVID-19 cohort based on Figure 7 – maximizing classification accuracy. Taxonomic GMHI-inspired Q2PD features (**c**, **d**)

remain relatively stable above the coverage of 5 million reads. Functional and species interaction-related features are much more sensitive (**e**-**h**).

Click here to access/download;Figure;Figure1.png

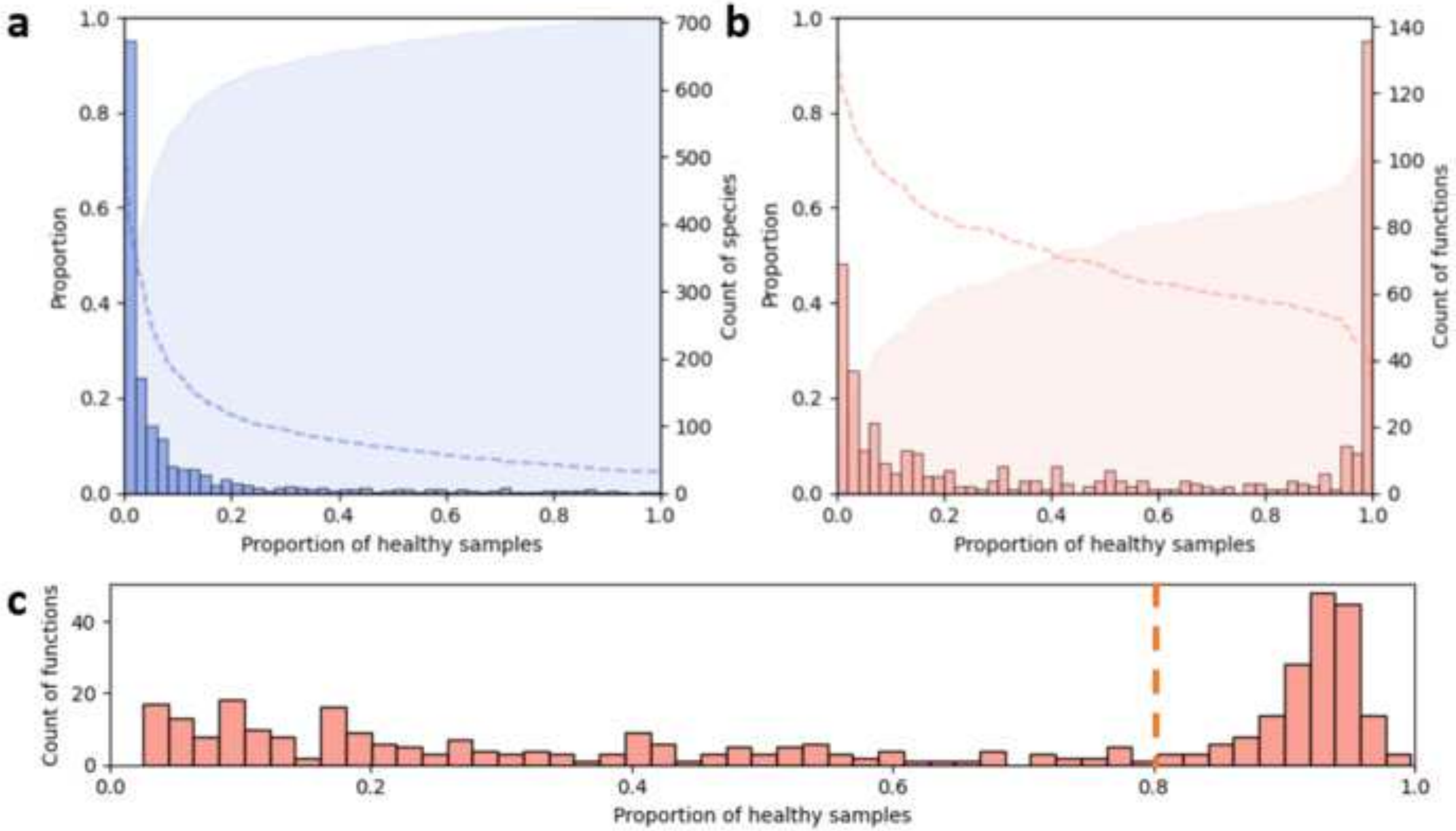

Click here to access/download;Figure;Figure 2.png

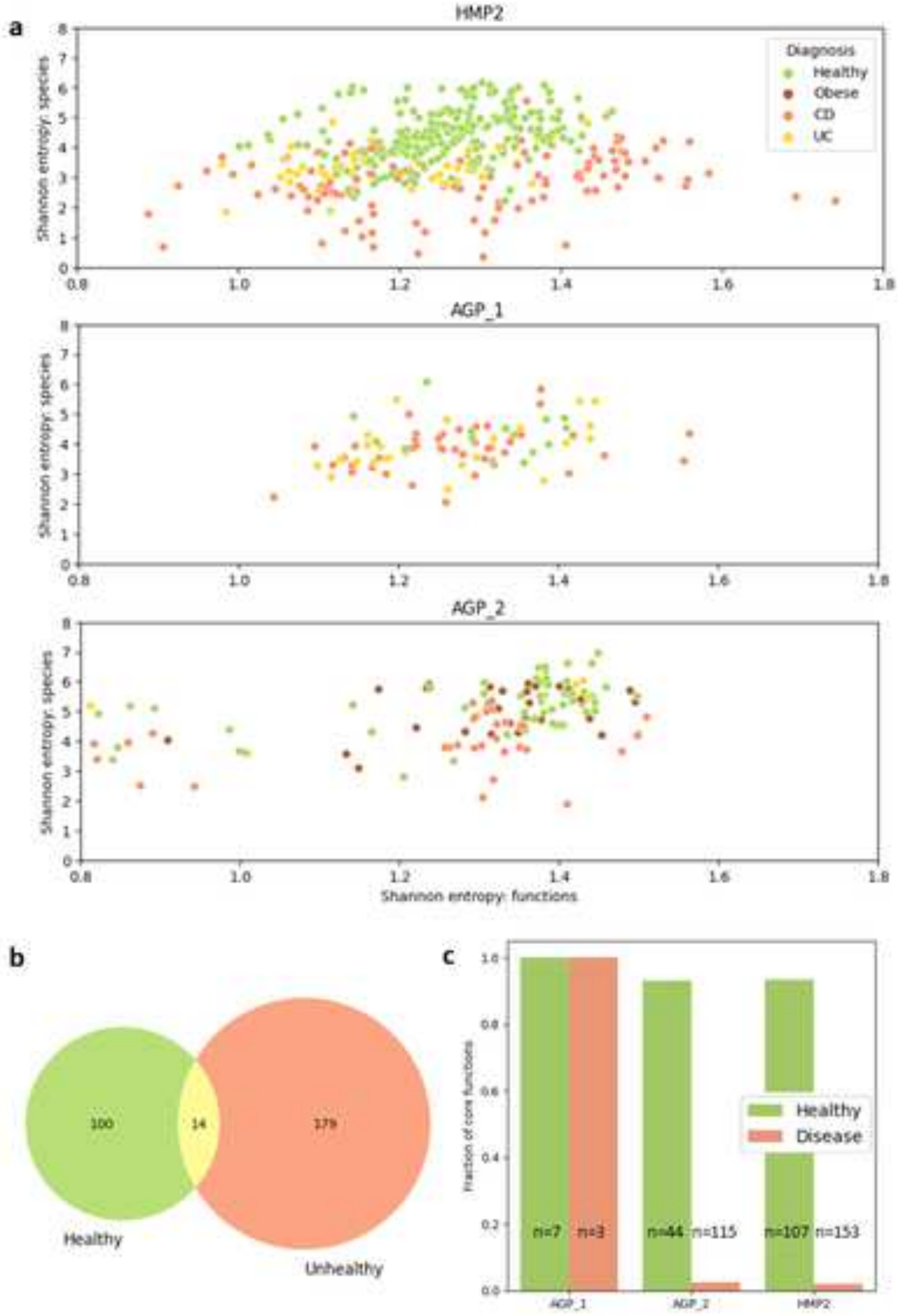

Click here to access/download;Figure;Figure 3.png

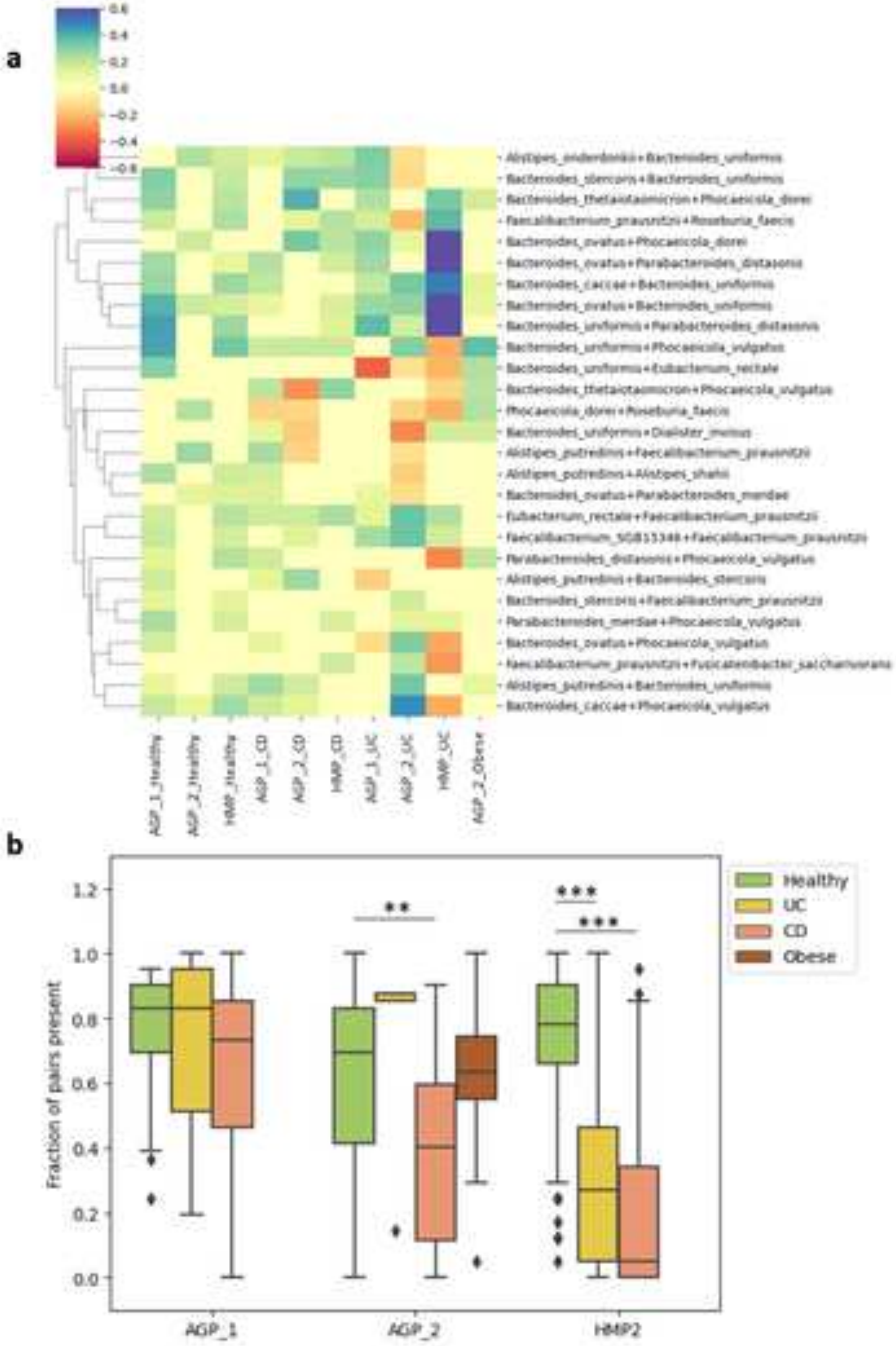

Click here to access/download;Figure;Figure 4.png

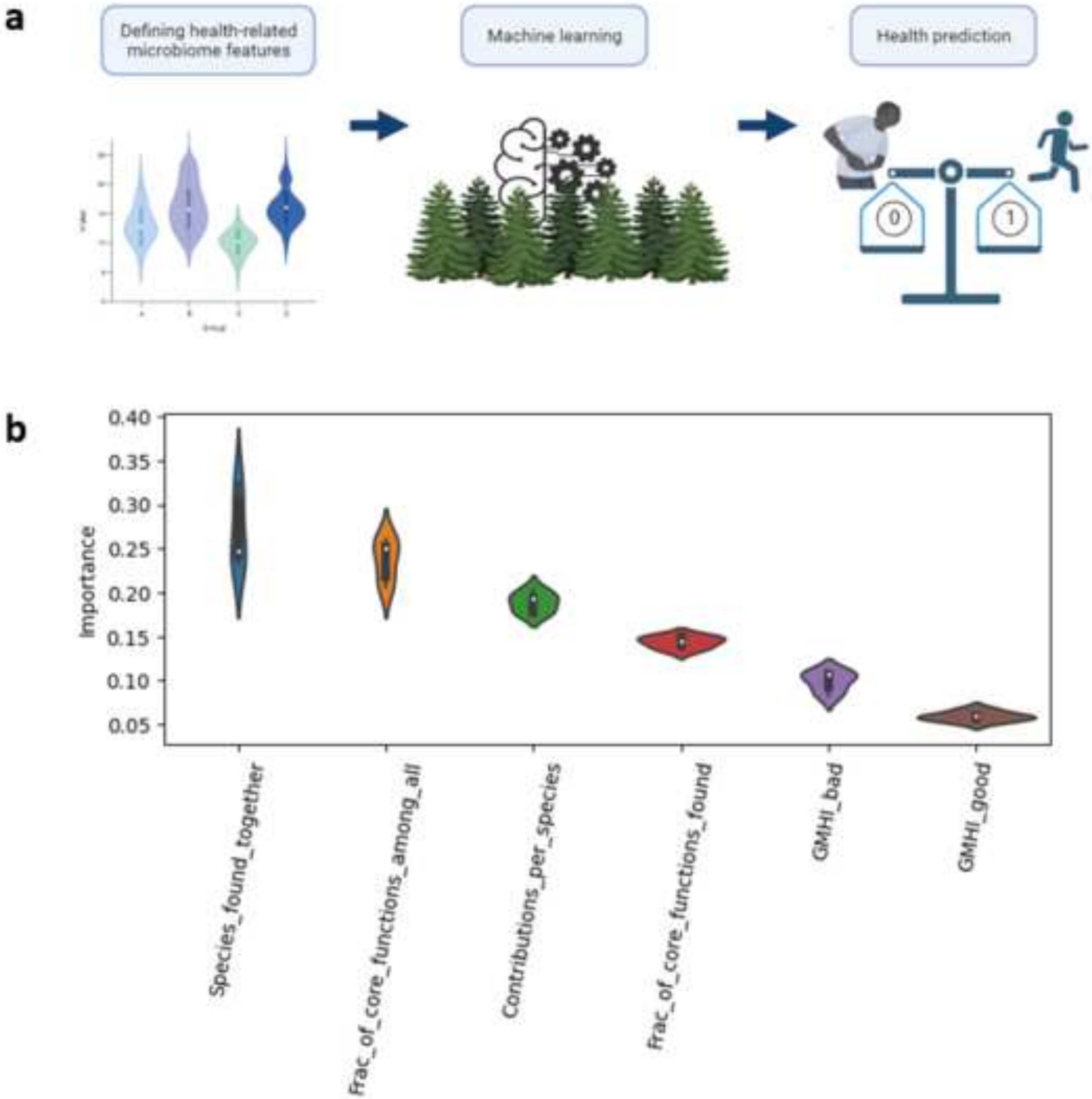

Click here to access/download;Figure;Figure 5.png

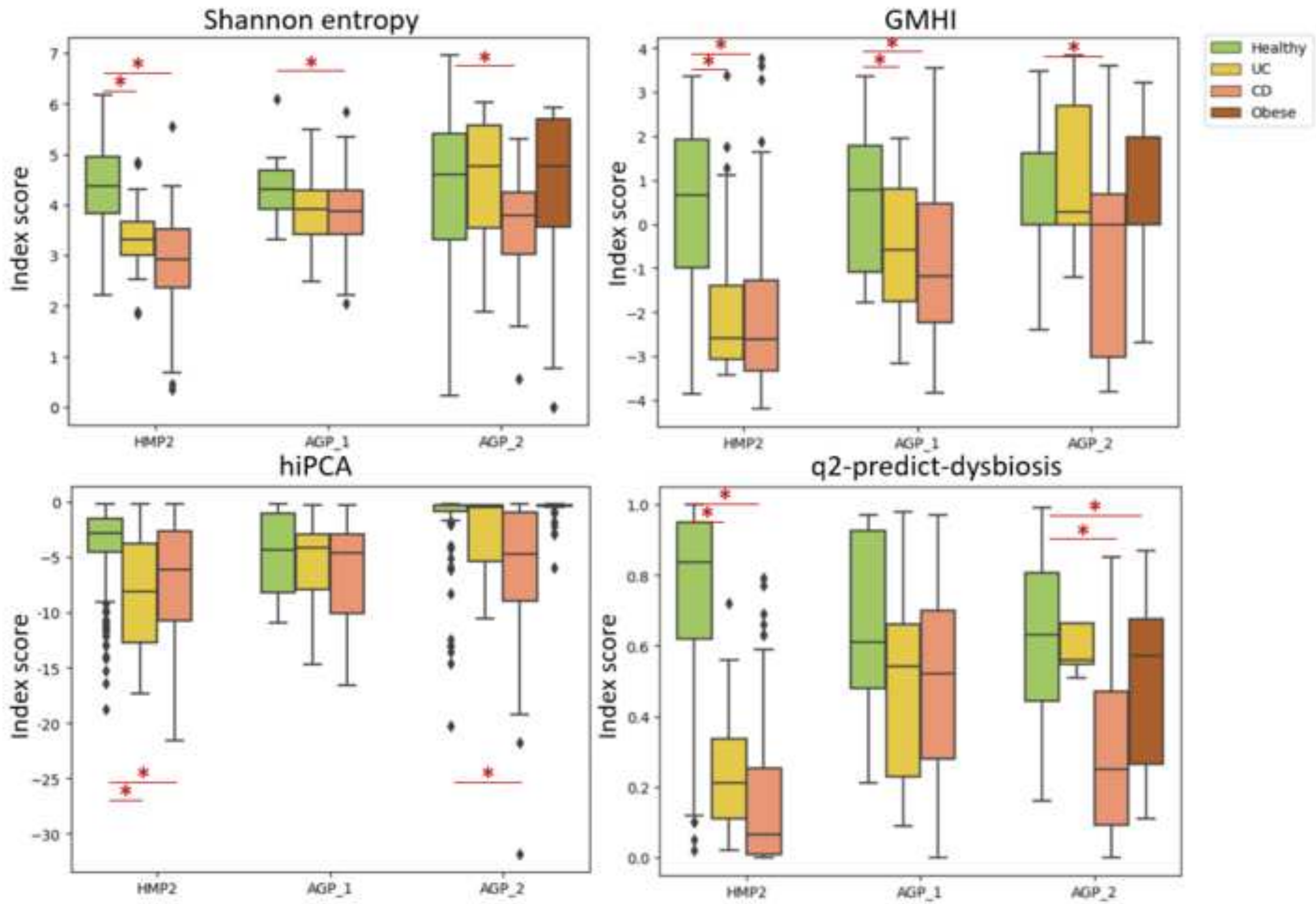

Click here to access/download;Figure;Figure 6.png

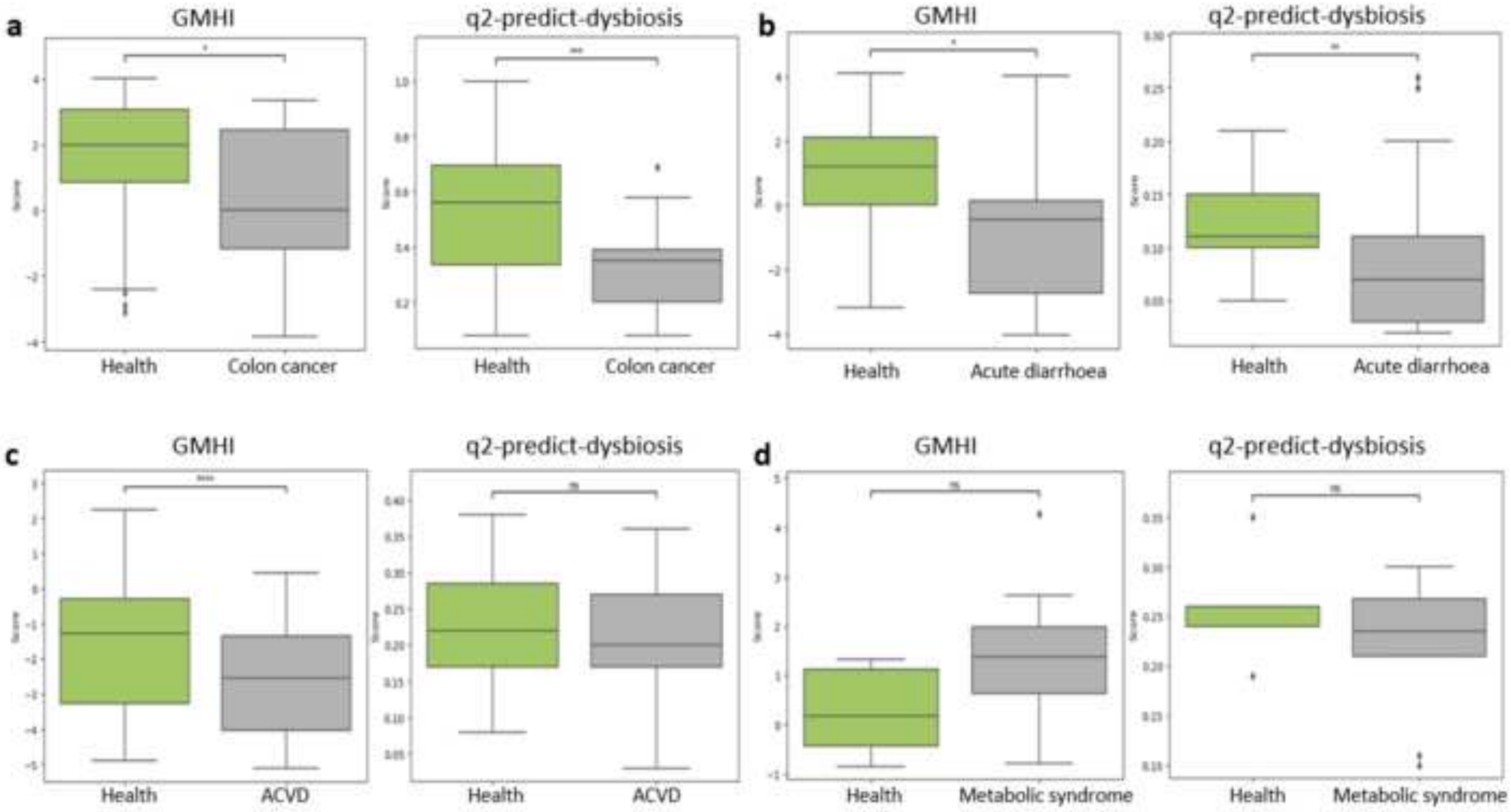

Click here to access/download;Figure;Figure 7.png

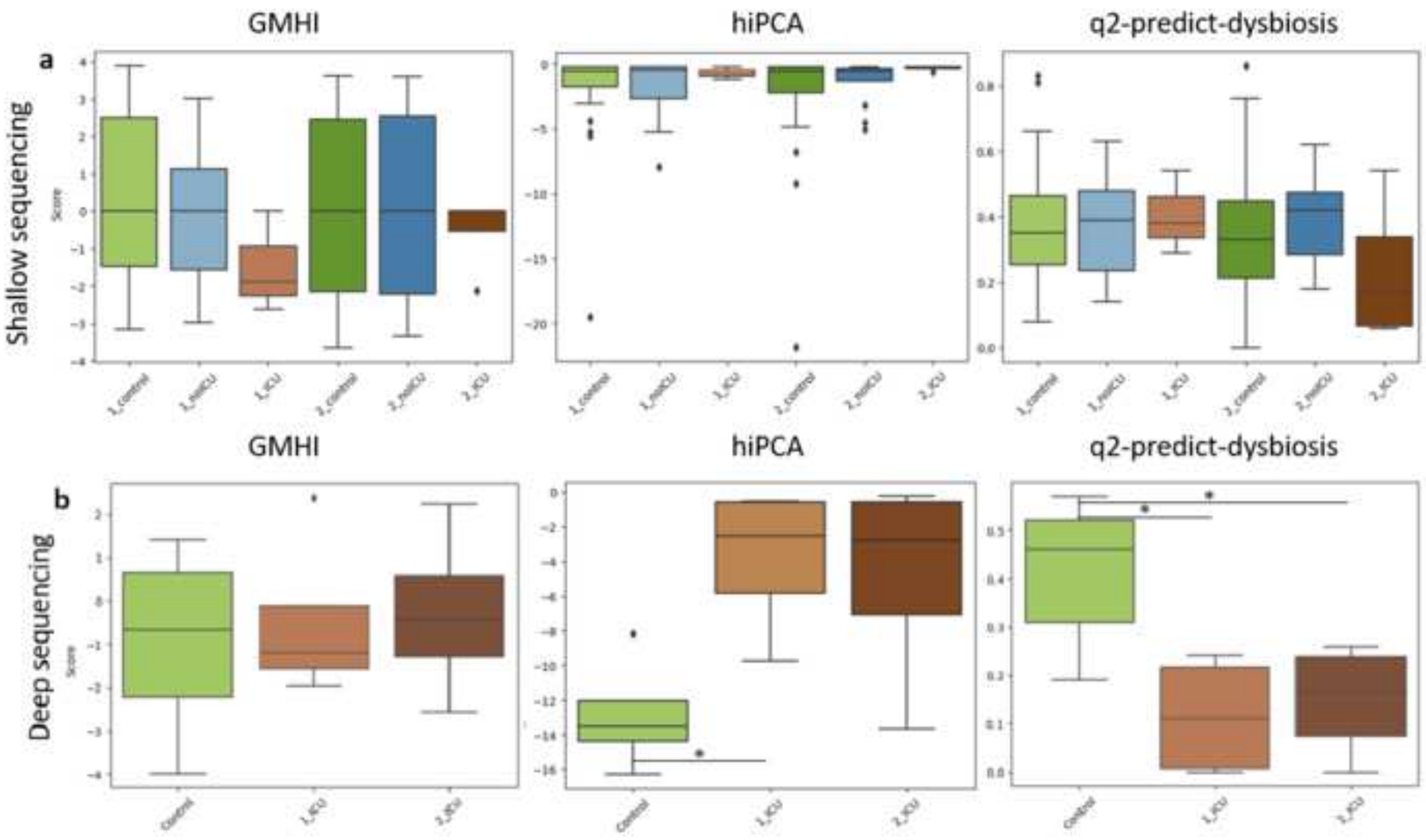

Click here to access/download;Figure;Figure 8.png

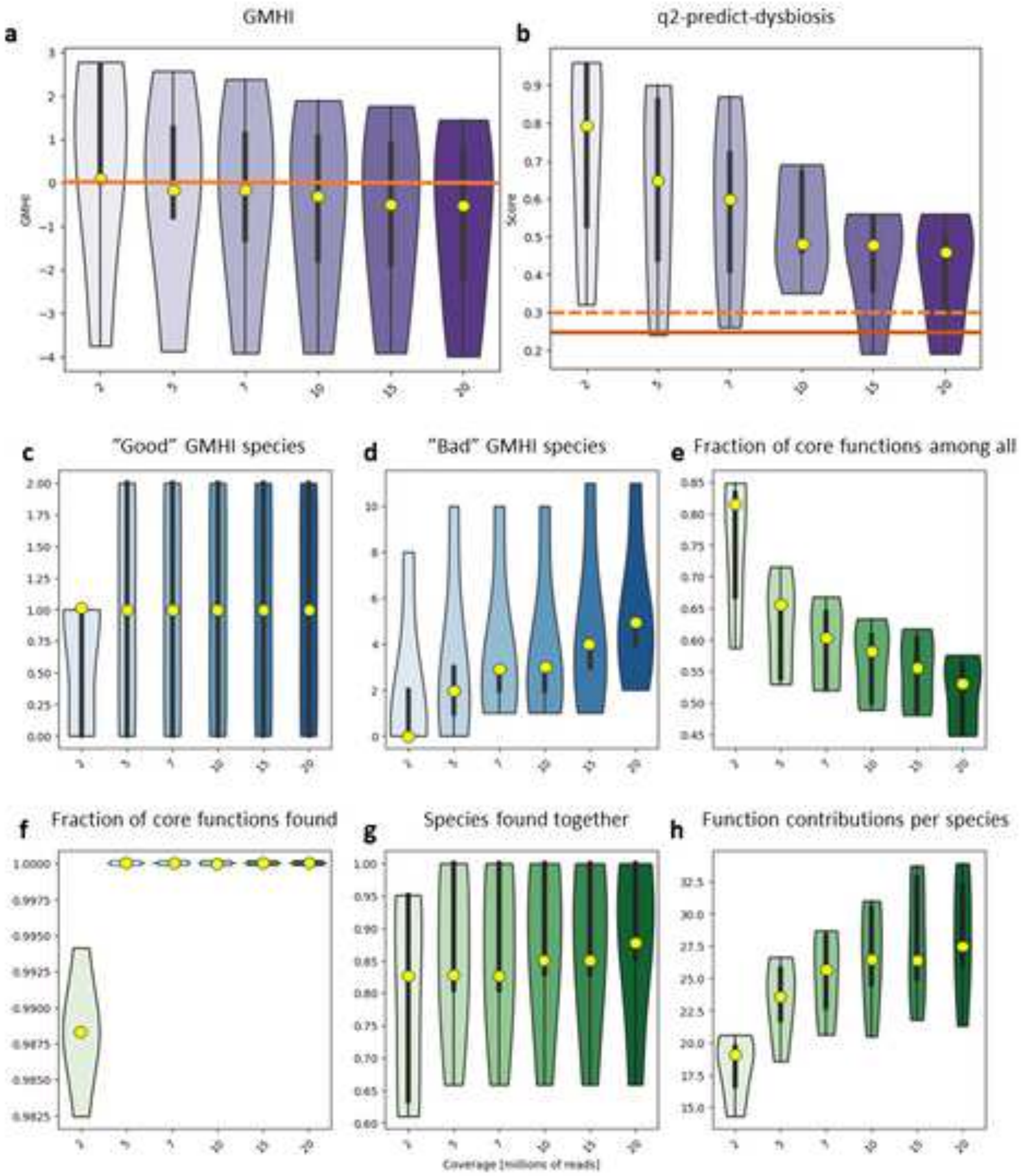

Click here to access/download
Supplementary Material
Manuscript_supplement.docx

Click here to access/download;Personal Cover;CoverLetter_GigaScience_202407_PPLsig.pdf

Prof Paweł P Łabaj, PhD DSc
Head of Bioinformatics RG
Deputy Director for General Affairs

Małopolska Centre of Biotechnology
Jagiellonian University
ul. Gronostajowa 7A
30-387 Kraków, Poland

July 23, 2024

JAGIELLONIAN UNIWERSITY IN KRAKÓW

MALOPOLSKA CENTRE OF BIOTECHNOLOGY

To the editors at *GigaScience*,

Dear Editor,

We are very excited to share this study we offer for publication consideration in *GigaScience*.

As awareness of the relevance of the gut microbiome to human health continues to increase, phrases such as "the gut-brain axis" or "microbiome dysbiosis" become only more relevant. It is clear that human microbiomes are continuously influenced by the exposome or external environment and its constituents from birth to death. Substantial resources have been invested into the understanding of the degree to which the exposome influences our guts, more specifically, into the identification of microbiome markers of health and disease. As costs of faecal microbiome analysis continuously decrease, this non-invasive procedure becomes a feasible option for patient health assessment. Despite this, **identification of healthy and diseased states is elusive as statistical analyses remain underdeveloped**. We offer a remedy to this identification problem with this submission. We utilize metagenomic metabolic and ontology-derived functions to successfully distinguish between healthy and Inflammatory Bowel Disease (IBD) individuals. This is possible from low- and high-resolution WGS metagenomic sequencing data.

Below, we provide further detail on this approach, it's novelty, timeliness and high profile, as well as reasoning for why we feel it belongs in *GigaScience*.

A rich microbiome is (debatably) considered healthy – and most approaches to assessing microbiome health rely on measures of microbiome diversity. The most comprehensive metagenomic method to date goes one step further taking into account the taxonomy of the microbes present in faecal samples. The Gut Microbiome Health Index, or the `GMHI`, was published in *Nature Communications* (Gupta et al., 2020) and has since exceeded 46.000 views. However, when applied to IBD datasets from the *Human Microbiome Project 2* (HMP2) and *American Gut Project* (AGP), it fails to distinguish between healthy and diseased samples. The same applies to its successor, the `hiPCA` (Zhu et al., 2023), which expands on the `GMHI` by applying additional data transformations.

Looking at the microbiome through the taxonomic classification lens is presently considered outdated. The functional composition of the microbial genomes themselves with identifiable pathways and metabolite outcomes provides the ecological basis of microbial co-existence and prompts the inclusion of this added information. We accommodate this demand by **re-defining microbiome health as a product of its functions and**

Address:
Gronostajowa 7a st.
30-387 Kraków

JAGIELLONIAN
UNIWERSITY
IN KRAKÓW

MALOPOLSKA
CENTRE
OF BIOTECHNOLOGY

interactions. Noting that functions are more conserved than taxa across healthy populations, **we identified a core set of functions**, rather than species, as a healthy microbiome characteristic. We observed how this core set is lost and replaced by others in the diseased state, as well as changes in the number of microbial interactions once the microbiome loses its homeostasis. Ultimately, **following the incorporation of other function- and interaction-based features, our novel IBD index was created**. Despite targeting healthy and IBD individuals from the HMP2 and AGP, classified better with our method than with the **GMHI** and the **hiPCA**, our index proved to be superior when applied to other diseases that the latter indices had been developed on. In addition, **we demonstrated its ability to identify longitudinal health trends and robustness to sequencing depth**, which the **GMHI** lacked. In short, we successfully designed and implemented the first function-based IBD-focused index of human gut health, but simultaneously achieved high levels of accuracy on other diseases. We surmise that this progress will facilitate clinical use in tracking individual health and as an early screening of gut health alterations.

We sincerely hope that you will consider this manuscript for publication in *GigaScience*.

Corresponding author: Paweł P. Łabaj

Address:
Gronostajowa 7a st.
30-387 Kraków
