# Supplementary material for: Healthy microbiome—moving towards functional interpretation
Source: Gigascience. 2025 Mar 21;14:giaf015. doi: 10.1093/gigascience/giaf015 (PMC11927397; doi:10.1093/gigascience/giaf015)
Supplement: giaf015_GIGA-D-24-00311_Revision_2 [file giaf015_giga-d-24-00311_revision_2.pdf]

GigaScience

Healthy microbiome - moving towards functional interpretation

--Manuscript Draft--

**Manuscript Number:**	GIGA-D-24-00311R2
**Full Title:**	Healthy microbiome - moving towards functional interpretation
**Article Type:**	Technical Note
**Funding Information:**	Narodowe Centrum Nauki (2020/38/E/NZ2/00598) — Dr Paweł P Łabaj
**Abstract:**	Microbiome-based disease prediction has significant potential as an early, non-invasive marker of multiple health conditions linked to dysbiosis of the human gut microbiota, thanks in part to decreasing sequencing and analysis costs. Microbiome health indices and other computational tools currently proposed in the field often are based on a microbiome's species richness and are completely reliant on taxonomic classification. A resurgent interest in a metabolism-centric, ecological approach has led to an increased understanding of microbiome metabolic and phenotypic complexity revealing substantial restrictions of taxonomy-reliant approaches. In this study, we introduce a new metagenomic health index developed as an answer to recent developments in microbiome definitions, in an effort to distinguish between healthy and unhealthy microbiomes, here in focus, inflammatory bowel disease (IBD). The novelty of our approach is a shift from a traditional Linnean phylogenetic classification towards a more holistic consideration of the metabolic functional potential underlining ecological interactions between species. Based on well-explored data cohorts, we compare our method and its performance with the most comprehensive indices to date, the taxonomy-based Gut Microbiome Health Index (GMHI), and the high dimensional principal component analysis (hiPCA)methods, as well as to the standard taxon-, and function-based Shannon entropy scoring. After demonstrating better performance on the initially targeted IBD cohorts, in comparison with other methods, we retrain our index on an additional 27 datasets obtained from different clinical conditions and validate our index's ability to distinguish between healthy and disease states using a variety of complementary benchmarking approaches. Finally, we demonstrate its superiority over the GMHI and the hiPCA on a longitudinal COVID-19 cohort and highlight the distinct robustness of our method to sequencing depth. Overall, we emphasize the potential of this metagenomic approach and advocate a shift towards functional approaches in order to better understand and assess microbiome health as well as provide directions for future index enhancements. Our method, q2-predict-dysbiosis (Q2PD), is freely available (https://github.com/Kizielins/q2-predict-dysbiosis).
**Corresponding Author:**	Paweł P Łabaj, PhD, DSc<br>Jagiellonian University: Uniwersytet Jagiellonski w Krakowie<br>Krakow, POLAND
**Corresponding Author Secondary Information:**	
**Corresponding Author's Institution:**	Jagiellonian University: Uniwersytet Jagiellonski w Krakowie
**Corresponding Author's Secondary Institution:**	
**First Author:**	Kinga Zielinska
**First Author Secondary Information:**	
**Order of Authors:**	Kinga Zielinska
	Klas I. Udekwu
	Witold Rudnicki
	Alina Frolova
	Paweł P Łabaj
**Order of Authors Secondary Information:**	

Powered by Editorial Manager® and ProduXion Manager® from Aries Systems Corporation

Response to Reviewers:	Thank you for the further feedback. We have now revised the Manuscript accordingly.
Additional Information:	
Question	Response
Are you submitting this manuscript to a special series or article collection?	Yes
Please select an option from the menu: as follow-up to "Are you submitting this manuscript to a special series or article collection?"	Functional Metagenomics
**Experimental design and statistics**<br><br>Full details of the experimental design and statistical methods used should be given in the Methods section, as detailed in our Minimum Standards Reporting Checklist. Information essential to interpreting the data presented should be made available in the figure legends.<br><br>Have you included all the information requested in your manuscript?	Yes
**Resources**<br><br>A description of all resources used, including antibodies, cell lines, animals and software tools, with enough information to allow them to be uniquely identified, should be included in the Methods section. Authors are strongly encouraged to cite Research Resource Identifiers (RRIDs) for antibodies, model organisms and tools, where possible.<br><br>Have you included the information requested as detailed in our Minimum Standards Reporting Checklist?	Yes
**Availability of data and materials**<br><br>All datasets and code on which the conclusions of the paper rely must be either included in your submission or deposited in publicly available repositories	Yes

(where available and ethically appropriate), referencing such data using a unique identifier in the references and in the "Availability of Data and Materials" section of your manuscript.<br><br>Have you have met the above requirement as detailed in our Minimum Standards Reporting Checklist?	

Powered by Editorial Manager® and ProduXion Manager® from Aries Systems Corporation

Manuscript

Click here to access/download;Manuscript;HealthyMicrobiome_for_producti

Healthy microbiome - moving towards functional interpretation

Kinga Zielińska[1], Klas I. Udekwu[2,3],Witold Rudnicki[4,5], Alina Frolova[6,7], Paweł P Łabaj[1*]

1 - Małopolska Centre of Biotechnology, Jagiellonian University, Krakow, Poland

2 - Department of Biological Sciences, Bioinformatics and Computational Biology Program, University of Idaho, Moscow, ID 83843, U.S.A.

3 - Swedish Environmental Epidemiology Centre, Department of Aquatic Sciences and Assessment, Swedish University of Agricultural Sciences, Uppsala, SE75007, Sweden

4 – Faculty of Computer Science, University of Białystok, Białystok, Poland

5 - Interdisciplinary Centre for Mathematical and Computational Modelling, University of Warsaw, Warsaw, Poland

6 - Institute of Molecular Biology and Genetics of National Academy of Sciences of Ukraine, Kyiv, Ukraine

7 - Kyiv Academic University, Kyiv, Ukraine

*corresponding author.

E-mail:

Kinga Zielinska: kinga.zielinska@uj.edu.pl
Klas I Udekwu: klas.udekwu@slu.se
Witold Rudnicki: w.rudnicki@uwb.edu.pl
Alina Frolova: fshodan@gmail.com
Paweł P Łabaj: pawel.labaj@uj.edu.pl

ORCID iDs:
Kinga Zielinska: 0000-0003-3278-1190
Klas I Udekwu: 0000-0002-8434-0744
Witold Rudnicki: 0000-0002-7928-4944
Alina Frolova: 0000-0001-5469-7366
Paweł P Łabaj: 0000-0002-4994-0234

// Abstract

Background

Microbiome-based disease prediction has significant potential as an early, non-invasive marker of multiple health conditions linked to dysbiosis of the human gut microbiota, thanks in part to decreasing sequencing and analysis costs. Microbiome health indices and other computational tools currently proposed in the field often are based on a microbiome's species richness and are completely reliant on taxonomic classification. A resurgent interest in a metabolism-centric, ecological approach has led to an increased understanding of microbiome metabolic and phenotypic complexity revealing substantial restrictions of taxonomy-reliant approaches.

Findings

In this study, we introduce a new metagenomic health index developed as an answer to recent developments in microbiome definitions, in an effort to distinguish between healthy and unhealthy microbiomes, here in focus, inflammatory bowel disease (IBD). The novelty of our approach is a shift from a traditional Linnean phylogenetic classification towards a more holistic consideration of the metabolic functional potential underlining ecological interactions between species. Based on well-explored data cohorts, we compare our method and its performance with the most comprehensive indices to date, the taxonomy-based Gut Microbiome Health Index (`GMHI`), and the high dimensional principal component analysis (`hiPCA`) methods, as well as to the standard taxon-, and function-based Shannon entropy scoring. After demonstrating better performance on the initially targeted IBD cohorts, in comparison with other methods, we retrain our index on an additional 27 datasets obtained from different clinical conditions and validate our index's ability to distinguish between healthy and disease states using a variety of complementary benchmarking approaches. Finally, we demonstrate its superiority over the `GMHI` and the `hiPCA` on a longitudinal COVID-19 cohort and highlight the distinct robustness of our method to sequencing depth.

Conclusions

Overall, we emphasize the potential of this metagenomic approach and advocate a shift towards functional approaches in order to better understand and assess microbiome health as well as provide directions for future index enhancements. Our method, `q2-predict-dysbiosis (Q2PD),` is freely available (https://github.com/Kizielins/q2-predict-dysbiosis).

Keywords

microbiome, gut microbiome health, health index, dysbiosis, metagenomics

Introduction

The prevalence of a range of diseases and conditions peripherally or directly linked to microbiome health such as Inflammatory Bowel Disease (IBD), diabetes, obesity and even various cancers, continue to increase globally and substantial funds are currently spent on diagnosis and treatment [1],[2]. While a correlation between gut microbiome composition and human health is widely acknowledged [3], the accurate identification of microbial and host markers of disease states remains elusive. Accordingly, the ability to evaluate patient health status based on a gut microbiome snapshot would be of high clinical value. Stool-based methods are promising because they can be collected non-invasively and frequently, and analysis time is short. Furthermore, decreasing costs of stool-sample analysis via next generation sequencing makes such microbiome characterization a strong competitor as a diagnostic tool [4].

Dysbiosis, defined as a perturbation of gut homeostasis, is believed to be accompanied by reduced microbiota diversity and increased prevalence of 'harmful' bacteria in adults [5],[6]. Eubiosis (opposite of dysbiosis) can be perturbed by a wide range of factors including infection, diet, exercise, antibiotics, stress or poor sleep [7]. The simplest interventions currently applied for the prevention or alleviation of mild microbiome dysbioses include dietary modification or prebiotics (often non-digestible food types which promote the growth of beneficial microorganisms), ingested live bacteria or probiotics

(beneficial bacteria usually in capsules), and lifestyle changes. More severe cases of gut dysbiosis, failing to respond to the above interventions, may qualify for fecal microbiota transplants, FMTs, which are increasingly gaining traction in clinics worldwide [8]. However, host-microbiota and intra-microbiota interactions are both extremely complex and highly individual, and despite success with FMT's, we have as yet no real understanding of why or how they work.

There are a number of accepted approaches used for the evaluation of a given gut microbiome's health status based on stool composition. Alpha diversity (Shannon entropy, for example) is a frequent choice, as microbiome richness was long believed to be a key driver of microbiome health and robustness [9],[10]. Beta diversity has also been applied in a number of longitudinal studies, albeit to a lesser degree and mainly to identify eubiotic samples based on a time-resolved proximity to other healthy samples [11]. The most robust index to date, outperforming diversity indices, is the Gut Microbiome Health Index, or the **GMHI** [12], recently renamed the Gut Microbiome Wellness Index (**GMWI**). An updated version of this index has recently been published [13]. The GMHI is based on the ratio of 50 microbial species associated with healthy or unhealthy gut ecosystems and is reported to exceed 73% accuracy in determining disease state, thus the authors suggesting that gut taxonomic signatures can predict health status. Another metagenomic gut-health index expanding on the **GMHI** approach, **hiPCA** [14], was introduced as a monitoring framework for personalized health purposes. The personalized approach is achieved by analyzing the contribution of each bacterium to the index, which allows for the identification of high-influence (ostensibly keystone) species in different patient groups. The **hiPCA** claim of better performance than the **GMHI** is attributed to the authors' application of additional transformation and clustering algorithms. Importantly, such studies are often defined by datasets limited in scope to industrialized nations and thus a less than complete consideration of diet-environment-microbiome interactions.

However, a recently re-visited definition of the microbiome emphasizes the importance of not just the microbiota (a community of microorganisms), but the whole "Theatre of Activity", ToA [15]. This ToA

includes structural elements (proteins, lipids, polysaccharides), metabolites and environmental conditions [16]. It is tightly bound to its corresponding ecological niche, and the synergistic relations between species provide all the necessary, community defining components. Based on this definition, we maintain that an index constructed from taxonomy alone is hardly sufficient to accurately capture biological phenomena occurring within the gut environment – the key to understanding gut dysbiosis. Instead, we hypothesize that **to effectively determine health:** i) a metagenomic functional profile is required (microbiome phenotype); and ii) species interactions (e.g measured as co-occurrence) but not just presence should be considered.

We introduce an approach that is based on identifiable metagenomic features within ecosystems that extend beyond diversity measures and basic taxonomic information. This **function-**centricism is broached in two ways: i) directly by evaluating the functional potential within and between species and ii) indirectly by assessing co-occurrence and synergism between bacterial species. Our goal is not only to distinguish between healthy and diseased, but importantly, to also quantify the degree of dysbiosis in each sample for the given cohort. We derive the health-describing features based on an exploratory analysis of healthy samples from the Human Microbiome Project 2 (HMP2, Lloyd-Price et al., 2019) and outperform **`Shannon entropy,`** the **`GMHI`** and the **`hiPCA`** in healthy versus inflammatory bowel disease (IBD) and obese classification. The robustness of our index is further validated by corroboratively, classifying two additional IBD-focused cohorts. By retraining the IBD-specific parameters on an additional set of 30 diverse cohorts encompassing a range of diseases, we demonstrate the superior performance of our approach. Our findings reveal that function-, rather than taxonomy-based features are more informative for the accurate classification of biological samples. Additionally, our method effectively identifies longitudinal microbiome changes in COVID-19 patients, which the **`GMHI`** and the **`hiPCA`** are unable to capture, and, crucially, it is distinctly robust to sequencing depth. Our method **`q2-predict-dysbiosis (Q2PD)`** is freely available [17].

Results

High prevalence of “core functions” in health

In order to develop a strategy to assess the degree of dysbiosis in a given microbiome sample, we must define eubiosis, i.e. the healthy microbiome. We based our initial analysis (described in the Methods section) on 384 medically-determined healthy samples from the HMP2 project and identified the most prevalent species, regardless of abundance (Figure 1a). We observed that 50% of species were present in less than 5% of samples, and hardly any species were shared by all individuals. On the other hand, the prevalence of functions within the healthy population had an opposite trend - 50% of functions were already represented by at least 40% of individuals (Figure 1b), a functional redundancy unaccounted for in the **`GMHI`** (or the **`hiPCA`** which is based on it). These results convinced us further about the unsuitability of basing an index purely on the presence of “core taxa” and encouraged a shift of focus towards more prevalent functions instead.

The addition of healthy samples from two validation cohorts maintained the function distribution profile obtained solely with HMP2 samples (Figure 1c). In order to test whether the functions were universal or cohort-specific, we calculated separately the distributions for functions present in 1, 2, or all 3 cohorts. We found that all functions missing from at least 1 cohort were present in less than 10% of samples, which indicated the presence of high-prevalence functions in all three cohorts. Based on the increased occurrence of certain functions in over 80% of samples (dotted line in Figure 1c), we defined them as “core functions” (refer to Supplementary Table 1 for full list). According to MetaCyc classification, 73.5% of “core functions” were assigned as “Biosynthesis” pathways, 18.8% “Degradation/Utilization/Assimilation”, and 7.6% “Generation of Precursor Metabolites and Energy”. In addition, a few of the above were additionally classified as “Superpathways”. The classification aligned well with a previously reported high prevalence of carbohydrate and amino acid metabolism-related pathways, potentially forming the functional microbiome core [18]. A detailed analysis of the core functions identified in our study, however, is out of the scope of this manuscript.

Shannon entropy calculated on species and functions allowed for good discrimination between healthy and unhealthy samples in the HMP2 dataset (Figure 2a top). However, the trends were unclear in the two validation cohorts (Figure 2a middle and bottom), reflecting the need for more complex methodology, expanding beyond microbiome richness, in order to classify datasets without obvious separation. Diversity analyses revealed that the number of functions per sample remained similar, or even increased, during microbiome transitions from healthy state to dysbiosis in HMP2 (Supplementary Figure 1). While admittedly, this cannot be measured solely using metagenomics data, the similarity could hypothetically be due to altered expression of genes usually silenced in the eubiotic state, although this observation was not reproduced in the validation cohorts. Next, we investigated the presence of “core functions” in different groups, testing whether “core functions” are maintained or replaced by others in dysbiotic, IBD, samples. While the differences were not significant in most cases, we noted a visibly higher percentage of "core functions" and a higher percentage of all "core functions" in healthy as compared to disease samples (Supplementary Figure 2). We carried out differential enrichment analysis using LefSe [19], performed separately for each cohort, and identified 100 functions that were more abundant in healthy as compared to disease cohorts (Figure 2b, Supplementary Figure 2). Over 90% of functions enriched in healthy samples were "core functions", while they constituted less than 5% of functions enriched in the unhealthy class of validation cohort 2 and HMP2 (Figure 2c). The LEfSe analysis on validation cohort 1 revealed only 10 significantly enriched functions (7 in health and 3 in disease), all of which were core. This indicated a more heterogeneous functional landscape within this cohort.

Species interactions and function contributions in health

Corroborating results from past studies, we observed a decrease in the species abundance in dysbiotic samples (Supplementary Figure 3, [20], [21]). Having previously noted an increase in the number of functions (Supplementary Figure 1), we speculated that the remaining species may contribute to core

or new functions, forming new connections with one another. Due to the substantial number of initial connections to analyze (170 "core functions" and 1490 species present in at least two projects), we restricted the number of species to those most informative in the context of health- / disease-state separation. We chose the Multi-Dimensional Feature Selection (MDFS) algorithm, as it was the only feature selection method accounting for inter-feature interactions that we were aware of at the time of manuscript submission [22],[23]. This approach reduced the number of relevant species to 587 allowing us to eliminate noise and focus on the most important interactions (see Methods for more details about the feature selection procedure).

We then used the SparCC algorithm, designed specifically for compositional data, to investigate correlations between the MDFS-selected species in health and disease [24]. We did not observe any trends in the number of correlations, or in the fraction of positive correlations per group, that would indicate differences between the two. However, we identified opposite relationships of some species in different groups (Figure 3a). A number of species we found to be positively correlated in eubiosis and are generally considered beneficial *(e.g. Eubacterium rectale*, *Faecalibacterium prausnitzii,* and a number of *Bacteroides* species), and those relationships would be disrupted in dysbiotic groups. We observed that the prevalence of the pairs positively correlated with health was higher than in a number of disease-associated groups (Figure 3b). Due to this, we included the co-occurrence of such species as another feature of interest to aid potentially in the determination of microbiome health.

Based on our previous results, we hypothesized that the contributions of each species to functions would be relatively stable in the healthy state and less predictable in disease. To test this, we compared the contributions of MDFS-identified species to "core functions" in different groups (Supplementary Figure 4). We did not observe any differences between health and disease, despite a relatively tight clustering of the healthy groups. However, we found stronger results when exploring functional redundancy. While the average number of species per function and the average number of functions per species did not reliably separate healthy from diseased profiles (Supplementary Figure

5), the latter approach was more informative as described in detail below. This finding was congruent with our earlier suspicions of an inherent functional plasticity of microbiome structure; modulation of function altering connectivity in the interaction network, leading to a shift towards less abundant, non-core functions upon perturbation of homeostasis. It also highlighted the challenge of identifying dysbiosis based on singular features, which were never statistically significant for all cohorts – and the utility of multiple perspectives for microbiome description, in order to meaningfully classify them.

Testing the accuracy of prediction for healthy and IBD individuals

Our final set of health-defining microbiome features included the following parameters (details of how each feature was calculated can be found in Table 1):

i) the fraction of "core functions" found,
ii) the proportion of "core functions" among all functions,
iii) the proportion of co-occurrent species pairs in healthy samples,
iv) the average number of functional 'contributions' per species.

In addition, we included two parameters derived from the GMHI method – the number of "good" and "bad" GMHI species identified in a sample, which would enable us to compare between approaches. We then fed these described parameters into a machine learning model. We opted for a Random Forest classification algorithm [25] due to its robustness to imbalanced data and interpretability, and performed a leave-one-out cross-validation [25]. For the model training, validation and subsequent testing, we used taxonomic and functional profiles from the curated Metagenomics database [26] to ensure consistency and reproducibility (see Methods).

Our index demonstrated the strongest statistically significant separation between healthy and Crohn's disease or ulcerative colitis individuals across all methods evaluated (Figure 4a). Alongside hiPCA, it was one of only two approaches to achieve a statistically significant distinction in the Nielsen_2014

cohort. Overall, both our index and hiPCA exhibited comparable levels of accuracy and AUC across the three cohorts, outperforming the other methods by a notable margin (Figure 4b). By contrast, the GMHI consistently delivered mediocre results, and Shannon entropy only performed well applied to the HMP2 cohort, yielding low AUC scores (0.37–0.53) in all other cases. When index values were used as features in the Boruta algorithm [27], with health status (0/1) as the target variable, our index emerged with the highest mean and summed importance across all cohorts (Figure 4c) [27]. The above highlights the ability of our index to provide the most informative scores for health status prediction as compared to the other methods.

Beyond IBD

While only healthy and IBD individuals had erstwhile been included in the development and validation of our approach, we wondered about the applicability of the **Q2PD** to dysbiosis attributed to other diseases. We extended our dataset to another 27 additional cohorts from various disease states, equating to 30 datasets used for method validation. We did not change any parameters of the **Q2PD**, which were originally determined based on the healthy samples from the HMP2. Instead, we retrained the model with the new data and performed a leave-one-cohort-out approach to ensure a robust benchmark. The procedure placed our index at a disadvantaged position, as some of the added datasets had been used to develop and train the other methods and our approach was thus truly blind to outcome in these new cases.

Despite the disability, and gratifyingly, the **Q2PD** achieved in terms of performance, the highest average accuracy and AUC across all datasets {(AUC=0.61, accuracy=0.58) > hiPCA (AUC=0.58, accuracy=0.57) > GMHI (AUC=0.55, accuracy=0.55) > Shannon entropy on species (AUC=0.52, accuracy=0.53) > Shannon entropy on functions (AUC=0.44, accuracy=0.43)} (See Figure 5a). The consistently poor performance of both entropy-based measures suggested their highly limited utility as predictive indices.

We observed that where **Q2PD** classified a particular cohort better than the other indices, it did so with a significantly greater margin than when it lost to the other methods. Its average winning AUC margin over the hiPCA, the second best classifier, was 0.19 while the losing margin to the hiPCA was 0.09 when the hiPCA had the highest AUC. The average winning AUC margin of Q2PD against the mean of the other indices was 0.20 and 0.11 if any other of them was better. In both cases, the T-test statistics for the differences between the means of the Q2PD's winning AUC margins and that of the hiPCA or the average of others produced p-values of 0.03 and 0.02, respectively, indicating a significant classification improvement with our method in areas in which the remaining indices did not classify well. The improvement was even more striking when we excluded datasets that had been used for the training of either method. In this case, the winning margin of the Q2PD was 0.22 while the losing margin to the mean of the other indices if any of them was better was 0.09.

Our investigation into the accuracy and AUC of the indices for each cohort revealed substantial variability in terms of the classes of cohort that each index was able to classify (Supplementary Figure 6). We observed that while some cohorts such as Liss_2016 could be classified well with function-based indices (Q2PD and Shannon entropy on functions), other cohorts such as Gupta_2019 were slightly better separated with taxonomy-based indices (hiPCA, GMHI and Shannon entropy on species). An exploration of the importance of feature(s) associated with the model training on each dataset alone revealed a large amount of diversity, suggesting different kinds of information used to classify different cohorts (Figure 5b). Interestingly, when trained on the level of individual cohorts, a random forest would pick the "GMHI_bad" as its most informative parameter (scoring the lowest rank in 9 cases) and the "GMHI_good" as the worst (appearing at the bottom of the ranking 6 times). The importance of the function-based features would vary depending on the dataset, with "Contributions_per_species" winning and losing twice and the other two being consistently in the middle of the ranking.

Having observed a discrepancy between the superior Q2PD performance and the greatest importance of the "GMHI_bad" parameter, we investigated this further by testing which types of diseases were

best suited for each index to utilize. To this end, we designed a ranking method based on the number of times each index achieved the highest AUC for the most cohorts for each disease. Overall, Q2PD outperformed the other indices for 6 diseases (atherosclerotic cardiovascular disease, colon cancer, infection, metabolic disease, schizophrenia and fecal microbiota transplant: donor versus patient classification). This was followed by hiPCA for 5 (Behcet's disease, IBD, type 2 diabetes, chronic fatigue and cirrhosis), Shannon entropy on taxa for 2 (Parkinson's disease and acute diarrhoea) and GMHI for 1 (a cohort with mixed diseases). From this we also calculated an average rank for each method for all the diseases above and yet again, Q2PD ranked best (average ranking=2.39), followed by hiPCA (2.71), Shannon entropy on taxa (3.14), GMHI (3.32) and Shannon entropy on functions (3.57). The poor performance of the GMHI indicated a diminished role of the "GMHI_bad" parameter when combining all datasets, and implied a better generalization of the health status using function-based parameters.

Q2PD robustness to longitudinal alterations and sequencing depth

To construct a final model, we trained the random forest classifier on the complete set of 30 cohorts. We then took advantage of a longitudinal COVID-19 dataset that had been sequenced both shallowly and deeply, and was 'unseen' by any methods, in order to evaluate performance of Q2PD across sequencing depths. The set consisted of three groups – COVID-19 patients who, during the course of the treatment, were either i) transferred to the intensive care unit (ICU) or ii) recovered (noICU), and iii) controls (healthy hospital staff). For every individual, two timepoints were selected - "1" that was collected upon hospital admission, or in the case of staff, early in the pandemic, and "2" which was the final sample taken from each individual. As expected, sequencing data with fewer than 300,000 reads per sample fell short in accurately classifying individuals, primarily because the limited read depth did not provide sufficient coverage for comprehensive functional annotation (Figure 6a).

Surprisingly, despite its earlier described successes at classification, Q2PD was not able to distinguish between healthy and COVID-19 individuals sequenced deeply classifying ICU patients as healthy

(Figure 6b). Curious about the reasons for this, we investigated feature importance and discovered that the two taxonomic parameters “GMHI_good” and “GMHI_bad” both had negative values. This was indicative of the detrimental influence of taxonomy-based features on the performance of the model. As suspected, retraining the Q2PD without them led to the expected, correct predictions (Figure 6c) suggesting that this dataset could be classified based on functional information alone. We further validated this when we performed classification using the taxonomy-based GMHI (Figure 6d) and the hiPCA (Figure 7e), which was inaccurate for GMHI and opposite for hiPCA.

Q2PD performed poorly on the shallow COVID-19 dataset which led us to ask where the threshold lay for sequencing depth reliability. In order to investigate method robustness to depth, we applied various degrees of rarefaction to deeply sequenced control samples expecting to see similar prediction scores despite variable sequencing depth. Because the performance of the **hiPCA** (second best classifier after the Q2PD overall) on the COVID-19 cohort was worse than that of the **GMHI**, we decided to use the latter as our benchmark.

The scores produced by the Q2PD were robust and consistent regardless of sequencing depth, whereas for **GMHI**, they increased with degree of rarefaction (Figure 7a,b). Furthermore, the **GMHI** classification appeared to be only relevant for a foreboding depth of 2 million reads (score > 0), as the healthy samples were defined as unhealthy at greater sequencing depth (score < 0). Our index correctly identified healthy samples at any sequencing depth (scores were always above threshold).

We plotted the values for each feature separately and found that at low sequencing depths certain species (Figure 8 c, d) or “core functions” (Figure 8 e, f) were undetectable. The “Fraction of core features among other features” and the “Function contributions per species” were those most dependent on coverage, which was anticipated due to their dependence on low-abundance functions (Figure 8 e - h). Interestingly, the numbers of “good” and “bad” species identified at any sequencing depth covered only 32% of the complete **GMHI** list (2 and 14, versus 7 and 43 for good and bad species, respectively). In line with the underlying theme of our work and in agreement with all other presented data , this overlap argues strongly against indices that are based solely on taxonomy.

Discussion

A connection between the human gut microbiome and gut health is now well established, and a number of approaches have been employed in an effort to identify gut dysbiosis from sequence-based analysis of stool samples. Those methods are based on taxonomy and rely either on measures of microbiome richness (alpha / beta diversity) or on the presence or absence of so-called “good” and “bad” bacteria, with the health-indexes **GMHI** [12] and **hiPCA** [14] proposed formally. Hampering such approaches, however, are ecological considerations of metabolic or functional redundancies inherent within complex environments. Currently, re-defining the microbiome to include **inferred functionality** is bolstered by recent studies that highlight the importance of interactions between microbiome components and functional aspects thereof. To address the inadequacy of dogmatic *Linnéan* (taxonomy-based) approach for evaluating change in microbiomes, we developed a novel method that incorporates function in bioinformatics-based assessment of microbiome dysbioses. We show that features based on microbiome functions and interactions define a healthy microbiome more accurately. There exists a set of “core functions”, which are consistently identified as present in healthy gut microbiomes, and which disappear in the advent of dysbiosis. We compare our results to the **hiPCA**, the **GMHI** and two **Shannon entropy** measures (calculated on either species or on functions). Our index not only outperforms the other methods in the originally targeted IBD classification, but also when applied to a range of other diseases. Finally, it is robust to sequencing depth, unlike the **GMHI**, despite being based on sequencing depth-sensitive parameters.

While we here present the index **Q2PD**, we acknowledge that challenges remain prior to eventual clinical deployment as the method’s robustness for diseases other than IBD and obesity is improved. One of the most fascinating results from this work, apart from the model itself, was the finding that different parameters were of varying importance across different diseases and cohorts. This finding could have practical implications in the clinic, as it could indicate the directionality of the microbiome-disease connections. By this we mean specifically that diseases originating in the gut, e.g. IBD, are

usually associated with taxonomic shifts and thus better classified with taxonomy-based indices while diseases originating elsewhere may have (functional) effects on the gut and thus better identified with function-oriented methods. It should be noted though that the etiology of many diseases, i.e. whether they originate in the gut or not, remains unknown.

Clearly, a deeper understanding of how the identified function- and interaction-based microbiome features respond to variations in sequencing depth and quality is required. Our initial test did find that longitudinal data provides more insight into personalized "core features" of a microbiome, and this may be indicative of individual deviations from the normal. Thus, tracking microbiome changes in individuals over time may facilitate the early identification of microbiome trajectories or alterations that could help determine risk for certain conditions or diseases, potentiating personalized clinical intervention.

A caveat to this type of study is the infancy of certain fields, in particular, the availability of functional annotation software. Currently, the only widely accepted functional annotation software is HUMAnN (any version), which requires data to be processed in a particular way. In future work we plan to explore other possibilities and ultimately migrate to other inputs (such as mifaser) as well as towards a more universal solution, such as gene content [28]. Only then can recent developments in augmented functional annotation be efficiently utilized, as we already have shown in other applications [29],[30],[31].

We are also considering the introduction of a multi-omics approach to improve our predictions, combining metagenomics, metabolomics and proteomics data. With decreasing costs for each of the above, predicting patient health status based on all three -omics methods might be a reasonable option that improves accuracy and prediction in the not-too-distant future. It would also allow us to quantitatively estimate the functions directly, instead of basing our analyses on the functional potential evaluated based on metagenomics data. As a primer for our current efforts, the integration

of multiple levels could be performed using the aforementioned MDFS, by investigating relationships between the layers, or using more advanced network-based approaches such has ViLoN [32].

In conclusion, we highlight the performance and high accuracy obtained by **Q2PD**, based only on a limited number of highly relevant parameters. Despite its development to separate samples derived from healthy vs IBD individuals, its broad applicability was proven to accurately distinguish between healthy patients and individuals with other disease states and conditions. Motivated by this success we are convinced that our approach and methodology provide a better mechanistic understanding of the human gut microbiome and its health, one based on resource competition and interactions based on fundamental ecological principles that ultimately define microbial community structure.

Methods

Defining metagenomic parameters associated with health

The raw shotgun sequencing fastq files for the HMP2 and the two IBD validation cohorts used to derive metagenomic health-associated parameters were processed with **Trim Galore 0.6.10** (RRID:SCR_011847) [33] to ensure sufficient read quality. The taxonomic profiles were calculated with **MetaPhlAn 4.0.6** (RRID:SCR_004915) [34] and functional profiles with **HUMAnN 3.7** [35]. Species selection was performed using MultiDimentional Feature Selection, or the **MDFS** [22],[23] with a Benjamini-Hochberg p-value correction in a 2D mode. Selection was performed per cohort (healthy versus each of the non-healthy groups in separate **MDFS** runs), and the final list of species was the union of the MDFS results for all cohorts (corrected p-values < 0.05). Metagenomic table formatting and alpha diversity plots were done using **QIIME 2** [36] and **SparCC** correlations with the **SCNIC** plugin [37]. Plots were made using custom python scripts. Any statistical tests were calculated with the independent t-test, unless stated otherwise.

IBD index

Random Forest is a classification and regression method [25]. The algorithm utilises an ensemble of CART trees [38], in which each tree is built using different bootstrap samples of data and different random subsets of variables at each stage of the tree construction. It is a robust and versatile algorithm that works well on different types of data [39].

Metagenomic features passed to the random forest were constructed based on healthy samples from the HMP2 and two validation cohorts (Val_1 and Val_2). The features are presented in Table 1.

Table 1. Parameters of the Q2PD model.

Name	Explanation	Derivation
Frac_of_core_functions_found	fraction of "core functions" found	number of “core functions” in a sample / number of all “core functions” in our list
Frac_of_core_functions_among_all	fraction of "core functions" among all functions	number of “core functions” in a sample / number of all functions in a sample
Species_found_together	fraction of species pairs commonly occurring together in healthy samples	SparCC correlation of species > 0.1, only nonnegative correlations in healthy cohorts
Func_contributions_per_species	average number of function contributions per species	number of all species to functions contributions based on stratified output / number of all species in a sample
GMHI_good	number of “good” GMHI species	list of health-associated GMHI species
GMHI_bad	number of “bad” GMHI species	list of disease-associated GMHI species

The importance of the features was assessed using a permutation approach and 5-fold cross validation.

From this step on, we used the manually curated samples from the curatedMetagenomicsRepository to ensure consistent sample processing (MetaPhlAn [34] and HUMAnN [35], specifically we used the MetaCyc functional annotations) as well as to provide an additional layer of validation of our approach. Our manual curation excluded a samples for the following reasons: incomplete metadata, use of antibiotics or other similar drugs, repeats from the same patients, young age (newborns), or healthy samples not being fully healthy (high BMI, chronic conditions). During filtering, we set the

following thresholds: species abundance >= 0.1%, pathway coverage >= 20% and function abundance >= 0.01% - with any features below that changed to 0.

During random forest training, healthy individuals were labeled as "1" and the IBD as "0". A leave-one-out cross-validation procedure was applied to predict health scores for each sample in order to avoid model overfitting or the need to split the data into training and testing sets. The final health score is the output of the random forest's *predict_proba* method, which expresses the probability of each sample belonging to the healthy (or unhealthy) group.

The GMHI scores were calculated with the **`QIIME 2 q2-health-index`** plugin [40] and the **`hiPCA`** [14] predictions were obtained by substituting the original test file with our test samples, due to a lack of instructions about how to do it better (this could potentially result in an overlap of the train and test samples, possibly falsely increasing the **`hiPCA`**'s accuracy). The Shannon entropies were calculated separately on taxonomic and functional profiles for each sample using custom python scripts.

The GMHI was the only parameter with pre-defined threshold for health. In the case of the other methods, the optimal threshold would be defined using the Youden's J statistic calculated on the training data.

The relevance of each index in the context of IBD predictions was calculated using **`Boruta`** [27]. The Boruta algorithm is a wrapper around the Random Forest classifier. It works in the following way: a data set is extended by adding a randomly permuted copy of each original variable - a so-called shadow variable. A predictive model for the decision variable is then built using the random forest algorithm. The importance of each variable is estimated using a permutation test. Boruta collects the information about the importance and then compares the importance score of each original variable with the maximal importance achieved by a shadow variable.

The procedure is repeated multiple times, and a statistical test is performed. The variables are eventually assigned to three classes: Confirmed (better than random), Rejected (no better than

random) and Tentative (those that could not be assigned neither to Confirmed no to the Tentative class. The utility of the variable is a good indicator of the information importance carried by the variable, also in situations where the synergistic interactions are important.

In the Boruta section of our analysis, the predictions of each index were passed as parameters and the health labels (0/1) were used as decision vectors. Index importances were redefined as ranks – with the highest importance marked as rank "1".

Index testing: expanding to other diseases

For the purpose of testing, the index was re-trained on the complete set of 30 datasets. However, no parameters were modified. To ensure a reliable benchmark, a leave-one-cohort-out procedure with a 5-fold cross validation was performed. The accuracy and AUC statistics were defined separately for each test cohort based on the validation set (which was a subset of the training dataset).

The performance of our index was evaluated by calculating its winning margin over hiPCA, the second best index, and the mean of the other methods. Specifically, for each test dataset, the AUC of the hiPCA or the mean AUC of the other methods was subtracted from the AUC produced by the Q2PD. The winning margin was defined as a mean of the AUC differences, separately for cases when Q2PD won and when not.

Importance of the features contributing to the Q2PD score was evaluated by training a random forest model separately for each cohort and using a feature permutation approach. The importances were changed to ranks, with the highest ranks (lowest values) representing the greatest importance.

Final index and application to the COVID-19 dataset

The final index was trained on the complete set of 30 cohorts using 5-fold cross validation. The health threshold was defined based on a mean of thresholds defined on training data for each iteration of the leave-one-cohort-out approach and was set at 0.38 (which was very similar for all iterations).

To evaluate the indices in the context of the robustness to sequencing depth, healthy deeply sequenced samples from the COVID-19 dataset were rarefied to different sequencing depths using **`seqtk 1.4`** [41].

Data availability

The HMP2 data can be found in Sequence Read Archive (SRA) with the accession code PRJNA398089. The two IBD validation cohorts can be located under accessions PRJNA389280 and PRJEB1220. The test cohorts were downloaded from the curatedMetagenomicsData repository [26]. The samples from this repository were manually selected - we removed samples from children, reported control yet unhealthy samples, as well as those of patients undergoing antibiotic treatment. The COVID-19 cohort can be found under the accession PRJEB64515 in the European Nucleotide Archive. The Q2PD is deposited on GitHub and can be accessed along with sample accessions and scripts needed to reproduce our results here: [17]. The complete code and additional data required to reproduce our analyses have also been deposited as a GigaDB dataset [42]. DOME-ML (Data, Optimization, Model and Evaluation in Machine Learning) annotations are available in the DOME registry via accession 4cstv1dfjm [43]. An archival copy of the code is available via Software Heritage [44].

Availability of supporting source code and requirements

Project name: q2-predict-dysbiosis

Project home page: https://github.com/Kizielins/q2-predict-dysbiosis

Operating system(s): Platform independent

Programming language: Python

Other requirements: scikit-learn version >= 1.1.3

License: MIT Licence

RRID: SCR_026038

bio.tools ID: q2-predict-dysbiosis

Acknowledgements

This research was conducted as a part of the NCN Sonata BIS grant number 2020/38/E/NZ2/00598. We gratefully acknowledge Poland's high-performance Infrastructure PLGrid (HPC Centers: ACK Cyfronet AGH, PCSS, CI TASK, WCSS) for providing computer facilities. We would like to thank Dr Krzysztof Mnich, Dr Sajad Shahbazi, Dr Balakrishnan Subramanian and Piotr Stomma from the University of Białystok for their expertise in the Multi-Dimensional Feature Selection algorithm and insightful contributions to our work, dr. Tomasz Kościółek from the Sano Centre for Computational Medicine and the members of Bioinformatics Research Group at MCB JU for their comments and support.

Ethics declarations

All authors declare no competing interests.

Bibliography

[1] A. E. M'koma, 'Inflammatory Bowel Disease: An Expanding Global Health Problem', *Clin. Med. Insights Gastroenterol.*, vol. 6, p. CGast.S12731, Jan. 2013, doi: 10.4137/CGast.S12731.

[2] Y.-R. Hong, J. Huo, R. Desai, M. Cardel, and A. A. Deshmukh, 'Excess Costs and Economic Burden of Obesity-Related Cancers in the United States', *Value Health J. Int. Soc. Pharmacoeconomics Outcomes Res.*, vol. 22, no. 12, pp. 1378–1386, Dec. 2019, doi: 10.1016/j.jval.2019.07.004.

[3] J. Durack and S. V. Lynch, 'The gut microbiome: Relationships with disease and opportunities for therapy', *J. Exp. Med.*, vol. 216, no. 1, pp. 20–40, Oct. 2018, doi: 10.1084/jem.20180448.

[4] J. S. Bajaj, C. Acharya, M. Sikaroodi, P. M. Gillevet, and L. R. Thacker, 'Cost-effectiveness of integrating gut microbiota analysis into hospitalisation prediction in cirrhosis', *GastroHep*, vol. 2, no. 2, pp. 79–86, Mar. 2020, doi: 10.1002/ygh2.390.

[5] A. K. DeGruttola, D. Low, A. Mizoguchi, and E. Mizoguchi, 'Current Understanding of Dysbiosis in Disease in Human and Animal Models', *Inflamm. Bowel Dis.*, vol. 22, no. 5, pp. 1137–1150, May 2016, doi: 10.1097/MIB.0000000000000750.

[6] T. Hrncir, 'Gut Microbiota Dysbiosis: Triggers, Consequences, Diagnostic and Therapeutic Options', *Microorganisms*, vol. 10, no. 3, p. 578, Mar. 2022, doi: 10.3390/microorganisms10030578.

[7] J. E. Martinez *et al.*, 'Unhealthy Lifestyle and Gut Dysbiosis: A Better Understanding of the Effects of Poor Diet and Nicotine on the Intestinal Microbiome', *Front. Endocrinol.*, vol. 12,

2021, Accessed: Dec. 03, 2023. [Online]. Available: https://www.frontiersin.org/articles/10.3389/fendo.2021.667066

[8] M. J. Bull and N. T. Plummer, ‘Part 2: Treatments for Chronic Gastrointestinal Disease and Gut Dysbiosis’, *Integr. Med. Clin. J.*, vol. 14, no. 1, pp. 25–33, Feb. 2015.

[9] J. Li, K. L. Hoang, K. A. Bates, S. C. L. Knowles, K. C. King, and T. E. Hector, ‘Experimental temperatures shape host microbiome diversity and composition’, *Glob. Change Biol.*, vol. 29, Oct. 2022, doi: 10.1111/gcb.16429.

[10] D. Gong, X. Gong, L. Wang, X. Yu, and Q. Dong, ‘Involvement of Reduced Microbial Diversity in Inflammatory Bowel Disease’, *Gastroenterol. Res. Pract.*, vol. 2016, p. 6951091, 2016, doi: 10.1155/2016/6951091.

[11] S. Zouiouich *et al.*, ‘Markers of metabolic health and gut microbiome diversity: findings from two population-based cohort studies’, *Diabetologia*, vol. 64, no. 8, pp. 1749–1759, 2021, doi: 10.1007/s00125-021-05464-w.

[12] V. K. Gupta *et al.*, ‘A predictive index for health status using species-level gut microbiome profiling’, *Nat. Commun.*, vol. 11, no. 1, Art. no. 1, Sep. 2020, doi: 10.1038/s41467-020-18476-8.

[13] D. Chang *et al.*, ‘Gut Microbiome Wellness Index 2 enhances health status prediction from gut microbiome taxonomic profiles’, *Nat. Commun.*, vol. 15, no. 1, p. 7447, Aug. 2024, doi: 10.1038/s41467-024-51651-9.

[14] J. Zhu *et al.*, ‘Statistical modeling of gut microbiota for personalized health status monitoring’, *Microbiome*, vol. 11, no. 1, p. 184, Aug. 2023, doi: 10.1186/s40168-023-01614-x.

[15] G. Berg *et al.*, ‘Microbiome definition re-visited: old concepts and new challenges’, *Microbiome*, vol. 8, no. 1, p. 103, Jun. 2020, doi: 10.1186/s40168-020-00875-0.

[16] J.-Y. Lee, D. J. Bays, H. P. Savage, and A. J. Bäumler, ‘The human gut microbiome in health and disease: time for a new chapter?’, *Infect. Immun.*, Nov. 2024, doi: 10.1128/iai.00302-24.

[17] *q2-predict-dysbiosis*. [Online]. Available: https://github.com/Kizielins/q2-predict-dysbiosis

[18] Y. Zou *et al.*, ‘1,520 reference genomes from cultivated human gut bacteria enable functional microbiome analyses’, *Nat. Biotechnol.*, vol. 37, no. 2, Art. no. 2, Feb. 2019, doi: 10.1038/s41587-018-0008-8.

[19] N. Segata *et al.*, ‘Metagenomic biomarker discovery and explanation’, *Genome Biol.*, vol. 12, no. 6, p. R60, 2011, doi: 10.1186/gb-2011-12-6-r60.

[20] H. C. Mirsepasi-Lauridsen *et al.*, ‘Disease-Specific Enteric Microbiome Dysbiosis in Inflammatory Bowel Disease’, *Front. Med.*, vol. 5, 2018, Accessed: Dec. 03, 2023. [Online]. Available: https://www.frontiersin.org/articles/10.3389/fmed.2018.00304

[21] A. Mosca, M. Leclerc, and J. P. Hugot, ‘Gut Microbiota Diversity and Human Diseases: Should We Reintroduce Key Predators in Our Ecosystem?’, *Front. Microbiol.*, vol. 7, 2016, Accessed: Dec. 03, 2023. [Online]. Available: https://www.frontiersin.org/articles/10.3389/fmicb.2016.00455

[22] R. Piliszek *et al.*, ‘MDFS: MultiDimensional Feature Selection in R’, *R J.*, vol. 11, no. 1, pp. 198–210, 2019.

[23] K. Mnich and W. R. Rudnicki, ‘All-relevant feature selection using multidimensional filters with exhaustive search’, *Inf. Sci.*, vol. 524, pp. 277–297, Jul. 2020, doi: 10.1016/j.ins.2020.03.024.

[24] J. Friedman and E. J. Alm, ‘Inferring Correlation Networks from Genomic Survey Data’, *PLOS Comput. Biol.*, vol. 8, no. 9, p. e1002687, Sep. 2012, doi: 10.1371/journal.pcbi.1002687.

[25] L. Breiman, ‘Random Forests’, *Mach. Learn.*, vol. 45, no. 1, pp. 5–32, Oct. 2001, doi: 10.1023/A:1010933404324.

[26] E. Pasolli *et al.*, ‘Accessible, curated metagenomic data through ExperimentHub’, *Nat. Methods*, vol. 14, no. 11, Art. no. 11, Nov. 2017, doi: 10.1038/nmeth.4468.

[27] M. B. Kursa and W. R. Rudnicki, ‘Feature Selection with the Boruta Package’, *J. Stat. Softw.*, vol. 36, pp. 1–13, Sep. 2010, doi: 10.18637/jss.v036.i11.

[28] C. Zhu *et al.*, ‘Functional sequencing read annotation for high precision microbiome analysis’, *Nucleic Acids Res.*, vol. 46, no. 4, p. e23, Nov. 2017, doi: 10.1093/nar/gkx1209.
[29] M. Maranga *et al.*, ‘Comprehensive Functional Annotation of Metagenomes and Microbial Genomes Using a Deep Learning-Based Method’, *mSystems*, vol. 8, no. 2, pp. e01178-22, Mar. 2023, doi: 10.1128/msystems.01178-22.
[30] J. Koehler Leman *et al.*, ‘Sequence-structure-function relationships in the microbial protein universe’, *Nat. Commun.*, vol. 14, no. 1, Art. no. 1, Apr. 2023, doi: 10.1038/s41467-023-37896-w.
[31] L. M. Szydlowski *et al.*, ‘Adaptation to space conditions of novel bacterial species isolated from the International Space Station revealed by functional gene annotations and comparative genome analysis’, Sep. 30, 2023, *bioRxiv*. doi: 10.1101/2023.09.28.559980.
[32] M. M. Kańduła, A. D. Aldoshin, S. Singh, E. D. Kolaczyk, and D. P. Kreil, ‘ViLoN—a multi-layer network approach to data integration demonstrated for patient stratification’, *Nucleic Acids Res.*, vol. 51, no. 1, p. e6, Jan. 2023, doi: 10.1093/nar/gkac988.
[33] F. Krueger, *Trim Galore*. (Nov. 29, 2023). Perl. Accessed: Dec. 04, 2023. [Online]. Available: https://github.com/FelixKrueger/TrimGalore
[34] A. Blanco-Míguez *et al.*, ‘Extending and improving metagenomic taxonomic profiling with uncharacterized species using MetaPhlAn 4’, *Nat. Biotechnol.*, vol. 41, no. 11, Art. no. 11, Nov. 2023, doi: 10.1038/s41587-023-01688-w.
[35] F. Beghini *et al.*, ‘Integrating taxonomic, functional, and strain-level profiling of diverse microbial communities with bioBakery 3’, *eLife*, vol. 10, p. e65088, May 2021, doi: 10.7554/eLife.65088.
[36] E. Bolyen *et al.*, ‘Reproducible, interactive, scalable and extensible microbiome data science using QIIME 2’, *Nat. Biotechnol.*, vol. 37, no. 8, pp. 852–857, Aug. 2019, doi: 10.1038/s41587-019-0209-9.
[37] *SCNIC*. [Online]. Available: https://github.com/lozuponelab/q2-SCNIC
[38] J. F. Leo Breiman, *Classification and Regression Trees*. Chapman and Hall/CRC, 1984.
[39] M. Fernandez-Delgado, E. Cernadas, S. Barro, and D. Amorim, ‘Do we Need Hundreds of Classifiers to Solve Real World Classification Problems?’.
[40] *q2-health-index*. [Online]. https://github.com/bioinf-mcb/q2-health-indexhttps://github.com/bioinf-mcb/q2-health-index
[41] H. Li, *lh3/seqtk*. (Nov. 30, 2023). C. Accessed: Dec. 04, 2023. [Online]. https://github.com/lh3/seqtk
[42] Zielińska K, Udekwu KI, Rudnicki W, Frolova A, Łabaj PP, ‘Supporting data for “Healthy microbiome - moving towards functional interpretation”’. GigaScience Database, 2025. doi: https://doi.org/10.5524/102656.
[43] Zielińska K, Udekwu KI, Rudnicki W, Frolova A and Łabaj PP, ‘Healthy microbiome - moving towards functional interpretation. [DOME-ML Annotations]’. DOME-ML Registry, 2025. [Online]. https://registry.dome-ml.org/review/4cstv1dfjm
[44] Zielińska K, Udekwu KI, Rudnicki W, Frolova A and Łabaj PP, ‘Healthy microbiome - moving towards functional interpretation (Version 1). [Computer software]’. Software Heritage, 2025. [Online]. https://archive.softwareheritage.org/swh:1:snp:c8fff6424c577c1495cd04c81a21657540563131

Captions

Figure 1. Distributions of species **(a)** and functions **(b)** present in healthy samples from the HMP2; absolute values of species and function counts are shown as histograms (with scales on the right-hand side, with shaded cumulative sum in the background and an inverse of the cumulative sum represented with a dashed line (with scales on the left-hand side). **(c)** Distribution of functions in healthy individuals from the HMP2 and two validation cohorts.

Figure 2. (**a**) Shannon entropy scores for species and functions in healthy and unhealthy samples from the HMP2 and validation cohorts. (**b**) LEfSe differential enrichment analysis: overlap of enriched pathways in healthy and unhealthy individuals in the validation and HMP2 projects. (**c**) Fraction of core functions among differentially enriched functions in healthy and unhealthy individuals in the validation and HMP2 cohorts.

Figure 3. (**a**) `SparCC` correlation strengths between species, restricted to pairs which were not negatively correlated in any healthy cohort. (**b**) Prevalence of the pairs in different cohorts.

Figure 4. (a) `Q2PD`, `GMHI, hiPCA` and `Shannon entropy (on species and functions)` scores for healthy and IBD (Crohn's disease = CD, ulcerative colitis = UC) individuals. (**b**) Accuracy and AUC values for each index, per IBD cohort. ShT = Shannon entropy on taxa, ShF = Shannon entropy on functions. (**c**) Average (top) and summed (bottom) importance of each index in the context of IBD prediction, determined by Boruta.

Figure 5 (**a**) Average accuracy and AUC values for each cohort. (**b**) Average importance ranks of features for each cohort. A lower rank indicates greater importance. Cells with black borders indicate

variables identified as informative by the Boruta algorithm. The AUCs were produced as a part of the feature importances analysis when training on individual cohorts, and therefore do not align with the AUCs produced by the Q2PD. Only datasets with AUCs > 0.5 are shown.

Figure 6. (**a**) Q2PD predictions on the shallow COVID-19 cohort. (**b**) . Q2PD predictions on the deep COVID-19 cohort. (**c**) Q2PD predictions on the deep COVID-19 cohort with the taxonomic parameters excluded. GMHI (**d**) and hiPCA (**e**) predictions on the deep COVID-19 cohort.

Figure 7. Robustness of health predictions to sequencing coverage. GMHI (**a**) and Q2PD (**b**) scores for deeply sequenced healthy samples from the COVID-19 cohort, rarefied to corresponding depths. The horizontal lines represent health thresholds relevant to the corresponding methods. Taxonomic GMHI-inspired Q2PD features (**c**, **d**) remain relatively stable above the coverage of 5 million reads. Functional and species interaction-related features are much more sensitive (**e**-**h**).

Click here to access/download;Figure;Figure1.png

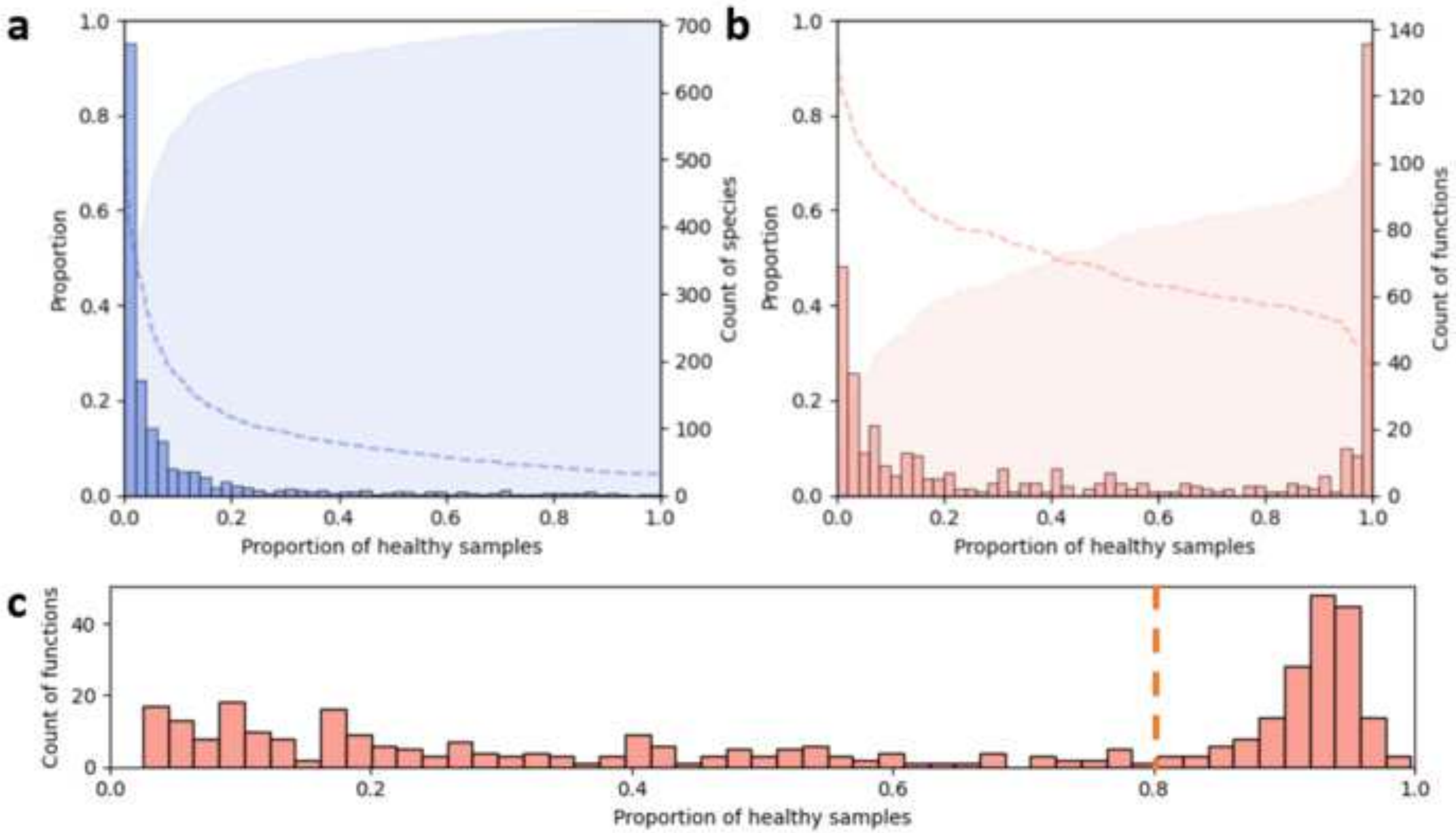

Click here to access/download;Figure;Figure 8.png

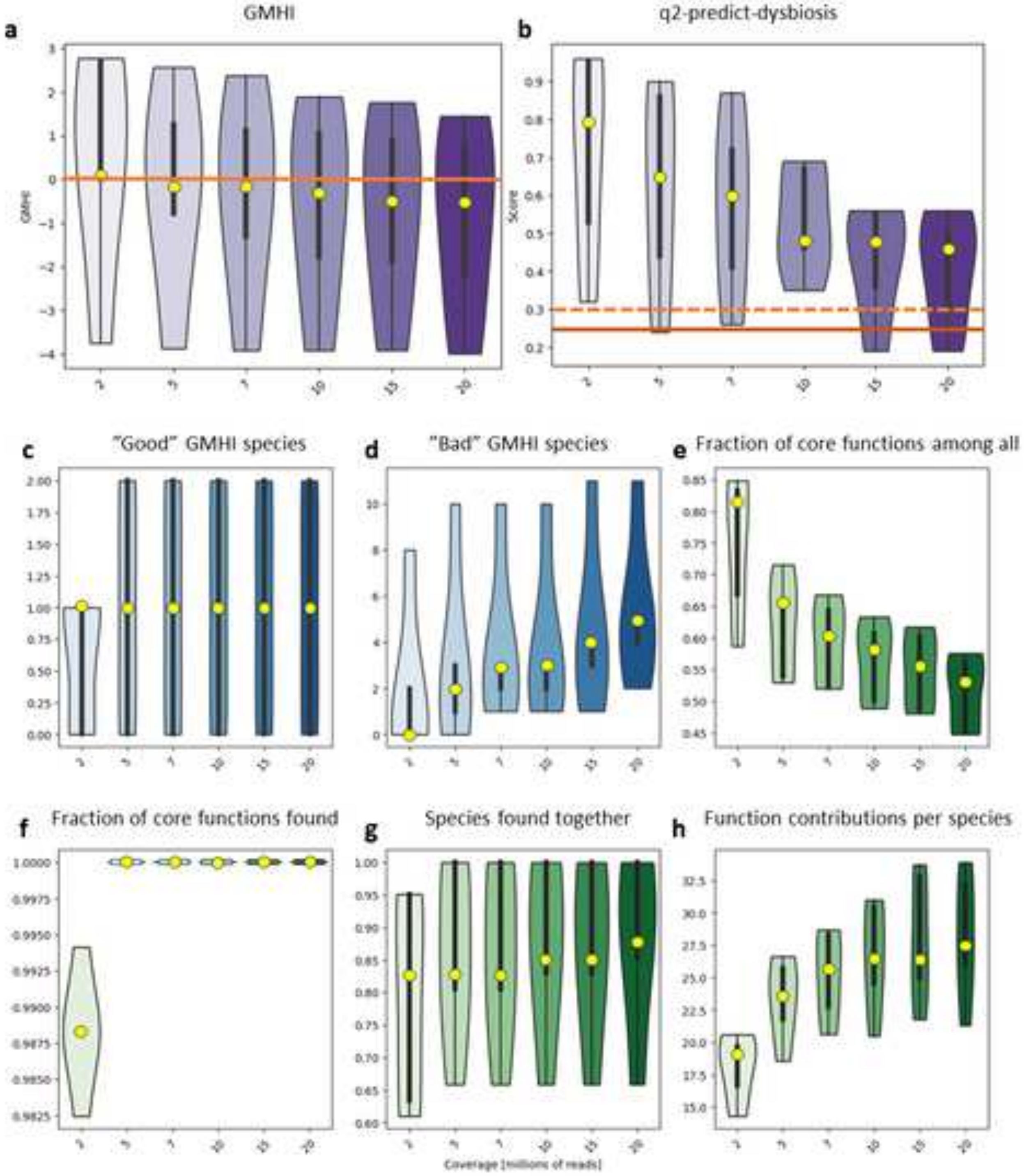

Figure 4

Click here to access/download;Figure;Figure 4.png

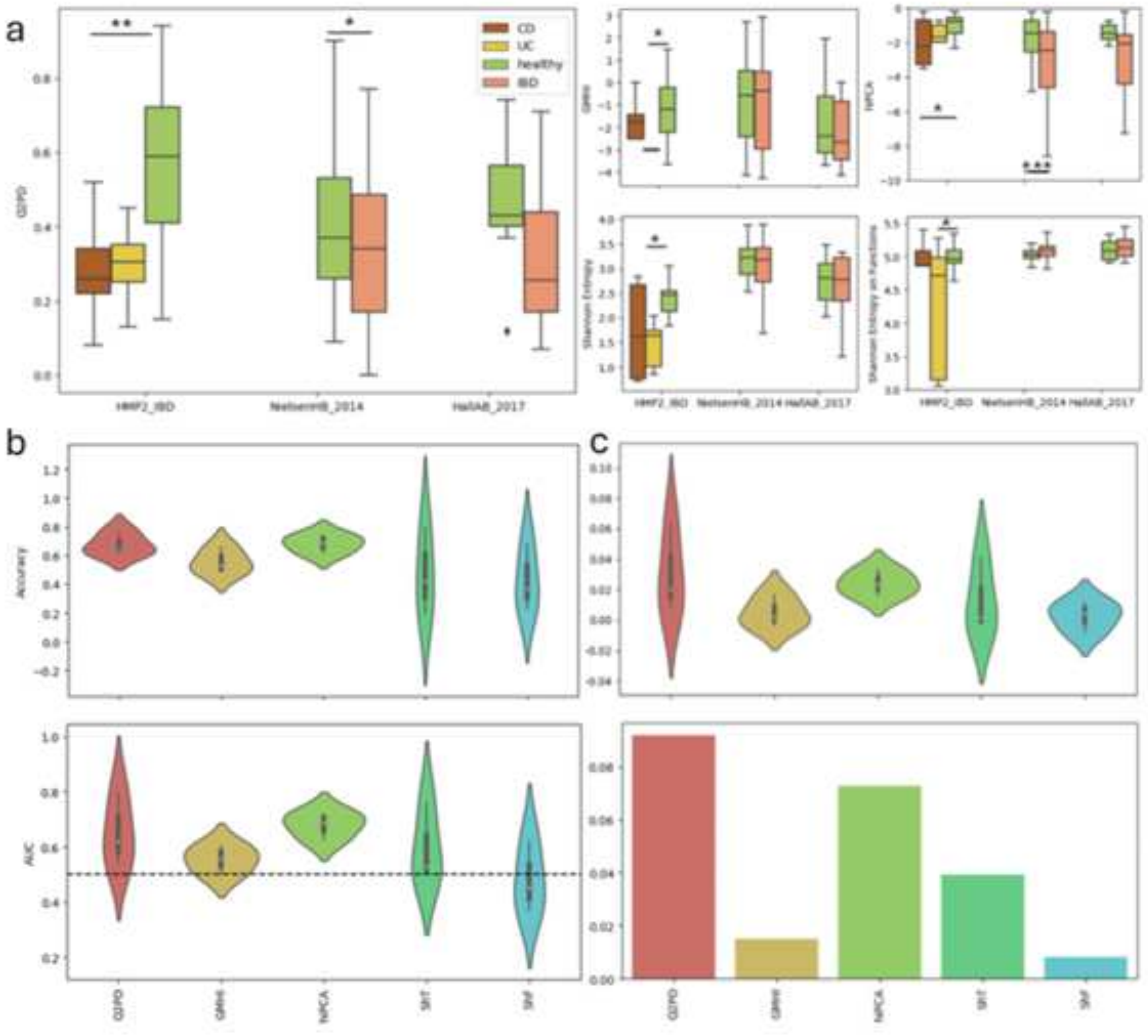

Figure 5

Click here to access/download;Figure;Figure 5.png

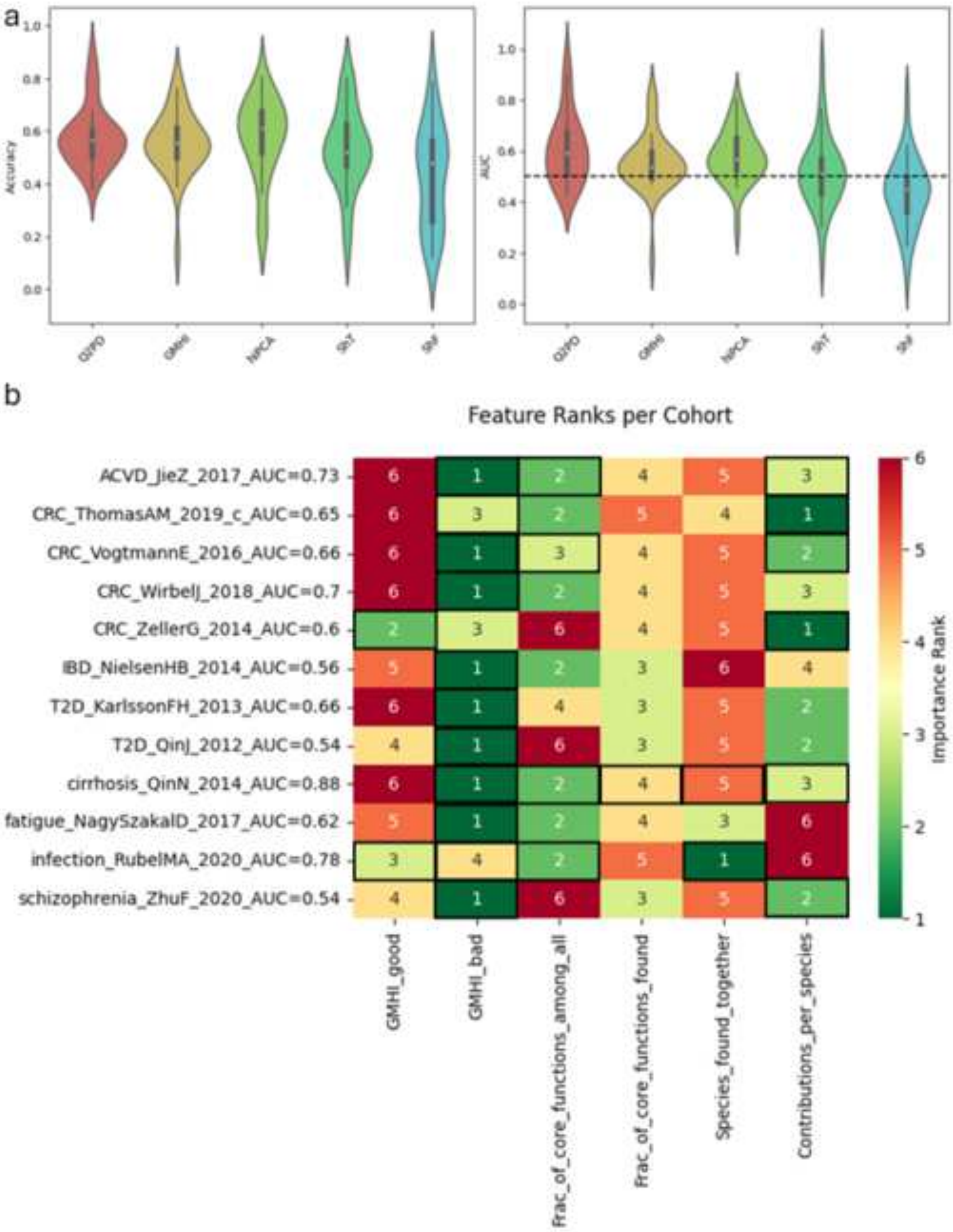

Figure 6

Click here to access/download;Figure;Figure 6.png

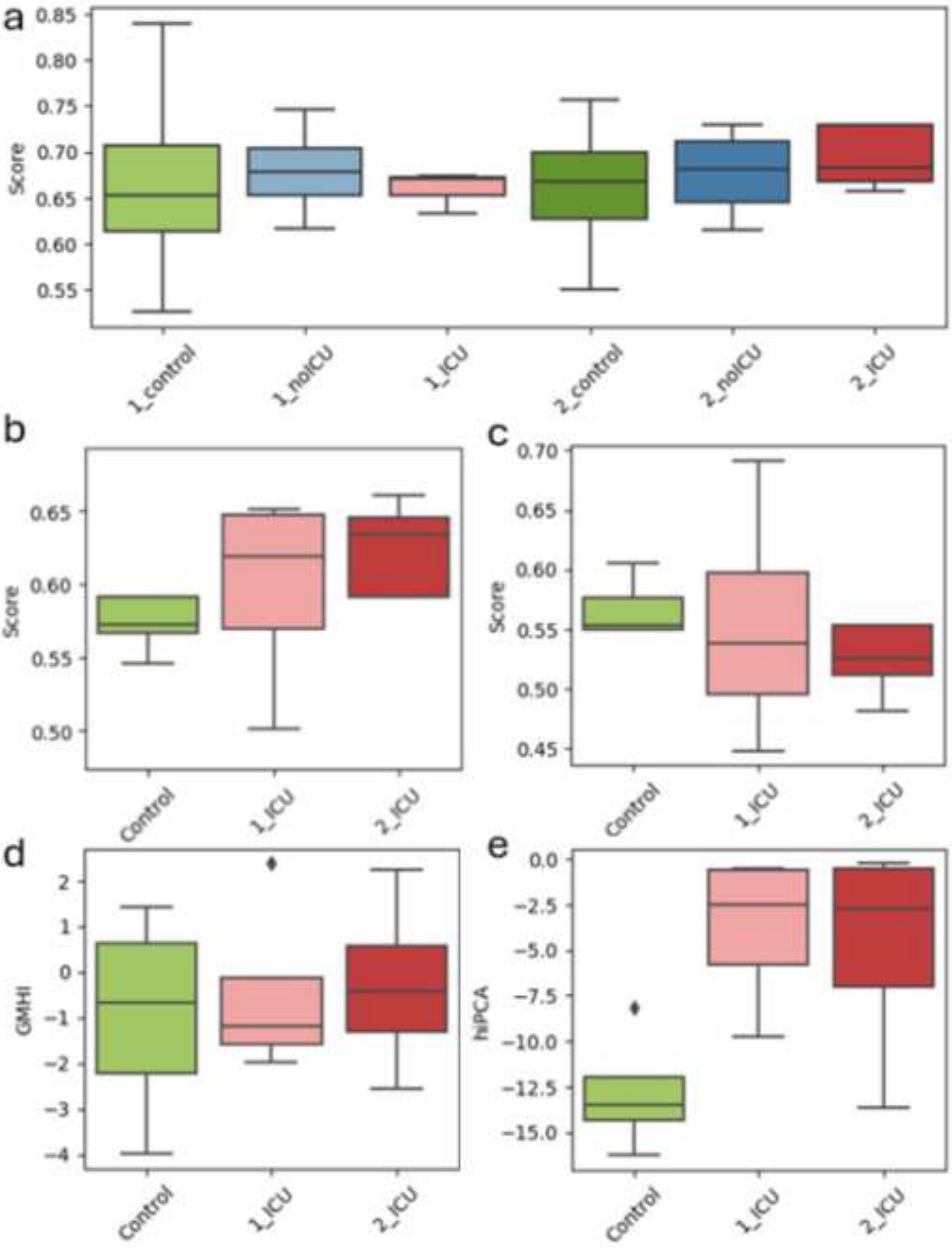

Figure 7

Click here to access/download;Figure;Figure 7.png

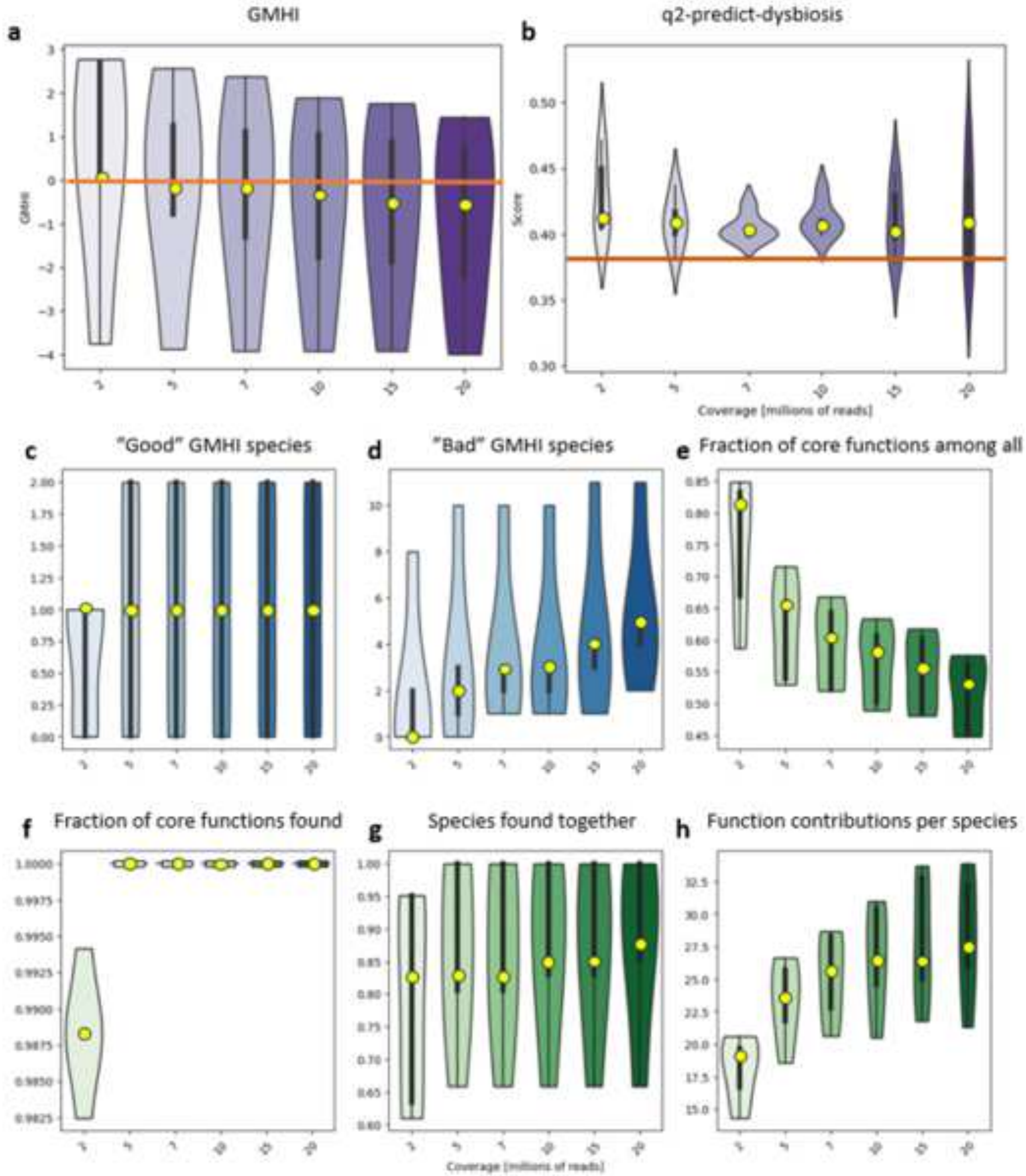

Figure 1

Click here to access/download;Figure;Figure 1.png

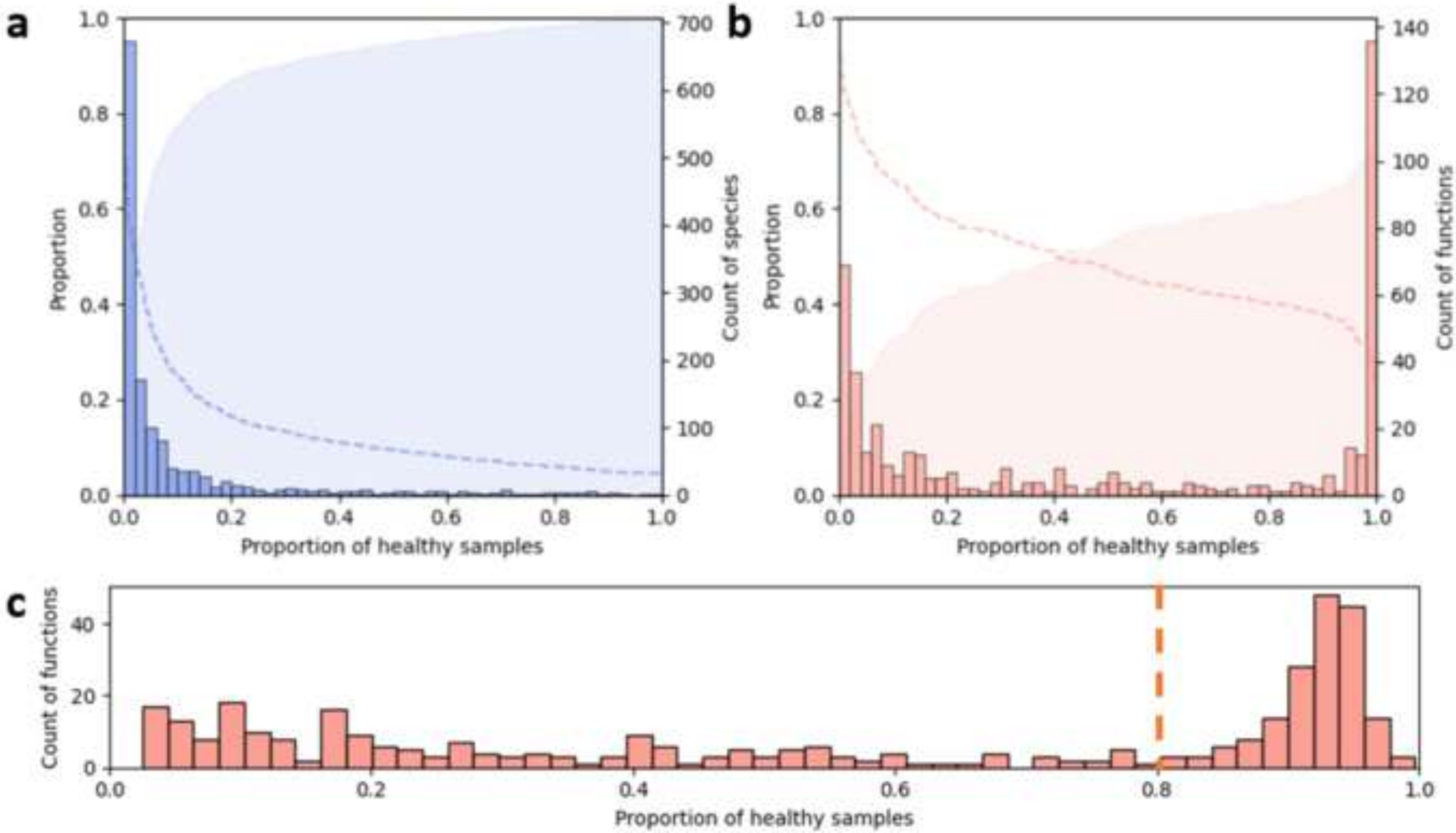

Figure 2

Click here to access/download;Figure;Figure 2.png

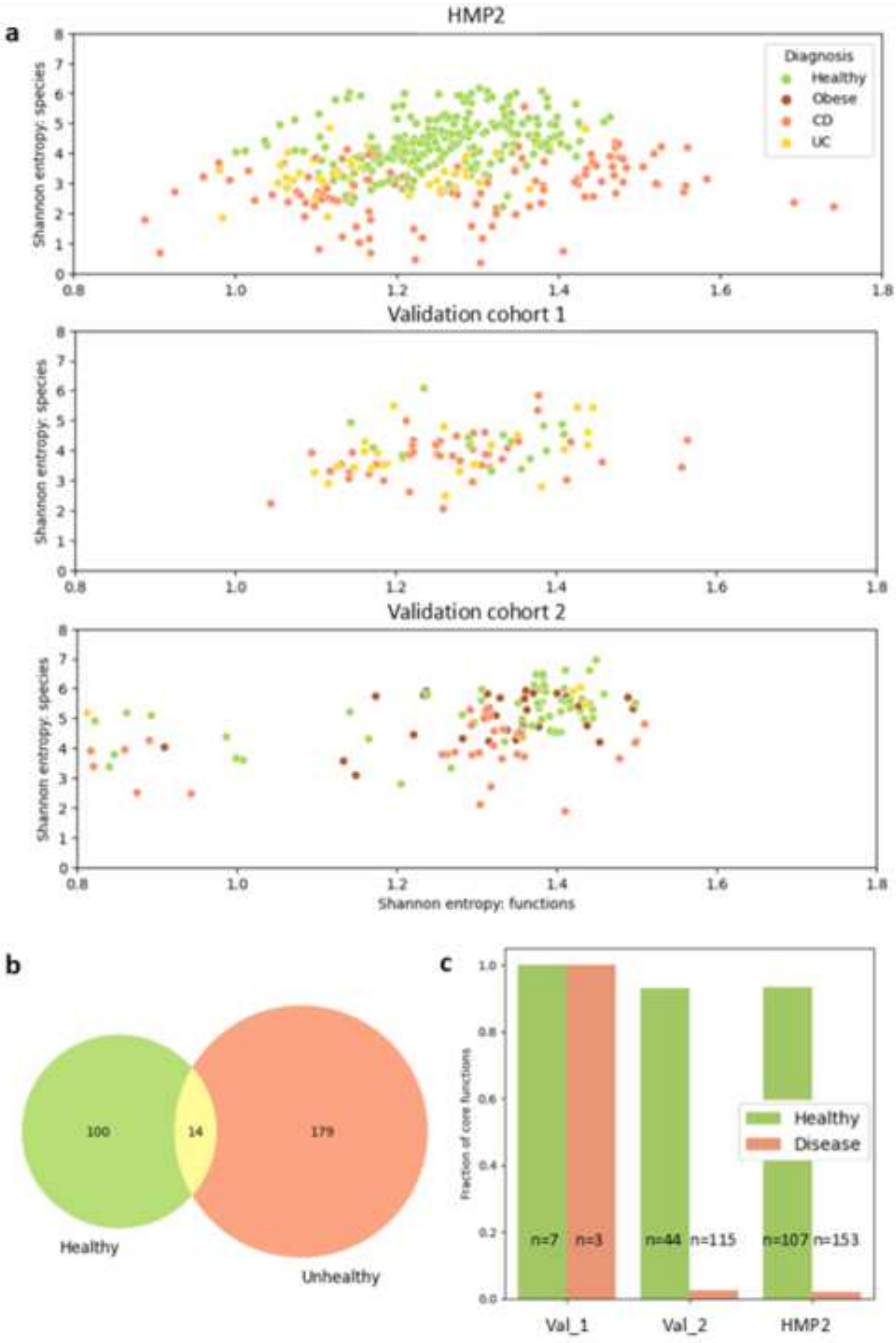

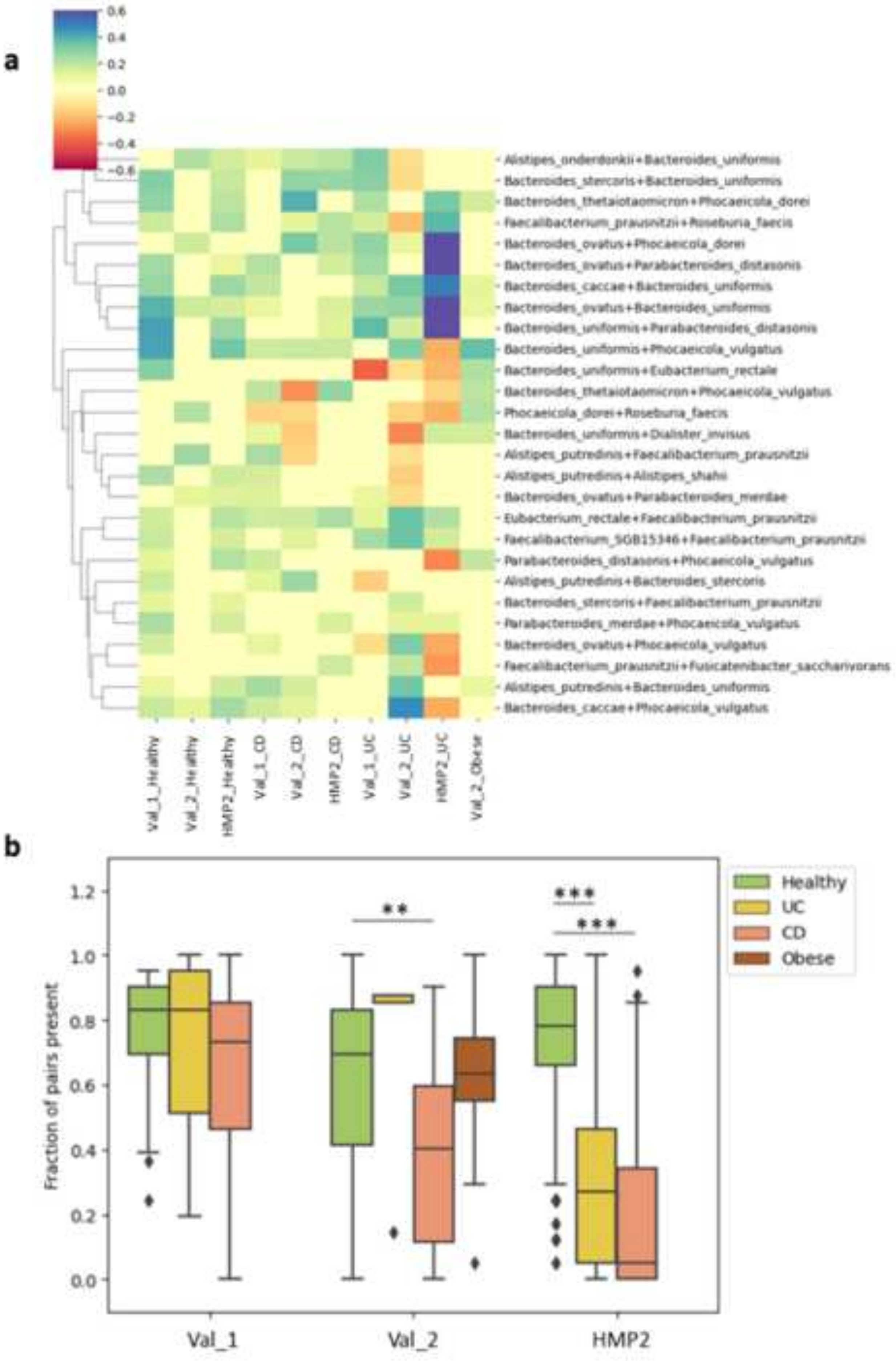
a
0.6
0.4
0.2
0.0
−0.2
−0.4
−0.6
Alistipes_onderdonkii+Bacteroides_uniformis
Bacteroides_stercoris+Bacteroides_uniformis
Bacteroides_thetaiotaomicron+Phocaeicola_dorei
Faecalibacterium_prausnitzii+Roseburia_faecis
Bacteroides_ovatus+Phocaeicola_dorei
Bacteroides_ovatus+Parabacteroides_distasonis
Bacteroides_caccae+Bacteroides_uniformis
Bacteroides_ovatus+Bacteroides_uniformis
Bacteroides_uniformis+Parabacteroides_distasonis
Bacteroides_uniformis+Phocaeicola_vulgatus
Bacteroides_uniformis+Eubacterium_rectale
Bacteroides_thetaiotaomicron+Phocaeicola_vulgatus
Phocaeicola_dorei+Roseburia_faecis
Bacteroides_uniformis+Dialister_invisus
Alistipes_putredinis+Faecalibacterium_prausnitzii
Alistipes_putredinis+Alistipes_shahii
Bacteroides_ovatus+Parabacteroides_merdae
Eubacterium_rectale+Faecalibacterium_prausnitzii
Faecalibacterium_SGB15346+Faecalibacterium_prausnitzii
Parabacteroides_distasonis+Phocaeicola_vulgatus
Alistipes_putredinis+Bacteroides_stercoris
Bacteroides_stercoris+Faecalibacterium_prausnitzii
Parabacteroides_merdae+Phocaeicola_vulgatus
Bacteroides_ovatus+Phocaeicola_vulgatus
Faecalibacterium_prausnitzii+Fusicatenibacter_saccharivorans
Alistipes_putredinis+Bacteroides_uniformis
Bacteroides_caccae+Phocaeicola_vulgatus
Val_1_Healthy
Val_2_Healthy
HMP2_Healthy
Val_1_CD
Val_2_CD
HMP2_CD
Val_1_UC
Val_2_UC
HMP2_UC
Val_2_Obese
b
Fraction of pairs present
1.2
1.0
0.8
0.6
0.4
0.2
0.0
**
***
***
Healthy
UC
CD
Obese
Val_1
Val_2
HMP2

Click here to access/download
Supplementary Material
Manuscript_supplement_new.docx

Personal Cover

Click here to access/download;Personal Cover;Q2PD_revised_letter.pdf

Prof. Paweł P Łabaj, PhD DSc
Head of Bioinformatics RG
Deputy Director for General Affairs

Małopolska Centre of Biotechnology
Jagiellonian University
ul. Gronostajowa 7A
30-387 Kraków, Poland
e: pawel.labaj@uj.edu.pl

December 3, 2024

JAGIELLONIAN
UNIWERSITY
IN KRAKÓW

MALOPOLSKA
CENTRE
OF BIOTECHNOLOGY

Dear Editor Dr Hans Zauner,

Dear Reviewers,

while there were no requested revisions we disagreed with, we would like to use this opportunity to thank you for your valuable feedback and give a brief overview of the substantial changes we have made to our manuscript since the last revision round.

After reading your comments, we felt inspired to expand our method beyond IBD and to make our benchmark unassailable, leaving no room for contention. We have emphasized our approach to developing the Q2PD solely on healthy individuals, which we tested on a comprehensive set consisting of 30 cohorts with various diseases. By applying a range of benchmarking approaches, we have shown that, overall, our index was superior to the other methods. This was particularly true when we compared all approaches on an unseen COVID-19 dataset, where only our index successfully identified diseased individuals. Furthermore, thanks to training the Q2PD on diverse data, we have made it exceptionally robust to sequencing depth.

We found your questions about the importance of each parameter of the Q2PD in the context of different diseases particularly insightful. Notably, having calculated feature importances for each cohort, we observed that diseases such as IBD would be classified well with taxonomy-based parameters, while the presence of taxonomic features was detrimental to the performance of the Q2PD on the COVID-19 dataset. This lead us to a fascinating hypothesis that perhaps this could be explained by the location of the disease, where taxonomy-based features could be linked to alterations originating in the gut, while the microbiome would change functionally if it was influenced by a disease originating elsewhere. While this finding is beyond the scope of the manuscript, we believe it could be of utmost importance to the further understanding of the microbiome health and disease, and a direction we would love to follow in our future work.

We would like to once again sincerely thank you for your feedback, and emphasize how greatly it influenced our newest version of the manuscript. We are very excited to present to you our updated work, and are really looking forward to your opinions. We hope you find the results as exciting as we do and recognize their importance, especially now as the topic is gaining increased attention in recently published original articles and reviews.

Sincerely,

Łabaj Paweł

Paweł P. Łabaj

Address:
Gronostajowa 7a st.
30-387 Kraków
