# Supplementary material for: Healthy microbiome—moving towards functional interpretation
Source: Gigascience. 2025 Mar 21;14:giaf015. doi: 10.1093/gigascience/giaf015 (PMC11927397; doi:10.1093/gigascience/giaf015)
Supplement: giaf015_Response_to_Reviewer_Comments_Original_Submission [file giaf015_response_to_reviewer_comments_original_submission.pdf]

Author's Response To Reviewer Comments

Thank you very much for your insightful comments. Please find our answers below.

Reviewer #1: The manuscript proposes a new method to distinguish between healthy and diseased human gut microbiomes. The topic is timely, as to date, there is no consensus on what constitutes a healthy microbiome. The key conceptual advance of this study is the integration of functional microbiome features to define health. Their new computational approach, q2-predict-dysbiosis (Q2PD), is open source and available on GitHub.

While the manuscript is conceptually innovative and interesting for the scientific community, there are several major limitations in the current version of this study.

#1. To develop the Q2PD, they define features associated with health by comparing it with microbiome samples from IBD patients. There are many more non-healthy/dysbiotic phenotypes beyond IBD, therefore it is not accurate to use IBD as synonymous of dysbiosis as done throughout this version of the paper.
Thank you for this insightful comment. It seems that our process has not been clear – the index has been developed on the healthy cohort only, and the IBD samples were used to validate our assumptions about the healthy microbiomes. Therefore, ”dysbiosis” in our case refers to the alteration of the microbiome healthy state. We have since revised our wording and ensured this does not cause any further confusion.

#2 The study initially tests the performance of Q2PD against other gut microbiome health indexes (GMHI and hiPCA) using the same data that was used to select the health-associated features of Q2PD. Model performance should be assessed on independent data. On a separate analysis, they do use different datasets (from GMHI and hiPCA), but these datasets seem to be incomplete - GMHI and hiPCA publications have included 10 or more disease categories, and it is unclear why only 4 categories are shown in this study.
Thank you for pointing this out. Following your comment, we have expanded our test dataset to as many as 31 diseases in total (Supplementary Figure 6). We do not include all samples for the following reasons: incomplete metadata, use of antibiotics or other similar drugs, repeats from the same patients, young age (newborns), or healthy samples not being fully healthy (high BMI, chronic conditions). We have included this information in the Methods (lines 405-412). The test data not only contains datasets that the GMHI and hiPCA had originally been developed on but also additional, unseen by any method data.

#3 While Q2PD does provide visible improvements in differentiating some diseases from healthy phenotypes, the accuracy and sensitivity of Q2PD isn't clear. To adopt Q2PD, I would like to know what are the chances that the classification results will be correct.
Thank you. We have since added information about the accuracy and AUC scores as our benchmark in the main body of the manuscript. The results are presented for the whole dataset as well as individually for each disease (Figure 5a, Supplementary Figure 6). In terms of the classification on the level of an individual samples, higher values (closer to 1) indicate increasing confidence of the index that the sample is healthy. We have added this information to the Methods (lines 413).

#4 There is very little documentation on how to use Q2PD. What are the expect outputs for example, do we need to chose a threshold to define health? Is the method completely dependent on Humann and Metaphlan outputs, or other formats are accepted? The test data contain some samples with zero counts. I got an error when trying it with the test data (ValueError: node array from the pickle has an incompatible dtype…).
Thank you. We have now improved the code and the documentation. We fixed bugs, added requirements and information about the output – which answer all of your questions. Currently the only accepted input is Metaphlann and Humann, but as we state in our discussion, we are planning to expand to other inputs in the future. We are also planning to wrap-up the method as a QIIME2 plugin to make it more accessible to a wider range of researchers.

Therefore, I recommend including a range of disease categories to develop Q2PD and use independent

datasets to validate the model in terms of accuracy and sensitivity. Alternatively, consider focusing this contribution on IBD. Making the code more user friendly will drastically increase the adoption of Q2PD by the community.
We would like to sincerely thank you for your feedback, which substantially improved our manuscript. It not only allowed us to work on the weakest parts but also inspired us to re-write most sections and greatly improve the work overall.

Please also use page and line numbers when submitting the next version.
Thank you, we have added those as requested.

Other suggestions:

Abstract: I recommend replacing 'attributed' with 'linked', as 'attributed' suggests that dysbiosis may be causing (rather than reflecting) disease.
Thank you, we have made the necessary replacement.

Results:
Please indicate what it is meant by 'function' here - it will be good to clarify that this method uses Metaphlan's read-based approach to identify metabolic pathways. What is used, pathway completeness or abundance?
Thank you. Indeed, we used Metaphlan and Humann, specifically the MetaCyc functional annotations. We performed additional filtering and set the following thresholds: species abundance >= 0.1%, pathway coverage >= 20% and function abundance >= 0.01% - with any features below changed to 0. This has now been included in the Methods (lines 4011-412).

Results regarding Figure 3a are difficult to interpret. Is 'non-negatively correlated' the same as 'positively correlated'? What does the colour gradient represent - their abundance in those groups, or the strength of their correlation?
Thank you for pointing this out. Non-negative correlation indicates that a pairing was not negatively correlated in any healthy cohort. The colour represents the strength of the SparCC correlation between the species. We have added this description to the figure caption (Figure 3).

"We observed that the prevalence of the pairs positively correlated in health was higher than in a number of disease-associated groups (Figure 3b)" . This is a very generalised statement considering that only half of the comparisons were significant. How co-occurring species were selected?
Thank you for asking. The species were selected based on the MDFS algorithm, which searches for relevant features in the context of a decision variable (in our case, disease). This must have not been clear, so we have modified the text accordingly (lines 174-175). What we were looking for here were trends – if we managed to find a parameter which would perfectly separate the groups, there would be no need for an index. The separation was strong enough, however, to be informative and to form stand-alone model parameter.

"To test this, we compared the contributions of MDFS-identified species to "core functions" in different groups (Supplementary Figure 4)." How was this comparison made, based on species correlations? The caption of these figures could include more detail - it just says 'Top species contributions to functions.' but how do you define 'top' ? What do the colours represent?
Thank you for inquiring. The species contributions were based on the stratified output of HUMAnN. For every cohort, the sum of the contributions for all samples was calculated and contributions with the greatest summed abundances are shown. The final values have been normalized to values between 0 and 1 to compare the strength of the correlations between the groups. We have now added this information to the figure caption (Supplementary Figure 4).

'This finding was congruent with our earlier suspicions of functional plasticity; modulation of function and thus altered connectivity in the interaction network, shifting
towards less abundant, non-core functions upon perturbation of homeostasis.' This is reasonable, but I don't understand how you can draw this conclusion from these figures where there seems to be no significant difference between health and disease.

Thank you for your comment. As we pointed in one of our answers above, finding a feature which would produce a statistically significant difference for all cases would indicate no need for a more complex index. However, as microbiome alterations in dysbiosis and disease are multidimensional and can take many forms, an index composed of multiple informative parameters is required. We have adjusted the wording in the end of the paragraph to make this clearer (lines 195-197).

Section 'Testing q2-predict-dysbiosis, GMHI and hiPCA accuracy of prediction for healthy and IBD individuals'

What is the difference between fraction of "core functions" found the fraction of "core functions" among all functions?
Thank you. The first one refers to the fraction of all core functions we pre-defined found, whereas the latter determines the percentage of the functions found in the sample being core. We have not only changed the wording within the manuscript (lines 200-205) but also added Table 1 with additional information.

"Most importantly, Q2PD produced visually the highest scores for all healthy in comparison to unhealthy cohorts" . This was not statistically significant. In fact, GMHI finds more significant differences between health and disease than Q2PD.
Thank you. We have since re-trained our index and the results are now different. Our index demonstrated the strongest statistically significant separation between healthy and Crohn’s disease or ulcerative colitis individuals across all methods evaluated (Figure 4a). Alongside hiPCA, it was one of only two approaches to achieve a statistically significant distinction in the Nielsen_2014 cohort. Overall, both our index and hiPCA exhibited comparable levels of accuracy and AUC across the three cohorts, outperforming the other methods by a notable margin. In addition, when index values were used as features in the Boruta algorithm, with health status (0/1) as the target variable, our index emerged with the highest mean and summed importance across all cohorts (Figure 4c).

Sup. Figure 7 - would be informative to add the name/description of these metabolites not just their ID).
Thank you – this section has now been removed because it was adding unnecessary complexity.

'Although the threshold of 0.6 as determinant of health by the Q2PD was not applicable to the new datasets'. Does the threshold to define health with Q2PD change depending on the dataset? What are the implications of this for the applicability of this index?
Thank you. We have since re-trained our index on multiple cohorts, which resulted in a unified threshold for all samples (threshold = 0.38). This has substantially improved the applicability of our method to new cohorts. We have included this information in the documentation of our method on Github.

Effects of sequencing depth - this is a very good addition to the paper, the effects of sequencing depth can be profound but are ignored in most studies, so I commend the authors for doing this here. It would be even better, in my opinion, if this was done with the same datasets used to test/compare Q2PD with other methods, as using a different dataset here adds a new layer of confounding factors.
Thank you. There were two reasons for choosing healthy samples from the COVID-19 dataset. Firstly, some of the datasets used in the subsequent sections had been used to train the GMHI or the hiPCA. We chose COVID-19, an independent Polish cohort, to make sure that none of the indices were in an advantageous position. Secondly, the COVID-19 cohort had a substantial sequencing depth – with some samples exceeding 50 million reads. This allowed us to evaluate the effects of a large degree of rarefaction. The two reasons resulted in a strict and unbiased benchmark.

'the GMHI and the hiPCA produced the opposite trend, wrongly indicating patient recovery.' The difference here is striking, what is driving this trend?
Thank you. Following your question, we examined importance of each parameter of the Q2PD separately for each cohort (Figure 5). The results revealed a large variability within the features contributing to the score for each cohort and disease the most. Your insightful comment has led us to further conclusions, however. We hypothesize that diseases, which originate in the gut, are more depedent on taxonomy-based indices (as they most often arise due to the presence of pathogens). On the other hand, diseases originating outside of the gut would have more impact on its functional composition. We prove this in the subsequent COVID-19 section, where we show that the presence of taxonomic features decreases the

performance of the Q2PD on the COVID-19 cohort (Figure 6 b,c). Finally, we have also modified our software, which is now possible to run in two modes: with and without the taxonomy-based indices.

The Gut Microbiome Wellness Index 2 (GMWI2) is now published. I don't think it needs to be part of the benchmarking, but it could be acknowledged/cited here.
Thank you, we have now added the citation.

Methods:
More information on how the data was processed is needed - how were the abundance tables normalized? Which output from Humann was used for downstream analyses?
Thank you. We have now added this information to the Methods (lines 405-412) and included all the scripts on Github.

To ensure reproducibility, please provide the scripts/code used for analyses and figures.
Thank you. Our Github now contains scripts required to re-run our analyses, as well as information about how to run different tools.

Reviewer #2: In this study, the authors present a novel metagenomic health index designed to differentiate between healthy and unhealthy microbiomes. This area of research is crucial for developing a non-invasive, cost-effective method to assess patient health status. However, I have several suggestions that I believe will enhance the study and address some key points.

Main Comments:
1.) The study would benefit from additional post-analysis to provide greater depth. Although the authors applied their approach to several diseases, they did not elaborate on the significance of individual microbiome features across different diseases. For instance, the GMHI parameters were identified as least important in IBD—does this observation hold universally across all diseases analysed?
Thank you for your feedback. Since then, we have expanded our test dataset to include 30 diseases and extended our post-analysis. In response to your question, we assessed the importance of each Q2PD parameter separately for each cohort (Figure 5). This analysis revealed significant variability in the features contributing most to the score across different cohorts and diseases. Your insightful comment has inspired further conclusions. We propose that diseases originating in the gut are more reliant on taxonomy-based indices, as they often result from the presence of specific pathogens. Conversely, diseases originating outside the gut are likely to have a greater impact on its functional composition. We demonstrate this in the subsequent COVID-19 section, showing that the inclusion of taxonomic features reduces the Q2PD's performance in the COVID-19 cohort (Figure 6b,c). Finally, we have updated our software, which can now operate in two modes: with or without taxonomy-based indices.

2.) The index Q2D performed worse in AGP1 compared to HMP2 and AGP2. Is there a specific reason for this discrepancy? For example, does the index underperform in the heterogeneous functional landscape presented in AGP1 (Figure 2C)? An explanation for the reduced performance in this cohort would provide valuable insights into the method's performance under varying conditions.
Thank you for inquiring. We have since re-trained our index, and now our method is able to distinguish between healthy and IBD individuals from all cohorts correctly. In two out of three cohorts, the separation is statistically significant (Figure 4a).

3.) It would be beneficial to make all processed data and relevant scripts available in a GitHub repository to ensure that the results presented in the paper can be replicated by other researchers.
Thank you for this advice. Our Github now contains scripts required to re-run our analyses, as well as information about how to run different tools.

4.) When attempting to run the script available at https://github.com/Kizielins/q2-predict-dysbiosis, I encountered an error related to the scikit-learn version. The script appears to be compatible with version 1.2.2, whereas I was using version 1.4.2. Please consider updating the script or providing instructions for

resolving version compatibility issues.
Thank you for testing our method. We have since fixed the code and included an information about version requirements.

5.) The rationale behind considering only positive correlations when calculating the index is unclear. It would be helpful to clarify why negative correlations were excluded from the index calculations.
Thank you for pointing this out. The reason for including only positive correlations was searching for the co-occurrence of species, which we showed was greater in healthy than in disease samples (Figure 3b). The generalized results allowed us to transition to the level of an individual sample. Therefore, one of the parameters of the Q2PD is the co-occurrence of species pairs.

6.) In analysing longitudinal alterations, did the authors account for dependencies from previous time points Q2D index? If not, how do these longitudinal alterations differ from those observed in independent studies?
Thank you for asking. As our method has currently been only developed for cross-sectional data, we consider the correct identification of the expected longitudinal trend as an additional validation of our method. We are planning to develop our approach specifically for longitudinal datasets in the future.

7.) For each dataset analysed, additional details would be useful, such as the number of samples, species, functions, core functions, and the number of species remaining after applying the MDFS algorithm.
Thank you for this suggestion. A file with all calculated parameters for each sample has been added to our Gitlab as a part of the complete analysis. It can be found in the ”Data” folder under ”calculated_parameters_for_merged.txt”.

8.) On Page 13, the authors state that they chose GMHI as their benchmark because hiPCA and Shannon entropy produced worse results for the HMP2 cohort. However, Supplementary Table 3 indicates that Shannon entropy had a lower p-value than GMHI in the Mann-Whitney U test.
Thank you. We have since added all methods to our validation (Figure 5a).

Minor comments:

1) Page 11
Original: "Collecting information on feature importance at every iteration of the cross-validation procedure model, we consistently identified the two GMHI parameters as the least important (Figure 5b)."
Suggested: "Collecting information on feature importance at every iteration of the cross-validation procedure model, we consistently identified the two GMHI parameters as the least important (Figure 4b??)."
Thank you for finding the errors. We have since modified the feature importance section (Figure 5b) and removed this section.

2) Page 12
Original: "Most importantly, Q2PD produced visually the highest scores for all healthy in comparison to unhealthy cohorts."
Suggested: "Most importantly, Q2PD produced visually the highest median?? scores for all healthy in comparison to unhealthy cohorts."
Thank you, we have since removed this section.

3) Page 12
Original: "Q2PD was also the only index to produce a statistically significant difference between Healthy and Obese in HMP2"
Suggested: "Q2PD was also the only index to produce a statistically significant difference between Healthy and Obese in AGP2??"
Thank you, we have since removed this section.

4) Page 14
Original: "The Q2PD important in all datasets that were included in its training and validation, specifically AGP_1, AGP_2 and HMP2 (Table 1, Supplementary Figure 7)."

Suggested: "The Q2PD important in all datasets that were included in its training and validation, specifically AGP_1, AGP_2 and HMP2 (Table 1, Supplementary Figure 8??)."
Thank you, we have since removed this section.
