# Supplementary material for: Healthy microbiome—moving towards functional interpretation
Source: Gigascience. 2025 Mar 21;14:giaf015. doi: 10.1093/gigascience/giaf015 (PMC11927397; doi:10.1093/gigascience/giaf015)
Supplement: giaf015_Response_to_Reviewer_Comments_Revision_1 [file giaf015_response_to_reviewer_comments_revision_1.pdf]

Author's Response To Reviewer Comments

Thank you for the further feedback. We have now revised the Manuscript accordingly.
