# Supplementary material for: Healthy microbiome—moving towards functional interpretation
Source: Gigascience. 2025 Mar 21;14:giaf015. doi: 10.1093/gigascience/giaf015 (PMC11927397; doi:10.1093/gigascience/giaf015)
Supplement: giaf015_Reviewer_1_Report_Original_Submission — Vanessa Marcelino -- 9/8/2024 [file giaf015_reviewer_1_report_original_submission.pdf]

Reviewer Report

Title: Healthy microbiome - moving towards functional interpretation

Version: Original Submission **Date:** 9/8/2024

Reviewer name: Vanessa Marcelino

Reviewer Comments to Author:

The manuscript proposes a new method to distinguish between healthy and diseased human gut microbiomes. The topic is timely, as to date, there is no consensus on what constitutes a healthy microbiome. The key conceptual advance of this study is the integration of functional microbiome features to define health. Their new computational approach, q2-predict-dysbiosis (Q2PD), is open source and available on GitHub.While the manuscript is conceptually innovative and interesting for the scientific community, there are several major limitations in the current version of this study.#1. To develop the Q2PD, they define features associated with health by comparing it with microbiome samples from IBD patients. There are many more non-healthy/dysbiotic phenotypes beyond IBD, therefore it is not accurate to use IBD as synonymous of dysbiosis as done throughout this version of the paper.#2 The study initially tests the performance of Q2PD against other gut microbiome health indexes (GMHI and hiPCA) using the same data that was used to select the health-associated features of Q2PD. Model performance should be assessed on independent data. On a separate analysis, they do use different datasets (from GMHI and hiPCA), but these datasets seem to be incomplete - GMHI and hiPCA publications have included 10 or more disease categories, and it is unclear why only 4 categories are shown in this study.#3 While Q2PD does provide visible improvements in differentiating some diseases from healthy phenotypes, the accuracy and sensitivity of Q2PD isn't clear. To adopt Q2PD, I would like to know what are the chances that the classification results will be correct.#4 There is very little documentation on how to use Q2PD. What are the expect outputs for example, do we need to chose a threshold to define health? Is the method completely dependent on Humann and Metaphlan outputs, or other formats are accepted? The test data contain some samples with zero counts. I got an error when trying it with the test data (ValueError: node array from the pickle has an incompatible dtype…).Therefore, I recommend including a range of disease categories to develop Q2PD and use independent datasets to validate the model in terms of accuracy and sensitivity. Alternatively, consider focusing this contribution on IBD. Making the code more user friendly will drastically increase the adoption of Q2PD by the community.Please also use page and line numbers when submitting the next version.Other suggestions:Abstract: I recommend replacing 'attributed' with 'linked', as 'attributed' suggests that dysbiosis may be causing (rather than reflecting) disease.Results:Please indicate what it is meant by 'function' here - it will be good to clarify that this method uses Metaphlan's read-based approach to identify metabolic pathways. What is used, pathway completeness or abundance?Results regarding Figure 3a are difficult to interpret. Is 'non-negatively correlated' the same as 'positively correlated'? What does the colour gradient represent - their abundance in those groups, or the strength of their correlation?"We observed that the prevalence of the pairs positively correlated in health was higher than in a number of disease-associated groups (Figure 3b)" . This is a very generalised statement

considering that only half of the comparisons were significant. How co-occurring species were selected?"To test this, we compared the contributions of MDFS-identified species to "core functions" in different groups (Supplementary Figure 4)." How was this comparison made, based on species correlations? The caption of these figures could include more detail - it just says 'Top species contributions to functions.' but how do you define 'top' ? What do the colours represent?'This finding was congruent with our earlier suspicions of functional plasticity; modulation of function and thus altered connectivity in the interaction network, shiftingtowards less abundant, non-core functions upon perturbation of homeostasis.' This is reasonable, but I don't understand how you can draw this conclusion from these figures where there seems to be no significant difference between health and disease.Section 'Testing q2-predict-dysbiosis, GMHI and hiPCA accuracy of predictionfor healthy and IBD individuals'What is the difference between fraction of "core functions" found the fraction of "core functions" among all functions?"Most importantly, Q2PD produced visually the highest scores for all healthy incomparison to unhealthy cohorts" . This was not statistically significant. In fact, GMHI finds more significant differences between health and disease than Q2PD.Sup. Figure 7 - would be informative to add the name/description of these metabolites not just their ID).'Although the threshold of 0.6 as determinant of health by the Q2PD was not applicable to the new datasets'. Does the threshold to define health with Q2PD change depending on the dataset? What are the implications of this for the applicability of this index?Effects of sequencing depth - this is a very good addition to the paper, the effects of sequencing depth can be profound but are ignored in most studies, so I commend the authors for doing this here. It would be even better, in my opinion, if this was done with the same datasets used to test/compare Q2PD with other methods, as using a different dataset here adds a new layer of confounding factors.'the GMHI and the hiPCA produced the opposite trend, wrongly indicating patient recovery.' The difference here is striking, what is driving this trend?The Gut Microbiome Wellness Index 2 (GMWI2) is now published. I don't think it needs to be part of the benchmarking, but it could be acknowledged/cited here.Methods:More information on how the data was processed is needed - how were the abundance tables normalized? Which output from Humann was used for downstream analyses?To ensure reproducibility, please provide the scripts/code used for analyses and figures.

Level of Interest

Please indicate how interesting you found the manuscript: Choose an item.

Quality of Written English

Please indicate the quality of language in the manuscript: Choose an item.

Declaration of Competing Interests

Please complete a declaration of competing interests, considering the following questions:

- Have you in the past five years received reimbursements, fees, funding, or salary from an organisation that may in any way gain or lose financially from the publication of this manuscript, either now or in the future?
- Do you hold any stocks or shares in an organisation that may in any way gain or lose financially from the publication of this manuscript, either now or in the future?
- Do you hold or are you currently applying for any patents relating to the content of the manuscript?
- Have you received reimbursements, fees, funding, or salary from an organization that holds or has applied for patents relating to the content of the manuscript?
- Do you have any other financial competing interests?
- Do you have any non-financial competing interests in relation to this paper?

If you can answer no to all of the above, write 'I declare that I have no competing interests' below. If your reply is yes to any, please give details below.

I declare that I have no competing interests.

I agree to the open peer review policy of the journal. I understand that my name will be included on my report to the authors and, if the manuscript is accepted for publication, my named report including any attachments I upload will be posted on the website along with the authors' responses. I agree for my report to be made available under an Open Access Creative Commons CC-BY license (http://creativecommons.org/licenses/by/4.0/). I understand that any comments which I do not wish to be included in my named report can be included as confidential comments to the editors, which will not be published.

Choose an item.

To further support our reviewers, we have joined with Publons, where you can gain additional credit to further highlight your hard work (see: https://publons.com/journal/530/gigascience). On publication of this paper, your review will be automatically added to Publons, you can then choose whether or not to claim your Publons credit. I understand this statement.

Yes Choose an item.
