# Supplementary material for: Healthy microbiome—moving towards functional interpretation
Source: Gigascience. 2025 Mar 21;14:giaf015. doi: 10.1093/gigascience/giaf015 (PMC11927397; doi:10.1093/gigascience/giaf015)
Supplement: giaf015_Reviewer_2_Report_Original_Submission — Saritha Kodikara -- 9/13/2024 [file giaf015_reviewer_2_report_original_submission.pdf]

Reviewer Report

Title: Healthy microbiome - moving towards functional interpretation

Version: Original Submission **Date:** 9/13/2024

Reviewer name: Saritha Kodikara

Reviewer Comments to Author:

In this study, the authors present a novel metagenomic health index designed to differentiate between healthy and unhealthy microbiomes. This area of research is crucial for developing a non-invasive, cost-effective method to assess patient health status. However, I have several suggestions that I believe will enhance the study and address some key points.Main Comments:1.) The study would benefit from additional post-analysis to provide greater depth. Although the authors applied their approach to several diseases, they did not elaborate on the significance of individual microbiome features across different diseases. For instance, the GMHI parameters were identified as least important in IBDâ€”does this observation hold universally across all diseases analysed?2.) The index Q2D performed worse in AGP1 compared to HMP2 and AGP2. Is there a specific reason for this discrepancy? For example, does the index underperform in the heterogeneous functional landscape presented in AGP1 (Figure 2C)? An explanation for the reduced performance in this cohort would provide valuable insights into the method's performance under varying conditions.3.) It would be beneficial to make all processed data and relevant scripts available in a GitHub repository to ensure that the results presented in the paper can be replicated by other researchers.4.) When attempting to run the script available at https://github.com/Kizielins/q2-predict-dysbiosis, I encountered an error related to the scikit-learn version. The script appears to be compatible with version 1.2.2, whereas I was using version 1.4.2. Please consider updating the script or providing instructions for resolving version compatibility issues.5.) The rationale behind considering only positive correlations when calculating the index is unclear. It would be helpful to clarify why negative correlations were excluded from the index calculations.6.) In analysing longitudinal alterations, did the authors account for dependencies from previous time points Q2D index? If not, how do these longitudinal alterations differ from those observed in independent studies?7.) For each dataset analysed, additional details would be useful, such as the number of samples, species, functions, core functions, and the number of species remaining after applying the MDFS algorithm.8.) On Page 13, the authors state that they chose GMHI as their benchmark because hiPCA and Shannon entropy produced worse results for the HMP2 cohort. However, Supplementary Table 3 indicates that Shannon entropy had a lower p-value than GMHI in the Mann-Whitney U test.Minor comments:1) Page 11Original: "Collecting information on feature importance at every iteration of the cross-validation procedure model, we consistently identified the two GMHI parameters as the least important (Figure 5b)."Suggested: "Collecting information on feature importance at every iteration of the cross-validation procedure model, we consistently identified the two GMHI parameters as the least important (Figure 4b??)."2) Page 12Original: "Most importantly, Q2PD produced visually the highest scores for all healthy in comparison to unhealthy cohorts."Suggested: "Most importantly, Q2PD produced visually the highest median?? scores for all healthy in comparison to unhealthy cohorts."3)

Page 12Original: "Q2PD was also the only index to produce a statistically significant difference between Healthy and Obese in HMP2"Suggested: "Q2PD was also the only index to produce a statistically significant difference between Healthy and Obese in AGP2??"4) Page 14Original: "The Q2PD important in all datasets that were included in its training and validation, specifically AGP_1, AGP_2 and HMP2 (Table 1, Supplementary Figure 7)."Suggested: "The Q2PD important in all datasets that were included in its training and validation, specifically AGP_1, AGP_2 and HMP2 (Table 1, Supplementary Figure 8??)."

Level of Interest

Please indicate how interesting you found the manuscript: Choose an item.

Quality of Written English

Please indicate the quality of language in the manuscript: Choose an item.

Declaration of Competing Interests

Please complete a declaration of competing interests, considering the following questions:

- Have you in the past five years received reimbursements, fees, funding, or salary from an organisation that may in any way gain or lose financially from the publication of this manuscript, either now or in the future?
- Do you hold any stocks or shares in an organisation that may in any way gain or lose financially from the publication of this manuscript, either now or in the future?
- Do you hold or are you currently applying for any patents relating to the content of the manuscript?
- Have you received reimbursements, fees, funding, or salary from an organization that holds or has applied for patents relating to the content of the manuscript?
- Do you have any other financial competing interests?
- Do you have any non-financial competing interests in relation to this paper?

If you can answer no to all of the above, write 'I declare that I have no competing interests' below. If your reply is yes to any, please give details below.

'I declare that I have no competing interests

I agree to the open peer review policy of the journal. I understand that my name will be included on my report to the authors and, if the manuscript is accepted for publication, my named report including any attachments I upload will be posted on the website along with the authors' responses. I agree for my report to be made available under an Open Access Creative Commons CC-BY license (http://creativecommons.org/licenses/by/4.0/). I understand that any comments which I do not wish to

be included in my named report can be included as confidential comments to the editors, which will not be published.

Choose an item.

To further support our reviewers, we have joined with Publons, where you can gain additional credit to further highlight your hard work (see: https://publons.com/journal/530/gigascience). On publication of this paper, your review will be automatically added to Publons, you can then choose whether or not to claim your Publons credit. I understand this statement.

Yes Choose an item.
